# DNA barcodes from four loci provide poor resolution of taxonomic groups in the genus *Crataegus*

**DOI:** 10.1093/aobpla/plv045

**Published:** 2015-04-29

**Authors:** Mehdi Zarrei, Nadia Talent, Maria Kuzmina, Jeanette Lee, Jensen Lund, Paul R. Shipley, Saša Stefanović, Timothy A. Dickinson

**Affiliations:** 1 The Centre for Applied Genomics, The Hospital for Sick Children, Peter Gilgan Centre for Research and Learning, 686 Bay St, Toronto, ON, Canada M5G 0A4; 2 Green Plant Herbarium, Department of Natural History, Royal Ontario Museum, 100 Queen's Park, Toronto, ON, Canada M5S 2C6; 3 Canadian Centre for DNA Barcoding, 50 Stone Road East, Guelph, ON, Canada N1G 2W1; 4 109 Lakeshore Ave., Edgewood, BC, Canada V0G 1J0; 5 Department of Chemistry, I.K. Barber School of Arts and Sciences, The University of British Columbia Okanagan, 3333 University Way, Kelowna, BC, Canada V1V 1V7; 6 Department of Ecology and Evolutionary Biology, University of Toronto, 25 Willcocks Street, Toronto, ON, Canada M5S 3B2; 7 Department of Biology, University of Toronto Mississauga, 3359 Mississauga Road, Mississauga, ON, Canada L5L 1C6

**Keywords:** Gametophytic apomixis, hawthorn, hybridization, ITS2, Maleae, natural health products, plastid DNA, polyploidy, Rosaceae

## Abstract

DNA barcodes can facilitate identification of organisms especially when morphological characters are limited or unobservable. To what extent this potential is realized in specific groups of plants remains to be determined. Libraries of barcode sequences from well-studied authoritatively identified plants represented by herbarium voucher specimens are needed in order for DNA barcodes to serve their intended purpose, where this is possible, and to understand the reasons behind their failure to do so, when this occurs. We evaluated four loci, widely regarded as universal DNA barcodes for plants, for their utility in hawthorn species identification. Three plastid regions, *mat*K, *rbcL*a and *psb*A*-trn*H, and the internal transcribed spacer 2 (ITS2) of nuclear ribosomal DNA discriminate only some of the species of *Crataegus* that can be recognized on the basis of their morphology etc. This is, in part, because in Rosaceae tribe Maleae most individual plastid loci yield relatively little taxonomic resolution and, in part, because the effects of allopolyploidization have not been eliminated by concerted evolution of the ITS regions. Although individual plastid markers provided generally poor resolution of taxonomic groups in *Crataegus*, a few species were notable exceptions. In contrast, analyses of concatenated sequences of the 3 plastid barcode loci plus 11 additional plastid loci gave a well-resolved maternal phylogeny. In the ITS2 tree, different individuals of some species formed groups with taxonomically unrelated species. This is a sign of lineage sorting due to incomplete concerted evolution in ITS2. Incongruence between the ITS2 and plastid trees is best explained by hybridization between different lineages within the genus. In aggregate, limited between-species variation in plastid loci, hybridization and a lack of concerted evolution in ITS2 all combine to limit the utility of standard barcoding markers in *Crataegus*. These results have implications for authentication of hawthorn materials in natural health products.

## Abstract (precis)

The leaves and fruits of some of the approximately 230 species of hawthorn (*Crataegus*) yield natural health products with significant therapeutic effects on symptoms of cardiovascular disease. DNA barcoding could be a valuable tool for authenticating these products, but only a small fraction of the 93 taxa that we examined were distinguished, even though all major clades and eight out of ten taxonomic sections of the genus were included. DNA barcoding as currently practised thus has limited utility in *Crataegus*. Hybridization, lineage sorting due to incomplete concerted evolution in ITS2, and limited variation in plastid loci are implicated.

## Introduction

The main aim of DNA barcoding is to identify an unknown organism by comparing a DNA sequence from the unknown with records in a database of identified sequences, based on some measure of genetic similarity (Hebert *et al*. 2003*a*; Chase *et al*. 2005; Ross *et al*. 2008; Seberg and Petersen 2009; Hollingsworth *et al*. 2011; Dong *et al*. 2012) or combination of diagnostic sequence characters (Reid *et al*. 2011; Weitschek *et al*. 2013). The Barcode of Life Data Systems (BOLD) database (Ratnasingham and Hebert 2007) plays an important role as a centralized and curated depository for DNA barcode sequences that is effective in archiving and making accessible detailed voucher information for each organism from which the sequence was obtained. The Consortium for the Barcode of Life (CBOL) Plant Barcoding Group (2009) identified two plastid protein-coding loci, *mat*K and *rbcL*, as the barcode regions for generating a library of identified sequences for plants. Additional discrimination on species-level taxonomy was proved by using supplementary markers: plastid *psb*A-*trn*H intergenic spacer and internal transcribed spacer 2 (ITS2) from nuclear ribosomal DNA (Kress *et al*. 2009; Chen *et al*. 2010). The usefulness and challenges of using different plant DNA barcodes were summarized by Hollingsworth *et al*. (2011). It remains to be seen to what extent these markers can assist identification in closely related plant taxa that are difficult to identify because, for example, there is a limited number of characters, not all of which are necessarily present at the phenological stage of a given specimen.

Hawthorns (*Crataegus*, Rosaceae tribe Maleae) are frequent and often locally abundant throughout the North Temperate Zone in high light intensity habitats where the moisture regime permits the establishment of woody vegetation. Within the Rosaceae subfamily Amygdaloideae the genus is relatively large (Dickinson *et al*. 2007), with 50–100 Old World species (Christensen 1992), and 150–200 New World ones (Phipps 2015). Species identification depends on a suite of characters; some of which can only be recorded at the time of flowering, while others must be observed in fruiting material. In addition, identification has been further complicated by the much larger number of species that have been described in *Crataegus*, for which comprehensive synonymies have only recently become available (Christensen 1992; Phipps 2015).

Hawthorn fruits are edible and a few species are cultivated for their fruits in East Asia (*C. pinnatifida*), Europe (*C. azarolus*, *C. germanica*) and in the New World (*C. mexicana*, *C. opaca*). Hawthorns are also used in traditional medicine in all of these places, and there is a sizable market for natural health products (NHPs) made from hawthorn leaves, flowers and fruits (Edwards *et al*. 2012). Hawthorn chemistry is fairly well known, but most species studied are Eurasian ones (Table 1 in Edwards *et al*. 2012; Yang and Liu 2012). Studies of the effects of hawthorn preparations in treating hyperlipidaemia and cardiovascular conditions have been the subject of meta-analyses that suggest there are significant health benefits to be obtained from these preparations (Guo *et al*. 2008). To date, however, virtually all published studies of the therapeutic components and the therapeutic effects of hawthorn preparations have used Eurasian *Crataegus* species (Yang and Liu 2012). Similarly, hawthorn NHPs sold in Europe and North America predominantly employ raw materials from only a limited number of Eurasian species.

In the southern interior of British Columbia, hawthorns are particularly abundant as a consequence of their weediness, and of agricultural activity and land-use changes (Phipps and O'Kennon 2002; Dickinson *et al*. 2008). The diversity of described hawthorn species is higher here than anywhere else in western North America, comprising widespread species like red-fruited *C. chrysocarpa* (*C.* sect. *Coccineae*) and *C. macracantha* (*C.* sect. *Macracanthae*), as well as black-fruited ones (*C. douglasii*, *C. suksdorfii* in *C.* sect. *Douglasia*) and their hybrids in *Crataegus* series *Douglasianae* and *Purpureofructus* (Zarrei *et al*. 2014) and the naturalized Eurasian red-fruited hawthorn, *C. monogyna*. Native hawthorn species are common throughout the southern half of British Columbia (south of 56° North latitude). In addition to using their wood for hammers, tool handles and digging sticks (Turner 2014*b*), coastal and interior First Nations in British Columbia used the fruits of native hawthorns for food, often smashed so as to break up the pyrenes (and the seeds inside; Turner 2014*b*). First Nation names for both black- and red-fruited hawthorns are documented for the southern half of British Columbia and the adjacent USA, the latter species (*C. chrysocarpa*) mainly in the southern and interior portion of this range (Turner 2014*a*, *b*). In fact, hawthorn species across North America are named by First Nations people, and ethnobotanical uses of hawthorn species are documented for food and for treatments of various conditions, including gastrointestinal, dermatological and heart ailments (Arnason *et al*. 1981; Edwards *et al*. 2012).

Together with widespread use of hawthorn NHPs that are manufactured almost exclusively from a small number of Eurasian species, the diversity and abundance of hawthorns in British Columbia has led to recognition of a potential economic opportunity for locally sourced NHPs. For example, the Naturally Grown Herb and Spice Producers Cooperative (HerbPro; http://herbpronaturals.ca/HerbPro/) has established an agroforestry demonstration farm with hawthorn as the main orchard alley cropping tree. HerbPro has developed a fresh/frozen puree of *C. chrysocarpa* fruit and *C. monogyna* leaf and has had farm-gate sales since 2008. Anecdotal evidence has shown that the puree has positive health benefits for those with cardiovascular disease and hypertension. The raw ingredients for NHPs manufactured and marketed in Canada must be identified and the species listed on product labelling. Collaboration between HerbPro and the academic authors has focussed on evaluating the chemistry and taxonomy of western North American hawthorns, developing identification tools that can be used in the field, and investigating the extent to which DNA barcodes can be used to confirm identifications and authenticate raw ingredients in post-harvest processing (e.g. dried and/or powdered forms of fruit, leaf and flower). Our collaboration also includes limited trials of hawthorn preparations in an animal model of human metabolic syndrome (F. Borthwick *et al.*, unpubl. data—presented in part as Dickinson *et al*. 2014).

Hawthorn preparations, as employed in the animal trials described above and as used in NHP formulations, comprise not only biopolymers (nucleic acids, proteins) but also complex mixtures of much lower molecular weight secondary metabolites. Characterization of these secondary metabolites (the metabolome) by means of analytical instrumentation such as mass spectrometer or nuclear magnetic resonance (NMR) spectrometer (metabolomics; Oliver *et al*. 1998) provides another means by which to describe hawthorn species. Since the therapeutic effects of hawthorn preparations appear to be mediated by the metabolome (notably, phenolic compounds; Edwards *et al*. 2012; Yang and Liu 2012), it is valid to ask to what extent the variation in the metabolomes of *Crataegus* species parallels the variation in DNA sequences. Given that the DNA sequence variation reflects genealogical relationships (we infer the latter from the former), we wish to see whether or not metabolomics resemblances reflect phylogenetic relationships.

To the extent that the molecular data are sufficient (sequence variation in ITS and two chloroplast loci) in a study of *Stachys* (Lamiaceae; Salmaki *et al*. 2013), we know that phytochemical variation (Tundis *et al*. 2014) is not necessarily congruent with phylogeny. Non-metric multidimensional scaling of data for several classes of secondary metabolites (mono-, sesqui- and diterpenes, iridoids, phenylethanoid glycosides, flavonoids) contrasts species of *Stachys* and those of *Sideritis*, while species of the genus *Betonica* are located in a subset of the chemospace occupied by the *Stachys* species (Tundis *et al*. 2014). In the nuclear and plastid phylogenies, however, *Betonica* is one of the outgroups for *Stachys*, and the species of *Sideritis* are nested among those of *Stachys* (Salmaki *et al*. 2013).

Here we examine the utility of the proposed plant DNA barcode loci, comparing the information they provide for hawthorns with that obtained using additional loci and molecular phylogenetic methods. In doing so, we seek to (i) sample the species diversity of hawthorns extensively in order to generate a vouchered library of DNA sequences deposited in BOLD (see **Supporting Information—Table S1**; dx.doi.org/10.5883/DS-NAMCRAT; Ratnasingham and Hebert 2007) and GenBank (Benson *et al*. 2011) from *Crataegus* specimens that have already been identified by expert taxonomists; (ii) assess the utility of the universal plant DNA barcoding markers in species identification, particularly in discrimination of medicinally important taxa; (iii) compare the preceding results with those of phylogenetic analyses of the cpDNA barcoding loci augmented by 11 additional cpDNA loci chosen for the variability they exhibit in *Crataegus* and other Maleae (Lo and Donoghue 2012) and (iv) examine whether polyploidization, apomixis and possibly incomplete concerted evolution of ITS limit the usefulness of DNA barcoding in plant groups where these factors may be frequent (Hollingsworth *et al*. 2011; Zarrei *et al*. 2012, 2014). (v) We provide a preliminary NMR metabolomics dataset with which we examine the relationship between similarities in the therapeutically significant phenolic composition of leaf tissue with that of phylogenetic relationships seen in the cpDNA sequence data.

## Methods

### Plant materials

We sampled DNA barcode loci for 355 *Crataegus* specimens in the Royal Ontario Museum Green Plant Herbarium (TRT; **Supporting Information—Table S1**; BOLD doi:10.5883/DS-NAMCRAT), selected as follows. A total of 275 samples of primarily North American species came from hawthorn trees in Ontario, the southeastern USA, Colorado, Utah, Wyoming and the Pacific Northwest for which we had leaf tissue dried on silica gel, as well as voucher specimens (Talent and Dickinson 2005; Lo *et al*. 2007, 2009; Dickinson *et al*. 2008; Coughlan 2012; Zarrei *et al*. 2012, 2014; Coughlan *et al*. 2014). For these individuals up to 10 accessions per species (or cytotype) were sampled **[see Supporting Information—Table S1]**. This material also included vouchered samples collected at the arboreta and botanical gardens acknowledged in our earlier papers, together with samples of Eurasian species provided to us by K. I. Christensen, A. A. Dönmez and T. Romankova **[see** **Supporting Information—Table S1****]**. A further 80 TRT herbarium specimens of North American species from the J.B. Phipps Hawthorn Research Collection **[see** **Supporting Information—Table S1****]** were sampled as part of the Canadian Centre for DNA Barcoding project, ‘DNA Barcode Flora of Canada’. Of the samples, 38 **[****Supporting Information—Table S1]**, together with samples from 6 additional individuals, were used for more intensive study of cpDNA sequence variation in mainly Pacific Northwest *Crataegus* **[Supporting Information—Table S2]** (cf. Zarrei *et al*. 2014). Finally, a further eight accessions were part of samples used to explore the utility of the two low-copy nuclear markers described below (AT1 and PEPC; **Supporting Information—Tables S3 and S4**). DNA for some of these individuals was obtained from seeds. These 369 *Crataegus* samples represent all of the clades found by Lo *et al*. (2007, 2009), as well as all four sections native to North America and four of six sections native to Eurasia (i.e. not including any of the four species in sections *Cuneatae* and *Henryanae*). Our sample also comprises four named nothosections, as well as 35 series, two named nothoseries and 83 species (plus five varieties, one hybrid and four named nothospecies; **Supporting Information—Tables S1 and S2**). Although just over one-third of our 369 accessions belong to *C.* section *Douglasia*, the taxonomic breadth of our sample is sufficient to let us relate the infrageneric classification **[****Supporting Information—Table S1]** to the phylogenetic structure found by Lo *et al*. (2007) in a similarly wide sampling of the genus. Most of the samples studied come from individuals whose ploidy level and breeding system have been documented either by means of chromosome counts and embryological studies (Dickinson *et al*. 1996), or by means of flow cytometry of leaf and seed tissues (Talent and Dickinson 2005, 2007*a*; **Supporting Information—Tables S1 and S2**).

Based on the results of Campbell *et al*. (2007), Potter *et al*. (2007) and Lo *et al*. (2007), sequences from *Amelanchier*, *Cotoneaster*, *Malus*, *Pyrus* and *Sorbus* were chosen for outgroup rooting of all but one of the trees produced in this study **[see** **Supporting Information—Tables S1–S4****]**. The tree built from sequences of the plastid barcode loci **[see Supporting Information—Fig. S1]** was rooted using the sequences from *Crataegus brachyacantha*, following the results of Lo *et al*. (2007), and based on the similarity of the submarginal venation seen in leaves of this species (unique in *Crataegus*; Fig. 1 in Dickinson *et al*. 2008) to that of *Hesperomeles* (Kelly 2008), a genus shown by Li *et al*. (2012) to be sister to *Crataegus*.

### Molecular methods

Four DNA barcodes (*rbcL*, *mat*K, *psb*A*-trn*H and ITS2; Chase *et al*. 2007; CBOL Plant Working Group 2009; Hollingsworth *et al*. 2011) were generated directly from genomic DNA for the 355 *Crataegus* accessions in the NAMCRAT dataset. DNA was extracted and amplified from leaf tissue using Canadian Centre for DNA Barcoding (CCDB) protocols (Ivanova *et al*. 2011; Kuzmina and Ivanova 2011*a*, *b*). With ITS2, additional primers (White *et al*. 1990; Stanford *et al*. 2000; Chen *et al*. 2010) were needed for successful amplification in some cases. The barcoding sample overlapped partially with that for which additional plastid loci were sequenced (below), and for which cloned ITS2 sequences were studied (Zarrei *et al*. 2014). The successfully amplified amplicons were then sequenced on the 3730xl DNA Analyser (Applied Biosystems) with both forward and reverse primers to assure accuracy.

To increase the discriminatory power of the plastid markers especially in view of the potential consequences of long generation times associated with woodiness in *Crataegus* (Smith and Donoghue 2008), an additional 11 plastid markers (Lo and Donoghue 2012) were sequenced for a subsample of individuals **[see** **Supporting Information—Table S2****]**, using the DNA extraction protocols described in Zarrei *et al*. (2014). They are as follows: *trn*G-*trn*S (Hamilton 1999), *rpl*2-*trn*H (Vaillancourt and Jackson 2000), *rpl*20-*rps*12 (Hamilton 1999), *trn*L-*trn*F (Taberlet *et al*. 1991), *atp*B-*rbcL* (Campbell *et al*. 2007), *rps*16 intron (Campbell *et al*. 2007), *rpl*16 intron (Campbell *et al*. 2007), *trn*C-*yfc*6, *acc*D (forward, 5′-AGAATGGGTACCTCGA-3′; reverse, 5′-GTGTGGTGATCAAGTAGTTA-3′, designed here), *rpo*C1 (Burgess *et al*. 2011) and *atp*F-*atp*H (Burgess *et al*. 2011). For this component of the project, the plastid barcode loci were amplified and sequenced using the following primers: *mat*K (forward, 5′-ACCCCATTCATCTGGAAATCTTGGTTC-3′; reverse, 5′-CGTACAGTACTTTTGTGTTTACGAG-3′, designed here), *rbcL*a (forward, Levin *et al*. 2003; reverse, Kress and Erickson 2007) and *psb*A*-trn*H (forward, Tate and Simpson 2003; reverse, Sang *et al*. 1997). The plastid amplicons were directly sequenced using the PCR primers. Post-PCR steps for all markers were followed as in Zarrei *et al*. (2014). Cycle sequencing reactions were performed using the BigDye^®^ Terminator v3.1 kit (Applied Biosystems, Inc., Foster City, CA, USA). Cleaned cycle sequencing products were sequenced on an ABI 3730 (Applied Biosystems) DNA Analyser at the Royal Ontario Museum (Toronto, Canada). Sequences were proofed and edited using Geneious Pro. v.5.6 (Drummond *et al*. 2012) and assembled using Geneious Pro. v.5.6 or BioEdit v.7.0.5.3 (Hall 1999).

We also investigated two low-copy nuclear markers, i.e. the partial Phosphoenolpyruvate Carboxylase (PEPC) gene and the Pentatricopeptide region (PPR) homologue to the AT1G09680 gene in Arabidopsis (AT1), in smaller samples representing mainly *Crataegus* section *Douglasia* **[see Supporting Information—Tables S3 and S4]**. Details of the methods used are given in **Supporting Information—****File S1**.

### Chemistry sample preparation

Leaves from 14 hawthorn samples representing four species (Table 1) were lyophilized for 24 h and then ground to a fine powder using a food mill. The milled plant material was passed through a 520 µm sieve (30 mesh) to remove any insufficiently milled particles. Two hundred and fifty milligrams of filtered material were extracted sequentially with three aliquots of deuterated methanol (methanol-d4) containing 5 mM 4,4-dimethyl-4-silapentane-1-sulfonic acid (DSS; 1.0, 0.5, 0.5 mL), centrifuged, and the supernatants collected and combined. This extract was filtered through a 0.45 µm polyfluorotetraethylene syringe filter prior to transfer into an NMR tube for analysis.

**Table 1.** Sources of the *Crataegus* leaf samples lyophilized and extracted for the metabolomics assays (Fig. 2). All WKHGC samples were collected from hawthorn individuals grown from bare root stock planted in 2005, 2006 and 2007 as orchard alley cropping trees at the Naturally Grown Herb and Spice Producers Cooperative (HerbPro) agroforestry demonstration farm in Edgewood BC. Ploidy levels in parentheses are those known for the taxon in question; all four *C. suksdorfii* shown to be diploid by flow cytometry.

Taxon	Sample	Locality	Voucher
*C. monogyna* Jacq. (2*x*)			
	WKHGC491	Edgewood BC (49.805, −118.156)	
	WKHGC493	Edgewood BC (49.805, −118.156)	
	WKHGC620	Edgewood BC (49.805, −118.156)	
*C. okanaganensis* J.B. Phipps and O'Kennon (4*x*)			
	WKHGC490	Edgewood BC (49.805, −118.156)	
	WKHGC495	Edgewood BC (49.805, −118.156)	
	WKHGC619	Edgewood BC (49.805, −118.156)	
*C. douglasii* Lindl. (4*x*)			
	WKHGC492	Edgewood BC (49.805, −118.156)	
	WKHGC617	Edgewood BC (49.805, −118.156)	
	WKHGC488	Edgewood BC (49.805, −118.156)	
	WKHGC494	Edgewood BC (49.805, −118.156)	
*C. suksdorfii* (Sarg.) Kruschke—2*x*			
	JC042	OR, Jackson Co. (42.4307, −123.094)	TRT00020321
	JC060	OR, Linn Co. (44.5626, −123.152)	TRT00020146
	JC139	OR, Columbia Co. (46.1106, −122.984)	TRT00020243
	2013-01	OR, Josephine Co. (42.278, −123.647) Deer Creek Center	TRT00028421

### Nuclear magnetic resonance spectra acquisition

A Varian MercuryPlus 400 MHz NMR instrument was used to acquire the metabolomic data (Varian, Inc., Palo Alto, CA, USA). One-dimensional proton spectra NMR experiments were acquired at 25 °C over a spectral width of 4201.7 Hz with an observe pulse of 11.80 µs (90°) using a PRESAT water suppression sequence including a PURGE cycle. The acquisition time was 3.899 s, resulting in a dataset of 16 000 points. A total of 128 transients were acquired for each spectrum. Spectra were processed using MestReNova version 9.0.1 (Mestrelab Research S.L., Santiago de Compostela, Spain) and were manually phased and baseline corrected using a Whittaker Smoother base point detection with spline fitting. The spectra were then referenced, binned to chemical shift widths of 0.005 ppm and normalized to the DSS reference peak. A transposed data matrix of chemical shifts and the intensity values of the superimposed spectra was exported as a comma separated value file (*.CSV) and imported to Microsoft Excel for formatting prior to import into multivariate statistical analysis software. Statistical analysis was performed on the region of the spectrum where the majority of the signals are due to plant phenolic compounds (6.0–8.0 ppm), resulting in a data matrix of 44 samples with 400 observables (intensities in each chemical shift bin). The data were then imported into the multivariate statistical analysis software, Solo version 7.3.1 (Eigenvector Research, Inc., Wenatchee, WA, USA).

### Data analyses

#### Barcoding markers

The DNA sequences were edited and uploaded into the BOLD system (Ratnasingham and Hebert 2007). Following the guidelines proposed by the CBOL Plant Working Group (2009) and Hollingsworth *et al*. (2011), the suitability of DNA barcoding for hawthorns was assessed against three main criteria: (i) universality and marker amplification success, (ii) sequence quality and coverage and (iii) discriminatory power among species. The data were analysed in two steps. Each marker was first analysed individually. Because plastid markers in Maleae appear to be inherited maternally (Corriveau and Coleman 1988; Ishikawa *et al*. 1992; Raspé 2001) and because they are found on a single chromosome as a linkage group (Doyle 1992), all three plastid markers were concatenated into a single matrix. Due to the paralogy issues associated with the ITS2 marker in *Crataegus* (discussed in Zarrei *et al*. 2014), the ITS2 data were not combined with those from the plastid markers in the final analyses.

To assess the universality of markers, we report the per cent sequencing success for each locus (Table 2). The assessment of the sequence quality was performed via the CCBC automated informatics pipeline following the guidelines of the CBOL (2009). Character-based analysis [maximum parsimony (MP) and Bayesian inference] has also proved to be a useful tool for species identification (Lowenstein *et al*. 2009; Kelly *et al*. 2010). However, since Hebert *et al*. (2003*a*, *b*) proposed the use of genetic distance as a standard method for analyses of barcode data, the majority of barcoding studies have followed this distance-based approach (reviewed in Taylor and Harris 2012). Neighbour-Joining (NJ) trees (Saitou and Nei 1987) were built using both the tools made available by BOLD (Jalview 2, Waterhouse *et al*. 2009; Kimura 2 parameter, after alignment by MUSCLE, Edgar 2004) and PAUP* v. 4b10 (Swofford 2003).

**Table 2.** Barcoding information for the four markers investigated here for 355 samples representing 93 *Crataegus* taxa **[see Supporting Information—Table S1]**. ^1^Percentage of individuals successfully sequenced. ^2^Outgroups excluded; calculated using MEGA v. 6.0 (Tamura *et al*. 2013).

Marker	*mat*K	*rbcL*a	*psb*A-*trn*H	ITS2
Aligned sequence length (bp)	783	552	429	671
Unaligned length (mean); excluding end gaps	321 (705.7 ± 99.3) 783	485 (551.3 ± 5.3) 552	183 (274.9 ± 22.0) 429	112 (312 ± 92.5) 587
Pairwise % identity	99.3	99.8	87.6	79.8
Number of taxa successfully amplified and sequences	82	93	81	65
Number of samples successfully amplified and sequenced	255	340	290	192
% sequencing success^1^	71.8	95.8	81.7	54.1
Overall mean sequence divergence^2^	0.001	0.002	0.128	0.135

Our evaluation of the success of the different barcode loci corresponds to the tree-based methods described by Ross *et al*. (2008). These authors used simulations to conclude that other approaches to using genetic distance data to evaluate barcoding success (BLAST, comparisons of the distances between unknowns and candidate reference taxa) will fail when not all candidate species are represented unambiguously in the set of reference taxa. Thus we know that because of the relatively limited sequence variation seen within the Maleae with individual chloroplast loci (Evans *et al*. 2000; Campbell *et al*. 2007; Potter *et al*. 2007) the correspondence between a barcode sequence and a taxon may be ambiguous. The abundance of ITS2 paralogs found by Zarrei *et al*. (2014) will have the same effect.

#### Additional plastid markers

All plastid sequences were submitted to GenBank (Benson *et al*. 2011), and the accession numbers are available in **Supporting Information—Table S2**. The sequences were aligned with CLUSTAL X (Thompson *et al*. 1997) or Geneious alignment option of Geneious Pro v.5.6 (Drummond *et al*. 2012). This initial alignment was adjusted manually in BioEdit v.7.0.5.3 or Geneious Pro v.5.6 to minimize steps in the most parsimonious trees (e.g. Koch *et al*. 2010). Because all 14 plastid markers (including the three barcode markers) used in this study are linked, the concatenated sequence matrix was analysed. However, separate parsimony analyses were conducted for each marker to investigate the possible incongruence (discussed in Bull *et al*. 1993) among datasets. Two different phylogenetic analyses were run for the concatenated regions: (i) MP analyses using PAUP* v. 4b10 (Swofford 2003) and (ii) Bayesian analyses (BIs; Yang and Rannala 1997) using MrBayes v. 3.2.0 (Ronquist and Huelsenbeck 2003; Ronquist *et al*. 2010). The details of each analysis are the same as in Zarrei *et al*. (2014). Indels were coded as separate presence/absence characters using SeqState version 1.4.1 (Müller 2005) with modified complex coding option originally described by Simmons and Ochoterena (2000) and appended to the end of matrices.

In the parsimony analysis, the character state changes were equally weighted and character changes were interpreted under ACCTRAN optimization (Agnarsson and Miller 2008). A two-stage strategy of Fitch parsimony (Fitch 1971) search was undertaken following Zarrei *et al*. (2014). The phylogenetic reliability was assessed using non-parametric bootstrapping. The bootstrap support (BS) was estimated using 1000 bootstrap pseudoreplicates with simple taxon addition and TBR swapping but permitting only 10 trees per replicate to be held. The consistency index (CI), rescaled consistency index (RC) and Farris's (1989) retention index (RI) were calculated to measure the amount of homoplasy in the dataset. The best-fit model for each region in the plastid concatenated matrix is provided in **Supporting Information—Table S5**. These models were selected by Akaike information criterion (AIC; Akaike 1974), as implemented in MrModeltest v. 2.3 (Nylander 2004).

For the BIs, two simultaneous runs with four chains each were run for 20 million generations. In each run, every 2000th tree was sampled. The completion of the BI was determined when the average standard deviation of split frequencies ≤0.05 (Ronquist and Huelsenbeck 2003) for the combined two runs was assumed and the complete convergence between the Bayesian Markov chain Monte Carlo runs was reached. Convergence of an independent search was further explored by plotting likelihood scores vs generations using the program Tracer v1.5 (Rambaut and Drummond 2007). The burn-in phase for each run—the first 25 % of sampled trees—was discarded during computing the phylogram consensus tree based on the average branch lengths (50 % majority rule) of the remaining trees (15 000 trees) using *sumt* command implemented in MrBayes. Support for Bayesian topologies was estimated using node posterior probabilities (PPs) from the posterior distribution of topologies.

#### Metabolomic data

Classes were defined for each sample according to its species identification. Before multivariate analysis, mean centring and Pareto scaling, where each variable is divided by the square of its standard deviation, were applied to the dataset (van den Berg *et al*. 2006). Following this, a hierarchical cluster analysis (HCA) was performed. The HCA dendrogram was generated using Ward's minimum variance method with Mahalanobis distance and generalized least squares weighting to *α* = 0.001.

## Results

### Barcoding markers

Across all four regions investigated, a total of 1077 assembled DNA sequences were obtained from 355 samples (mean = 3.03 regions sequenced per sample; BOLD dx.doi.org/10.5883/DS-NAMCRAT, Table 2; **Supporting Information—Table S1**). The overall average sample sequencing success was 75.8 %, ranging from 54.1 % for ITS2 to 95.8 % for *rbcL*a (Table 2). Internal transcribed spacer 2 had the lowest species sequencing success (69.9 %), compared with 100 % for *rbcL*a (Table 2). The sequences were retrieved for all the markers using both the forward and reverse primers. The variation in both the sequence length (because of insertions or deletions) and additive polymorphic sites (APS) was detected in the sequences of ITS2 due to the presence of multiple copies of ITS2 in the genome of each individual (see Discussion for more detail). No APS were detected in the traces obtained from the plastid markers. However, displacement of electropherograms was observed in *psb*A*-trn*H tracers due to the presence of homopolymer runs (Devey *et al*. 2009; Fazekas *et al*. 2010). Sequencing with the reverse primer resolved this problem. Pairwise percentage sequence identity varied from a low of 79.8 % (ITS2; estimated using Geneious Pro. v.5.6, Drummond *et al*. 2012; Table 2), to a high of 99.8 % (*rbcL*a; Table 2). The lowest sequence identity among the plastid barcode markers investigated here was 87.6 % for the *psb*A*-trn*H spacer (Table 2).

#### Resolution of the barcode loci

Despite earlier success using barcode sequence data to confirm the parentage of two hybrids (Christensen *et al*. 2014), only a very limited number of *Crataegus* species are diagnosable using individual plastid barcode loci (Table 3; cf. Fineschi *et al*. 2005). In addition to the six taxa (five species and one hybrid) for which at least one locus provided a diagnostic position (Table 3), single-nucleotide polymorphisms also diagnose two interesting groupings including the autotriploid *C. gaylussacia* with its probable progenitor, diploid *C. suksdorfii* (*mat*K, Table 3; Zarrei *et al*. 2014). The other grouping of interest is that of *C.* × *canescens* together with (i) *C.* sect. *Crataegus* and its hybrids with *C. punctata* (Christensen *et al*. 2014), (ii) *C. brachyacantha*, and *C. spathulata* (*rbcL*a, Table 3) and (iii) *C. germanica* (*C.* sect. *Mespilus*), and all of the *C.* ser. *Cerrones* taxa in the sample (*psb*A*-trn*H, Table 3). On the basis of their molecular phylogeny of *Crataegus*, Lo *et al*. (2007) suggested that *C.* × *canescens* (originally described as *Mespilus canescens* J.B. Phipps) was likely a hybrid involving *C. germanica*, *C. brachyacantha* and an unknown third taxon.

**Table 3.** Single-nucleotide changes in the three chloroplast markers investigated in *Crataegus* for 355 samples representing 93 distinct taxa **[see Supporting Information—Table S1]**. With these loci the accessions belonging to six taxa formed distinct single-taxon clusters in the corresponding NJ tree calculated by BOLD; these clusters result from the polymorphisms shown (position in the consensus sequence). In bold, polymorphisms and positions scored as diagnostic by BOLD (*n* ≥ 3). Ploidy levels shown are based on data from earlier studies (Talent and Dickinson 2005; Coughlan *et al*. 2014; Zarrei *et al*. 2014). In addition to *C.* × *canescens* (Lo *et al*. 2007), *C. spathulata* (Lo *et al*. 2007) and *C. nigra* (Zarrei *et al*. 2014) are suspected of being (paleo-) hybrids.

Marker	*mat*K	*rbcL*a	*psb*A-*trn*H
*C.* × *canescens* (allotriploid; *n* = 6)		T → C (5) with *N* = 21 diploid *C.* sect. *Crataegus* and 4 *C.* × *ninae-celottiae*	G → C (287) with *N* = 10 diploid *C. germanica* and 17 *C.* ser. *Cerrones*
		A→G (57)	
		**C → G (121)**	
		A→G (391)	
*C. brachyacantha* (diploid; *n* = 9)		A→G (57)	
		**G → C (244)**	
		**G → A (357)**	
		A →G (391)	
*C. spathulata* (diploid; *n* = 3)	**T → A (238)**	A→G (57)	G→A (91)
	**A → G (621)**	A→G (391)	
*C. gaylussacia* (autotriploid; *n* = 6)	C→T (288) with *N* = 7 diploid *C. suksdorfii*	**C → T (417)**	
*C. nigra* (diploid; *n* = 3)	G→T (277)	**A → C (120)**	
*C. pinnatifida* (diploids, triploids, tetraploids and hexaploids known; *n* = 3)	T→G (75) with *N*=3 of seven *C. macracantha*		G→T (197)
	**G → A (354)** with *N* = 2 *C. hupehensis*		

Combining the sequences of the three plastid barcode loci into a single alignment yielded an NJ tree **[see Supporting Information—Fig. S1]** in which some more and better-resolved clusters appeared, but few of these comprised only a single species, or even only closely related species (e.g. belonging to the same taxonomic series **[see Supporting Information—Fig. S1]**). Also, topological relationships between groups expected from earlier analyses of more informative loci (Lo *et al*. 2007, 2009; Zarrei *et al*. 2014) were not recovered in the NJ tree based only on *rbcL*a, *mat*K and *psb*A*-trn*H data **[see Supporting Information—Fig. S1]**.

The NJ tree based on ITS2 sequences obtained by direct amplification of genomic DNA according to the BOLD protocol **[see Supporting Information—Fig. S2]** is similarly problematic. As described earlier (Dickinson *et al*. 2011), while some diploids formed well-resolved single-species clusters (**Supporting Information—Fig. S2**; e.g. *C. brachyacantha*, *C. germanica*, *C. spathulata*), others did not (**Supporting Information—Fig. S2**; e.g. *C. marshallii*, *C. punctata*). Polyploids were found in heterogeneous clusters that in some cases did not include the most closely related diploid species (**Supporting Information—Fig. S2**; e.g. *C*. ser. *Cerrones*).

### Phylogenetic analysis of 14 plastid markers

**Figure 1.**
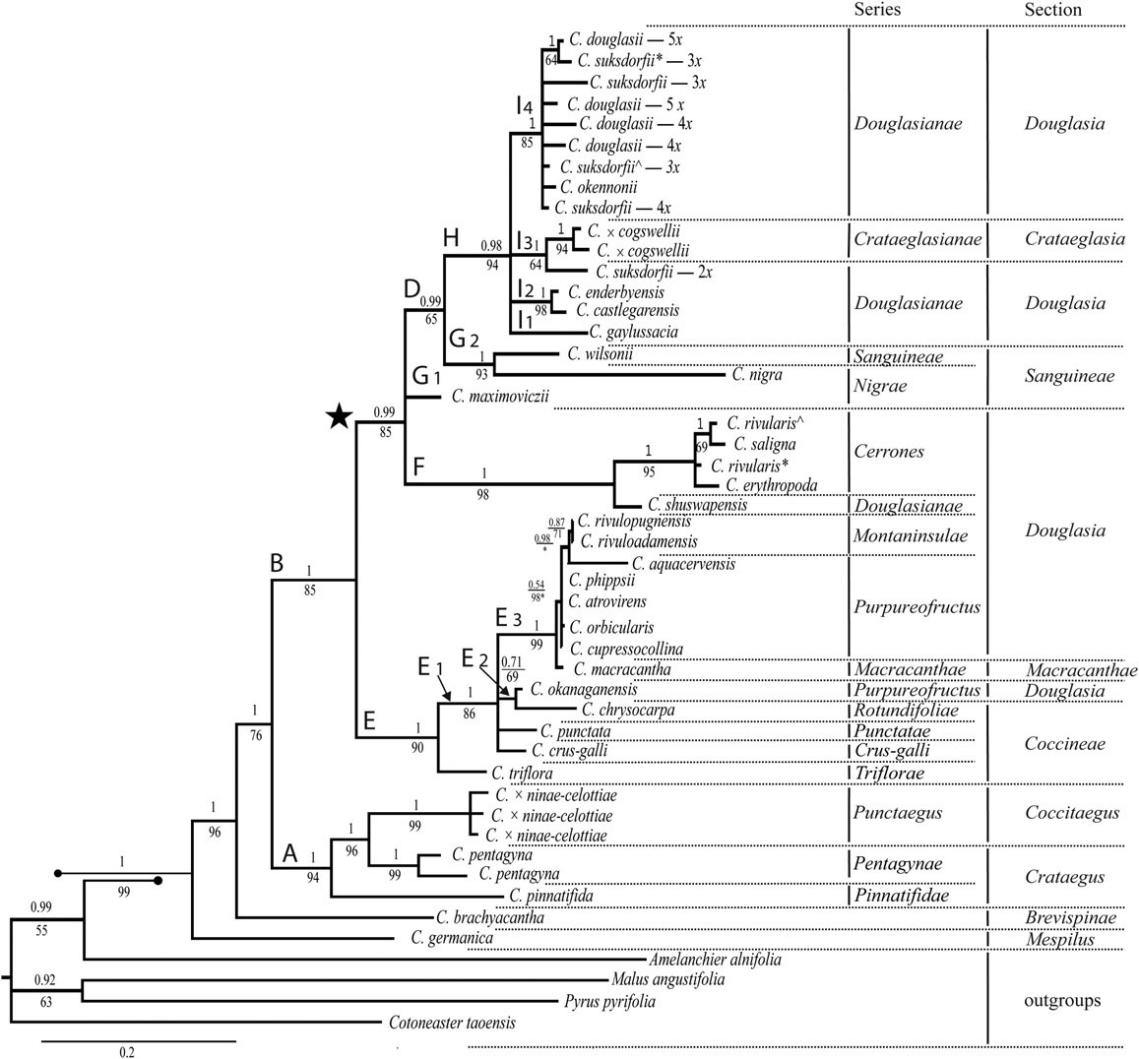
*Crataegus* phylogram based on sequence data from 14 plastid loci **[see Supporting Information—Table S2]**; majority rule consensus of 15 000 Bayesian trees. Numbers above branches are PP; numbers below the branches are percent BS. For ease of comparison, clades are labelled as in Fig. 4 of Zarrei *et al*. (2014). Scale for branch lengths is substitutions per site. *Crataegus suksdorfii* 3x* = sampled from Haida Gwaii; *C. suksdorfii* 3*x*^ = sampled from Vancouver Island; C. rivularis* sampled from Colorado (NT273); *C. rivularis*^ sampled from Wyoming (2001–42). An asterisk following the bootstrap value indicates the branches collapse in the strict consensus tree of 18 trees. The infrageneric classification mapped onto the tree is based on Christensen *et al*. (2014), Dickinson *et al*. (in prep.) and Phipps (2015).

No incongruence was detected between datasets in separate parsimony analyses of the 14 markers. In contrast to clustering based on one or a few barcode loci, phylogenetic analysis of a total of 560 sequences for 14 markers **[see Supporting Information—Table S6]** yielded much greater resolution, and recovered the groups and topological relationships expected from earlier analyses (in Fig. 1, branches are labelled so as to correspond as nearly as possible with those in Fig. 4 of Zarrei *et al*. 2014). The concatenated plastid matrix comprised 10 570 sites; of which, 487 (4.6 %) were variable and 190 (1.84 %) were parsimony-informative (including outgroups). The loci with highest percentages of parsimony-informative sites were *rpl*2-*trn*H (3.65 %) and *psb*A*-trn*H (3.35 %). Two markers, *rpo*C1 (0.18 %) and *rbcL*a (0.36 %), yielded the fewest parsimony-informative sites. Due to the very low resolution of the trees resulting from the individual markers, these trees are not shown. The first and second stage of parsimony analysis (Zarrei *et al*. 2014) of the concatenated plastid matrix generated 18 trees, each with: 587 steps, a CI of 0.86, a RC of 0.77 and a RI of 0.89 (Fig. 1). The ingroup is well supported (PP = 1; BS = 99).

Topological relationships between groups expected from earlier analyses (Lo *et al*. 2007, 2009; Zarrei *et al*. 2014) were found here (Fig. 1), as was not the case in the analysis of only three concatenated plastid loci **[see Supporting Information—Fig. S1]** or in that of the ITS2 sequences amplified from genomic DNA **[see Supporting Information—Fig. S2]**. *Crataegus germanica* (*C.* section *Mespilus*) and *C. brachyacantha* (*C.* section *Brevispinae*) branch as successive sisters to the remaining ingroup taxa. *Crataegus* section *Crataegus* forms a well-supported group (Fig. 1, clade A; PP = 1, BS = 94). Its sister clade (Fig. 1, Clade B) comprises *C.* sections *Coccineae* and *Macracanthae* mainly of eastern North America (Fig. 1, clade E) and an unlabelled clade (star, Fig. 1). This unlabelled clade consists of the remaining Eurasian species (in *C.* sect. *Sanguineae*, branches G_1_ and G_2_) and the primarily western North American *C.* sect. *Douglasia* (Fig. 1, clades F and H).

Clade D comprises species belonging to *C.* section *Sanguineae* and *C.* section *Douglasia* (Fig. 1). The phylogenetic relationship of *C. maximoviczii* (*C.* section *Sanguineae*; Fig. 1, clade G_1_) is not resolved and this species is in a polytomy with clades D and F. Clade F comprises *C.* series *Cerrones* (PP = 1; BS = 95) from the central Rocky Mountains, and the southern British Columbia allotetraploid, *C. shuswapensis* (*C.* series *Douglasianae*).

As noted earlier (Zarrei *et al*. 2014), the behaviour of well-documented recent intersectional hybrids (Christensen *et al*. 2014) helps elucidate the allopolyploid status of a number of species in *C.* section *Douglasia*. Three individuals of *C.* × *ninae-celottiae* (=*C. monogyna* × *C. punctata*; Christensen *et al*. 2014) analysed here are grouped with other members of sect. *Crataegus* in clade A with PP = 1 and BS = 99 (Fig. 1). In contrast, the two individuals of *C.* × *cogswellii* (=diploid *C. suksdorfii*× *C. monogyna*; Christensen *et al*. 2014) in our sample form a clade (*I*_3_, Fig. 1) with diploid *C. suksdorfii* with PP = 1 and BS = 98. Both nothospecies arose from hybridization over the past 200 years between native North American diploids and the introduced Eurasian species, *C. monogyna* (Christensen *et al*. 2014). The cladistic relationships between these hybrids and their parents seen in a phylogeny based on maternally inherited loci provide a good indication of the predominant direction in which hybridization has taken place (Christensen *et al*. 2014).

The same logic applies to the cladistic relationships between allopolyploids in *C.* series *Douglasianae* (Fig. 1, clade H, mostly) and those in *C.* nothoseries *Montaninsulae* and *Purpureofructus* (Fig. 1, clades E_2_ and E_3_). These relationships (Fig. 1) suggest that tetraploid *C. chrysocarpa* (or another member of *C.* series *Rotundifoliae*) is the female parent of *C. okanaganensis* (also tetraploid; clade E_2_, PP = 0.71, BS = 69), whereas tetraploid *C. macracantha* (or another member of *C.* sect. *Macracanthae*) is the female parent of the remaining section *Douglasia* species in clade E_3_ (PP = 1, BS = 99). Members of *C.* sect. *Coccineae* (probably also either *C. chrysocarpa* or *C. macracantha*, as these species are the only ones with any appreciable range in the trans-Mississippi west) appear to have been the male parents of the other allopolyploids in *C.* section *Douglasia* (Fig. 1; clades I_2_, *C. castlegarensis* and *C. enderbyensis*, and I_4_, *C. douglasii* and *C. okennoni*; Zarrei *et al*. 2014). As in the case of the two diploid nothospecies derived from *C. monogyna* (Christensen *et al*. 2014), the presence of cloned ITS2 variants from both *C.* section *Coccineae* and *C.* section *Douglasia* in these tetraploid nothospecies clinched their hybrid status (Zarrei *et al*. 2014). Finally, the same logic elucidates the origin of polyploid *C. suksdorfii* (Fig. 1, clade I4; Zarrei *et al*. 2014).

### Metabolomic analysis of Crataegus species

**Figure 2.**
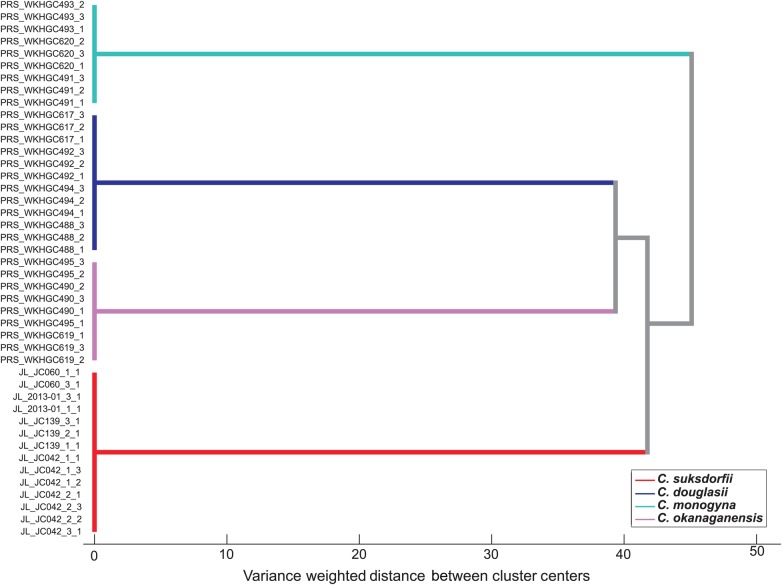
Hierarchical cluster analysis of ^1^H NMR metabolomics data of phenolic compounds (6–8 ppm) from four *Crataegus* species (Table 1).

Comparison of four *Crataegus* species (Fig. 2) with respect to their ^1^H NMR metabolomics data demonstrates the greater similarity of the two allotetraploids in the sample to each other, relative to their putative common ancestor, diploid *C. suksdorfii. Crataegus monogyna* is most dissimilar, and in this respect these limited metabolomics results parallel the phylogenetic relationships determined from both chloroplast DNA sequence data (Fig. 1) and nuclear DNA (Lo *et al*. 2007, 2009; Zarrei *et al*. 2014). This analysis targeted phenolic compounds because they have demonstrated cardioprotective activity. As specialized metabolites they are also more likely to vary more between species than within samples compared with primary metabolites such as sugars.

## Discussion

Using data from 93 mostly species-level *Crataegus* taxa that represent all major clades and 8 out of 10 sections of the genus, we assess DNA barcode markers using the following three criteria: (i) universality and marker amplification success, (ii) sequence quality and coverage and (iii) discriminatory power among species. We will also comment on the additional information that our DNA barcode data provide and on the serious limitations that our results place on using DNA barcodes to identify hawthorn species and authenticate hawthorn NHPs.

### Barcode universality and amplification success in Crataegus

The plastid markers met the first criterion for DNA barcoding, namely that amplification succeeded with almost all hawthorn accessions tested (87.1–100 % of the taxa amplified; Table 2). In contrast, amplification of the ITS2 region was generally less successful, and obtaining the data analysed here required recourse to additional primer pairs (65 taxa amplified; Table 2).

### Crataegus barcode sequence quality and coverage

The plastid markers also met the second criterion for DNA barcoding, namely that sequence quality was generally high for each of the markers investigated here (Table 2). However, the sequencing success for *mat*K was lower than that for the rest of the plastid markers. The ITS2 region had the lowest rate of sequencing success (69.9 %). Sequence quality was also low so that, overall, ITS2 yielded the fewest barcode sequences (Table 2).

### The limits of plastid DNA barcode discrimination of Crataegus species

There are more than 200 *Crataegus* species native to North America (Phipps 2015). We have sampled 93 mostly species-level taxa, and our accessions represent all four native sections, one naturalized section and the four nothosections of the genus that are currently recognized (Lo *et al*. 2007; Brouillet *et al*. 2010+; Christensen *et al*. 2014). In addition, our accessions represent a comprehensive sample of *Crataegus* in western North America, and of *C.* section *Douglasia*, thus matching recommendations in Piredda *et al*. (2011) for ensuring that the correct relationship between barcode sequences and species is obtained. Individually, the nucleotide diversity of the three plastid DNA barcode markers was low and distinguishes only three species and one nothospecies (Table 2; five in all, if the European species *C. nigra* is included). Similar results using just the *psb*A*-trn*H barcode locus have been obtained in *Fragaria*, where only two species out of 21 could be distinguished (Njuguna and Bassil 2011). In the NJ tree for the concatenated plastid loci (317 individuals with data for at least two loci), only one more species forms a distinct single-taxon cluster (**Supporting Information—Fig. S1**, diploid *C. germanica*). We have little doubt that these tallies could be marginally improved with further sampling of species, and of individuals within species, but not to a large extent. Comparison of the plastid and nuclear genomes (Wolfe *et al*. 1987; Clegg *et al*. 1994), as well as studies of the molecular systematics of the Rosaceae in general and the Maleae in particular (Evans *et al*. 2000; Campbell *et al*. 2007; Potter *et al*. 2007; cf. Nikiforova *et al*. 2013), all point to low levels of sequence variation in plastid loci especially in recently evolved species. As a result, in *Crataegus* a reasonable degree of phylogenetic resolution was only obtained by concatenating sequence data from 14 plastid loci (Fig. 1; cf. Lo and Donoghue 2012), but this is not a solution acceptable from the current barcoding point of view.

### The limits of ITS2 DNA barcode discrimination of Crataegus species

The intragenomic variability of the ITS2 region in *Crataegus* has negative consequences for DNA barcoding. Several different copies of ITS2 (ribotypes) are present in the genomes not only of polyploids, but also diploids (Zarrei *et al*. 2014), including recent hybrids (e.g. *C.*× *cogswellii* and *C.*× *ninae-celottiae*; Love and Feigen 1978; Wells and Phipps 1989; Christensen *et al*. 2014). Both indels and APS among ribotypes limit direct amplification of sequences from genomic DNA. For example, high-quality sequences could not be obtained from the recent hybrids *C.*× *cogswellii* and *C.*× *ninae-celottiae*. Several indels at different sites caused electropherogram displacements. Although bidirectionally sequencing this short marker (mean sequence length = 314) contributed to sequence recovery, the problem associated with identifying the paralogs still remained. Cloning the ITS2 amplicons revealed the extent of hybridization in polyploid black-fruited taxa of *Crataegus* by demonstrating the co-occurrence in a single individual of ITS2 sequences from sections *Coccineae* and *Douglasia* (Zarrei *et al*. 2014). This highlights the limited utility of ITS2 as a barcoding marker particularly for allopolyploid taxa (Hollingsworth *et al*. 2011).

The DNA barcoding protocol of direct sequencing of ITS2 is thus likely to lead to incorrect species identifications by randomly amplifying only a single ribotype, especially from a polyploid in which the number of paralogs is unknown. In the case of diploid *C. spathulata*, all three individuals form a single, distinct cluster (clade C_1_, **Supporting Information—Fig. S2**; see also *C. brachyacantha*, clade A_2_, and *C. germanica*, clade A_1_). However, an equally distinct cluster (**Supporting Information—Fig. S2**, clade C_2_) comprises all three individuals of diploid *C. marshallii* (series *Apiifoliae* in section *Crataegus*) plus *C. displar*, a tetraploid in series *Lacrimatae* (section *Coccineae*). Another example is *C. pinnatifida* (section *Crataegus*); ITS2 sequences were obtained for two of the individuals of this species **[see Supporting Information—Table S1]**. One of these (TRT099) forms a distinct cluster with *C. hupehensis* (section *Hupehenses*; **Supporting Information—Fig. S2**), while the other (TRT100) clusters with *C. wilsonii* (section *Sanguineae*; **Supporting Information—Fig. S2**). In fact, few of the clusters found on the NJ tree of the ITS2 sequences **[see Supporting Information—Fig. S2]** contain only a single species, even though these clusters may comprise taxa belonging to the same series. We can offer no explanation for the examples described above of disparate taxa forming small, well-defined clades. On the other hand, the way in which some tetraploid individuals from section *Douglasia* are found interspersed among individuals from section *Coccineae* in this tree **[see Supporting Information—Fig. S2]** is undoubtedly related to the way the *Douglasia* taxa concerned were found by Zarrei *et al*. (2014) to comprise individuals containing both *Coccineae* and *Douglasia* ITS2 sequences.

The problem associated with ITS2 as a barcode marker stems partly from the limited sequence variation that this locus exhibits in *Crataegus* (Zarrei *et al*. 2014), and partly from the incomplete homogenization of this locus by concerted evolution (Arnheim 1983; Zarrei *et al*. 2014). The rate at which molecular forces homogenize ITS2 is evidently lower than that at which hybridization and polyploidization have added ribotypes to an individual's genome (in a context where gametophytic apomixis produces unreduced female gametes which then undergo either parthenogenesis or fertilization; Talent and Dickinson 2005, 2007*a*, *b*). Similar results are seen with low-copy number nuclear genes like AT1 **[see Supporting Information—Fig. S3]** and PEPC (**Supporting Information—Fig. S4** **and File S1**). Neither of these issues has been considered in recent evaluations of ITS2 as a suitable DNA barcode marker in medicinal plants (Chen *et al*. 2010) or in the Rosaceae (Pang *et al*. 2011). In fact, Chen *et al*. (2010) did not sequence *Crataegus* ITS2 themselves, relying instead on sequences deposited in GenBank by E. Y. Y. Lo (Table S5 in Chen *et al*. 2010). Pang *et al*. (2011, their Table S1), only sequenced ITS2 in three varieties of *C. pinnatifida* and relied again almost exclusively on sequences deposited in GenBank by Lo (Table S2 in Pang *et al*. 2011). In both these studies, only the BLAST- and distance-based methods of evaluation of Ross *et al*. (2008) were used. Both these studies implicitly assumed a one-to-one correspondence between species and unique ITS2 sequences. Such a correspondence is necessary for the success of the BLAST- and distance-based methods of evaluation (Ross *et al*. 2008). These methods will break down when there is a one-to-many relationship between species and sequences, as is the case when not all of the possible ITS2 paralogs have been included in the reference dataset, and any given paralog may occur in more than one species. As we have shown elsewhere (Zarrei *et al*. 2014), the ITS sequences obtained in our earlier work (Lo *et al*. 2007, 2009) are likely to underestimate the intragenomic diversity at this locus in many *Crataegus* taxa.

### Metabolomics data

Relationships inferred from molecular data (Fig. 1) are also implied in the resemblances obtained with the small sample studied here for the flavonoid component of the metabolome (Fig. 2). More metabolomics data from a more representative sample are needed, however, before we can tell whether these data track the relationships seen in *Crataegus* molecular data or not, and whether chemical tests will be more useful than DNA barcode loci in discriminating hawthorn species used in NHPs.

### Limited utility of DNA barcode data for studies of Crataegus phylogeny

Sequence data from DNA barcode loci have proved useful in clarifying relationships between recent *Crataegus* hybrids and their parents where the latter were both diploids and from different clades (Christensen *et al*. 2014; cf. Lo *et al*. 2007). For the variety of reasons described above, these data are nevertheless inadequate for either barcoding purposes or for revealing evolutionary relationships across the entire genus **[see Supporting Information—Figs S1 and S2]**. Plastid loci can be chosen much more strategically if appreciable phylogenetic resolution is sought from a limited number of loci (Zarrei *et al*. 2014). Alternatively, alignments of sequences of considerably more than just three plastid loci can be analysed together in order to obtain a useful degree of phylogenetic resolution. When this approach is taken (Fig. 1), the results robustly corroborate earlier work on the same sample (Zarrei *et al*. 2014).

Comparison can also be made between our results and those of Lo and Donoghue (2012; a study of intergeneric relationships in the Maleae) as they relate to *Crataegus*. Their Fig. 1 is a tree based on ITS (cloned only when direct sequencing gave ambiguous nucleotides) plus the same 11 plastid loci studied here in addition to the barcoding loci *rbcL*, *mat*K and *psb*A*-trn*H, for a larger sample of hawthorn species than we have used (7 of the 47 Crataegus accessions, and 20 of the 33 species in **Supporting Information—Table S2** are shared with the Lo and Donoghue study). Even after Lo and Donoghue removed the two genera responsible for a lack of congruence between their ITS and plastid data, their tree based on the combined datasets shows support values that are marginally lower than those obtained here, with just 14 plastid loci, for branches A, B, C and E (Fig. 1). In both their study and ours, it is noteworthy that *C.* series *Cerrones* (clade F, Fig. 1) is sister to clade D, comprising both East Asian *C.* section *Sanguineae* (clades G_1_ and G_2_, Fig. 1) and North American *C.* series *Douglasianae* (clade H), suggesting that the origin of section *Sanguineae* involved an east to west trans-Beringian migration, from western North America into eastern Asia. These results also warrant possibly recognizing the *Cerrones* as a section, that is, at the same taxonomic rank as sections *Douglasia* and *Sanguineae*  (Fig. 1) **[see Supporting Information—Table S1]**.

### Limited utility of DNA barcode data for Crataegus NHP development

Various authors have pointed out that DNA barcoding will not be useful in particular plant groups. Spooner (2009) examined the utility of barcoding in wild potatoes and concluded that plastid loci may lack the necessary polymorphism, while ITS may exhibit too much intraspecific variation, much as we have seen here. Hollingsworth *et al*. (2011) list factors that will work against barcoding success: breeding system, hybridization, polyploidy, long generation times (or reduced mutation rates), narrow taxon concepts, species history and seed dispersal. Like many other genera in the Rosaceae, *Crataegus* is a woody perennial that exhibits a nexus of frequent hybridization, production and fertilization of unreduced gametes, hence polyploidy and accompanying shifts to self compatibility (Dickinson *et al*. 2007; Hojsgaard *et al*. 2014; Zarrei *et al*. 2014). Arguably as a result of the patterns of morphological variation associated with frequent apomixis and selfing (Dickinson and Phipps 1985; Dickinson 1986; cf. Lo *et al*. 2010), the (morphological) species concepts used in *Crataegus* have been quite narrow, and in many cases do not likely reflect a high degree of genetic differentiation. Concerning species history, Hollingsworth *et al*. (2011) refer to the effects of recent, rapid radiation on the one hand, and on the other hand, to the maintenance of genetic polymorphisms in large populations, as also contributing to the failure of DNA barcoding within a group. In the case of *Crataegus*, it seems likely that many hybridization and polyploidization events have been relatively recent, post-Pleistocene (<12 000 years) in any case, and possibly just in the last millennium, as a consequence of First Nation and then European land-clearing activities (Marie-Victorin 1938; Dickinson *et al*. 2008).

In fact, the morphology visible in hawthorn flowering and fruiting voucher specimens is adequate for identification of most western North American species (Dickinson 2012) because, being long-lived woody perennials, wild hawthorns are readily marked and vouchered at both seasons for comparison (Hildreth *et al*. 2007). It is also significant that in this geographic area there are only a limited number of common species, almost all of which have now been included in flow cytometric surveys of ploidy level and breeding system (**Supporting Information—Tables S1 and S2**; Talent and Dickinson 2005; Talent unpubl. data). In contrast, the *Crataegus* flora of eastern North America is much richer, and is less well represented in molecular phylogenetic studies (Lo *et al*. 2009; Zarrei *et al*. 2014). As a consequence, only a few groups can be considered even moderately well-characterized [e.g. series *Aestivales* and *Brevispinae*, and the North American series assigned to section *Crataegus* by Phipps (2015)]. More data are available from flow cytometry, and these have informed regional studies (Lance 2014), but coverage is not yet complete. Fortunately, a comprehensive floristic treatment of North American *Crataegus*, complete with extensive synonymies, is available now as a part of the *Flora North America* project (Phipps 2015). By providing testable taxonomic hypotheses, this treatment should stimulate the collection and analysis of new data with which to make sense of the taxonomic complexity that has been the hallmark of eastern North American hawthorns for over 100 years.

Production of hawthorn NHPs that employ native North American *Crataegus* species as raw materials thus should not be impeded by an inability to employ DNA barcoding to authenticate source taxa. Data on the predominant agamospermy of polyploid *Crataegus* (all western species except for two; Talent and Dickinson 2007*a*, *b*) suggest that orchards raised from seeds or developed by grafting from previously identified and vouchered genotypes can be harvested over multiple years, thus minimizing the need for raw material authentication. Limited trials in an animal model of human metabolic syndrome of hawthorn preparations from native *C. chrysocarpa* fruit and BC-grown *C. monogyna* leaves have shown significant improvements over untreated controls (F. Borthwick *et al.*, unpubl. data—presented in part as Dickinson *et al*. 2014). In addition to information on the taxonomy and phylogeny of hawthorns, information on hawthorn chemistry, analytical methods and the validation of those methods are increasingly available (Kirakosyan *et al*. 2005; Edwards *et al*. 2012). Discovery of metabolomic variation between hawthorn species suggests the possibility that different therapeutic outcomes may be obtained when North American NHPs are administered rather than the more ubiquitous hawthorn NHPs originating from European species.

## Conclusions

We have examined the utility of DNA barcoding in a sample of 355 accessions representing 93 mostly species-level taxa from all major clades known to date, in a moderately large plant genus, *Crataegus*. Our sequence data and voucher information (including specimen images) represent well-studied authoritatively identified individuals and are publicly available on the BOLD website (dx.doi.org/10.5883/DS-NAMCRAT; sequence data also on GenBank, **Supporting Information—Tables S1–S4**) where they can be consulted, downloaded and reanalysed. The preliminary results from the small metabolomics dataset studied here show the promise of NMR chemotaxonomic data for studies of *Crataegus* in relation to variation in therapeutic applications or NHP raw material authentication.

Our analyses of these sequence data from three plastid loci and ITS2 generally failed to recover either the cladistic structure or the morphology-based infrageneric classification of *Crataegus*. We attribute this result to the lack of variation within Rosaceae tribe Maleae in the plastid loci chosen as barcodes. The use of biparentally inherited ITS2 as a DNA barcode is confounded by the frequency of allopolyploidy in *Crataegus* combined with incomplete homogenization of this locus by concerted evolution. Incomplete concerted evolution of this kind leading to the presence of multiple gene copies is also common in the human genome (Zarrei *et al*. 2015).

In *Crataegus*, resolution of species or of groups of closely related species, depends on having more phylogenetically informative sites than can be provided by a small number of plastid loci. Relatively well-resolved phylogenetic analyses required concatenated sequences from a total of 14 plastid loci (Fig. 1). High-resolution phylogenetic analyses based on the nuclear genome will require data from very low-copy number loci, the paralogs of which are readily identifiable. Since phenolic compounds, widely purported to be beneficial in cardiovascular health, vary in composition between species, there is considerable potential for using this chemical information to choose an optimal species for NHP formulation. However, development of hawthorn NHPs using North American species will require a taxonomy bolstered by molecular data that are interpretable in the field using morphological characters. This is now largely available for western North America, but remains a challenge in the much more complex *Crataegus* flora of eastern North America.

## Accession Numbers

See **Supporting Information—Tables S1–S4** for GenBank accession numbers for the DNA sequences studied here.

## Sources of Funding

We gratefully acknowledge the Canada Foundation for Innovation and the Ontario Research Fund for supporting the Canadensys infrastructure that enabled TRT to database, image and curate voucher specimens for this project. Part of the laboratory work for this project was undertaken at the Canadian Centre of DNA Barcoding at Guelph where DNA barcoding was funded by the Government of Canada through Genome Canada and the Ontario Genomics Institute (2008-OGI-ICI-03). Fieldwork, and labwork in Toronto and Kelowna, were supported by a Natural Sciences and Engineering Research Council of Canada Strategic Research Project grant, ‘Hawthorn as a new Canadian agroforestry crop for functional foods and nutraceuticals,’ STPGP 381073-09 to T.A.D., S.S., P.R.S. and Spencer D. Proctor, who is a co-PI on the Strategic Research Project grant, but was not involved in the research that resulted in this manuscript. The generous support of the Louise Hawley Stone Charitable Trust enabled the Royal Ontario Museum Green Plant Herbarium to accept the University of Western Ontario's gift of the J.B. Phipps Hawthorn Research Collection. Additional support from the ROM Reproductions, Acquisitions, and Research Fund and the Department of Natural History, as well as from the Department of Ecology and Evolutionary Biology at the University of Toronto is also gratefully acknowledged.

## Contributions by the Authors

M.Z. designed the initial sampling, and carried out the sequencing and data analyses represented in Fig. 1 and **Supporting Information Figs S1–S4**. N.T. carried out most of the flow cytometric analyses and curates these data. M.K. sampled additional individuals and carried out all of the DNA barcode sequencing. J. Lee took part in fieldwork and record-keeping for the project. J. Lund and P.R.S. collected and analysed the metabolomics data used here. S.S. and T.A.D. advised on sampling and data analysis. The manuscript was prepared by M.Z. and T.A.D., based on contributions from, and revisions by, all of the authors.

## Conflict of Interest Statement

J. Lee is on the board of directors of the Naturally Grown Herb & Spice Producers Cooperative (HerbPro), and chairs their value added committee that is focussed on development of hawthorn-based products.

## Supporting Information

The following additional information is available in the online version of this article –

**Table S1.** Voucher information and GenBank accession numbers for the three plastid DNA barcode loci, and nrITS2 amplified directly from genomic DNA as a DNA barcode locus, used in the current study (**Supporting Information—Figs S1 and S2**; BOLD Dataset NAMCRAT; further details of the barcode sequences are available at dx.doi.org/10.5883/DS-NAMCRAT).

**Table S2.** Phylogenetic analysis of 14 *Crataegus* plastid DNA loci (Fig. 1). Voucher information and GenBank accession numbers for 11 supplementary plastid DNA loci and the three plastid DNA barcode loci (**Supporting Information—Table S1**; further details of the barcode sequences are available at dx.doi.org/10.5883/DS-NAMCRAT).

**Table S3.** *Crataegus* voucher information and GenBank accession numbers for the AT1 region used in the current study.

**Table S4.** *Crataegus* voucher information and GenBank accession numbers for the PEPC region used in the current study.

**Table S5.** Models of nucleotide evolution in *Crataegus* selected using the AIC for analyses of plastid (Fig. 1; **Supporting Information—Table S2**) and nuclear **[see Supporting Information—Figs S3 and S4**; **Supporting Information—Tables S3 and S4****]** markers.

**Table S6.** Comparison of *Crataegus* sequence variation between different markers utilized for the phylogenetic analyses (Fig. 1; **Supporting Information—Figs S3 and S4**).

**File S1; AT1 and PEPC Supporting Information.** Low-copy nuclear gene methods, data analyses and results.

**Figure S1.** Neighbour-Joining tree for three concatenated *Crataegus* plastid DNA barcode markers (*mat*K, *rbcL*a and *psb*A*-trn*H). The numbers preceding the taxonomic information are *Crataegus* sample identifiers (**Supporting Information—Table S1**; BOLD Dataset NAMCRAT). Ploidy level for the vouchered individual is given where known (in parentheses, if voucher information unavailable, but species has a single characteristic ploidy level). Scale for branch lengths in substitutions per site. Leaves are coloured to show *Crataegus* infrageneric classification **[see** **Supporting Information—Table S1****]**, with labelling of clades following that in Lo *et al*. (2007): blue rectangles, section *Mespilus* (clade A_1_); blue text, section *Brevispinae* (clade A_2_); red rectangles, section *Crataegus* (clade B); orange text, sections *Coccineae* and *Macracanthae* (clade D); purple rectangles, series *Cerrones* (clade E_1_); purple text, series *Douglasianae* (clade E_2_) and green rectangles, section *Sanguineae* (clade E_3_). Clade C as described earlier (Lo *et al*. 2007), and in the text.

**Figure S2.** Neighbour-Joining tree for ITS2 amplified directly from *Crataegus* genomic DNA as a DNA barcode locus. The numbers preceding the taxonomic information are *Crataegus* sample identifiers (**Supporting Information—Table S1**; BOLD Dataset NAMCRAT). Ploidy level for the vouchered individual is given where known (in parentheses, if voucher information unavailable, but species has a single characteristic ploidy level). This figure includes sequences (filled diamonds) from four accessions of *C. douglasii* (TRT157, 175, 177 and 184; **Supporting Information—Table S1**), two of *C. orbicularis* (MKTRT587, 588; **Supporting Information—Table S1**) and one of *C. sheila-phippsiae* (MKTRT617; **Supporting Information—Table S1**) with sequences that were not barcode compliant, but nevertheless could be fitted into an alignment. Scale for branch lengths in substitutions per site. Leaves are coloured, and clades are labelled, as described for **Supporting Information—Fig. S1**.

**Figure S3**. The Bayesian phylogram for AT1, the PPR homologue of the AT1G09680 gene in *Arabidopsis*. Branch support values are indicated as PP above branches, and bootstrap (BS) values below branches. Branches with PP <0.5 are shown as polytomies. BS values <50 % are not shown. Asterisks indicate branches retained in the strict consensus of 243470 trees. Scale for branch lengths in substitutions per site. The numbers preceding the taxonomic information are *Crataegus* sample identifiers **[see Supporting Information—Table S3]** and clone numbers (separated by a hyphen). Clades a–d are described in the **Supporting Information Text****—File S1**. Leaves are coloured as described for **Supporting Information—Fig. S1**.

**Figure S4**. The Bayesian phylogram for PEPC sequences. Branch support values are indicated as PP above branches. Branches with PP <0.5 are shown as polytomies (PP values <0.7 are not shown). Asterisks indicate branches retained in the strict consensus of 102 420 trees. Leaves are coloured as in **Supporting Information—Fig. S3**. Scale for branch lengths in substitutions per site. S, short paralog; L, long paralog. The numbers preceding the taxonomic information are *Crataegus* sample identifiers **[see Supporting Information—Table S4]** and clone numbers (separated by hyphen). Clade a is described in the **Supporting Information Text—****File S1**. Leaves are coloured as described for **Supporting Information—Fig. S1**.

Additional Information
