# Supplementary material for: DNA barcodes from four loci provide poor resolution of taxonomic groups in the genus Crataegus
Source: AoB Plants. 2015 Apr 29;7:plv045. doi: 10.1093/aobpla/plv045 (PMC4480070; doi:10.1093/aobpla/plv045)
Supplement: Additional Information [file supp_7_plv045_index.html]

DNA barcodes from four loci provide poor resolution of taxonomic groups in the genus Crataegus — Additional Information 

# DNA barcodes from four loci provide poor resolution of taxonomic groups in the genus *Crataegus*

## Additional Information

Additional Information

- Supplementary Data - doc file
- Supplementary Figure 1 - eps file
- Supplementary Figure 2 - eps file
- Supplementary Figure 3 - eps file
- Supplementary Figure 4 - eps file
- Supplementary Table 1 - doc file
- Supplementary Table 2 - doc file
- Supplementary Table 3 - doc file
- Supplementary Table 4 - doc file
- Supplementary Table 5 - doc file
- Supplementary Table 6 - doc file
