# Supplementary material for: DNA barcodes from four loci provide poor resolution of taxonomic groups in the genus Crataegus
Source: AoB Plants. 2015 Apr 29;7:plv045. doi: 10.1093/aobpla/plv045 (PMC4480070; doi:10.1093/aobpla/plv045)
Supplement: Additional Information [file supp_plv045_plv045supp_Data.doc]

**Molecular methods and results for the low-copy nuclear genes:**

Amplification of the partial Phosphoenolpyruvate Carboxylase (PEPC) gene used the primers provided in Lo *et al.* (2009). Pentatricopeptide region (PPR) homologue to AT1G09680 gene in Arabidopsis, hereafter AT1, was amplified using forwards (5'-ACTSCACMATGGCCCACTTCCT-3') and reverse (5'-CWGGGTTTCCATGCTTGCAATG-3') primers designed here. The voucher information is provided in Tables S3 and S4. The amplicons were purified using Bio Basic EZ-10 Spin Column PCR purification Kit (Bio Basic Inc., Markham, Canada). By direct sequencing of PEPC amplicons, variation in both the sequence length and additive polymorphic sites (APS) was detected. Only APS was found in the tracers of direct sequencing of AT1. To ensure that all different copies of PEPC and AT1 were amplified, different sets of PCR amplifications, e.g. highly denaturing PCR condition with adding DMSO (dimethylsulphoxide; 4% final concentration), was tested (see Zarrei *et al.* 2009, 2012, 2014, for details). Post-PCR steps for all markers were followed as in Zarrei et al. 2014. Cycle sequencing reactions were performed using the BigDye® Terminator v3.1 kit (Applied Biosystems Inc., Foster City, CA, USA). Cleaned cycle sequencing products were sequenced on an ABI 3730 (Applied Biosystems) DNA Analyzer at the Royal Ontario Museum (Toronto, Canada). Sequences were proofed and edited using Geneious Pro. v.5.6 (Drummond *et al.* 2012) and assembled using BioEdit v.7.0.5.3 (Hall 1999) or Geneious Pro. v.5.6 (Drummond *et al.* 2012).

**Data analyses**

All DNA sequences obtained from each accession for the AT1 and PEPC regions were collapsed into haplotypes using DnaSP v.5 (Librado and Rozas 2009). The number of haplotypes for each accessions is provided in Tables S3 and S4. The reduce matrices consist of 399 and 281 haplotypes for AT1 and PEPC, respectively. A recombination test was performed using RDP4 Beta 4.14 (recombination detection program, Martin *et al.* 2010). No signs of recombination were found in the AT1. However, four sequences with signs of recombination were removed from the PEPC datasets before further analyzing. These sequences were probably as a result of sequence recombination caused by PCR errors which is expected to be common in polyploids (see Lo *et al.* 2010 and references therein for the discussion on sequence recombination and PCR errors).

Because of presence of several haplotypes in the genome of each individual for AT1 and PEPC, we were unable to generate a concatenated matrix containing the DNA sequences for the plastids and low-copy nuclear genes. Each marker, therefore, was analyzed individually.

Two different phylogenetic analyses were run for each nuclear regions: (i) maximum parsimony (MP) analyses using PAUP* v. 4b10 (Swofford 2002), and (ii) Bayesian analyses (BI; Yang and Rannala 1997) using MrBayes v. 3.2.0 (Ronquist and Huelsenbeck 2003; Ronquist *et al.* 2010). The details of each analyses is followed as in Zarrei *et al.* 2009, 2012, 2014. Indels were coded as separate presence/absence characters for PEPC datasets (no indels in AT1 sequences) using SeqState version 1.4.1 (Müller 2005) with modified complex coding option originally described by Simmons and Ochoterena (2000) and appended to the end of matrices.

A safe deletion rule, originally introduced as Safe Taxonomic Reduction (STR) strategy by Wilkinson (1995) and modified by Zarrei *et al.* (2012), was used to reduce the size of the original dataset (see Zarrei *et al.* 2014 for details). The AT1 and PEPC matrices were reduced to 131 and 168 OTUs for final parsimony and BI analyses.

In the parsimony analysis, the character state changes were equally weighted and character changes were interpreted under ACCTRAN optimization (Agnarsson and Miller 2008). A two-stage strategy of Fitch parsimony (Fitch 1971) search was undertaken following Stefanović *et al.* (2007). The phylogenetic reliability was assessed using nonparametric bootstrapping. The bootstrap support (BS) was estimated using 1,000 bootstrap pseudoreplicates with simple taxon addition and TBR swapping but permitting only ten trees per replicate to be held. The consistency index (CI), rescaled consistency index (RC), and Farris’s (1989) retention index (RI) were calculated to measure the amount of homoplasy in the dataset.

The best fit model for each region in the AT1, and PEPC is provided in Table S5. These models were selected by Akaike information criterion (AIC; Akaike 1974), as implemented in MrModeltest v. 2.3 (Nylander 2004).

For the Bayesian analyses, two simultaneous runs with four chains each were run for 50 and 20 million generations for AT1 and PEPC datasets, respectively. In each run, every 2,000^th^ and 1,000^th^ trees was sampled for AT1, and PEPC, respectively. The completion of the Bayesian analysis was determined when the average standard deviation of split frequencies ≤ 0.05 (Ronquist and Huelsenbeck 2003) for the combined two runs was assumed and the complete convergence between the Bayesian MCMC runs was reached. Convergence of an independent search was further explored by plotting likelihood scores *vs* generations using the program Tracer v1.5 (Rambaut and Drummond 2007). The burn-in phase for each run – the first 25% of sampled trees was discarded during computing the phylogram consensus tree based on the average branch lenghts (50% majority rule) of the remaining trees (37,500, and 30,000 trees for the AT1 and PEPC, respectively) using *sumt* command implemented in MrBayes. Support for Bayesian topologies was estimated using node posterior probabilities (PP) from the posterior distribution of topologies.

**Results:**

A total of 462 DNA sequences (0.563 meganucleotides) comprising of 399 haplotypes were generated from AT1 (Table S3). Open reading frames were identified for all sequences suggesting functionality of retrieved sequences. In the Bayesian phylogram (Fig. S3) *Amelanchier* is supported as outgroup with PP=1 and BS=94. All clones of *C. brachyacantha* formed only one clade with absolute PP and BS values (Fig. S3). Similarly, *C. germanica* haplotypes formed a clade with PP=1 and BS=99 (Fig. S3). These two species formed a clade with PP=0.97 and BS=77, sister to the rest of ingroup with absolute PP and BS values (Fig. S3). Most of other ingroup haplotypes have PP < 0.95 and BS < 70 (Fig. S3). However, there are some clades with moderate PP and BS support, such as the grouping of *C. chrysocarpa* haplotypes with *C. gaylussacia* and diploid and triploid *C. suksdorfii* in clades a, b, and c. Some haplotypes of *C. chrysocarpa* were grouped with *C. castlegarensis* and *C. okennonii* in clade d with PP=1 and BS=80 (Fig. S3).

A total of 317 DNA sequences (0.253 meganucleotides) comprising 281 haplotypes were sequenced for PEPC. DNA sequences were designated here as long (L; mean sequence length= 818.5) and short (S; mean sequence length= 731) paralogues based on their length (indels) and mean nucleotide divergence (Table S5). The short copy has higher mean sequence divergence (mean overall sequence divergence = 0.064) than the large copy (mean overall sequence divergence = 0.018) resulting in a better resolution between different haplotypes in the small copy in both MP and BI trees. The duplication of the PEPC into two copies (L-copy and S-copy) is well supported with absolute PP and BS values (Fig. S4). The resolution among the haplotypes in the L-copy was very low (mostly with PP and BS < 50 percent). However, there are a few clades with PP > 0.95, but with BS < 50 (Fig. S4). These clades contain sequences from diploid *C. suksdorfii* and tetraploid *C. chrysocarpa*. *Amelanchier* haplotypes are supported as the outgroup with PP=1 and BS=94. While major clades in the small copy are well supported, these clades have limited taxonomic meaning as haplotypes from different sections are intermixed with each other in these well-supported clades. For example, clade A with PP=1 and BS=87 comprises haplotypes from *C. pentagyna*, *C. chrysocarpa*, and *C. douglasii*, which are from three different sections, of which at least one (*C.* sect. *Crataegus*) is not known to be involved in hybridizations with the other two species.

**Discussion:**

For discussion please refer to the main text of this paper.

**References:**

Akaike H. 1974. A new look at the statistical model identification. *IEEE Transactions on Automatic Control* **19**:716–723.

Agnarsson I, Miller JA. 2008. Is ACCTRAN better than DELTRAN? *Cladistics* **24**:1–7.

Drummond AJ, Ashton B, Buxton S, Cheung M, Cooper A, Duran C, Field M, Heled J, Kearse M, Markowitz S, Moir R, Stones-Havas S, Sturrock S, Thierer, T, Wilson A. 2012. *Geneious v5.6*, available from http://www.geneious.com.

Farris JS. 1989. The retention index and the rescaled consistency index. *Cladistics* **5**:417–419.

Fitch WM.1971.Towards defining the course of evolution: minimum change for a specific tree topology. *Systematic Zoology* **20**:406–426.

Hall TA. 1999. BioEdit: a user-friendly biological sequence alignment editor and analysis program for Windows 95/98/NT. *Nucleic Acids Symposium Series* **41**:95–98.

Librado P, Rozas J. 2009. DnaSP v5: a software for comprehensive analysis of DNA polymorphism data. *Bioinformatics* **25**:1451–1452.

Lo EYY, Stefanovic´ S, Christensen KI, Dickinson TA. 2009. Evidence for genetic association between East Asian and Western North American *Crataegus* L. (Rosaceae) and rapid divergence of the Eastern North American lineages based on multiple DNA sequences. *Molecular Phylogenetics and Evolution* **51**:157–168.

LoEYY, Stefanovic´ S, DickinsonTA. 2010.Reconstructing reticulation history in a phylogenetic framework and the potential of allopatric speciation driven by polyploidy in an agamic complex in Crataegus (Rosaceae). *Evolution* **64**:3593–3608.

MartinDP, Lemey P, Lott M, MoultonV, Posada D, Lefeuvre P. 2010. RDP3: a flexible and fast computer program for analyzing recombination. *Bioinformatics* **26**:2462–2463.

Müller K. 2005. SeqState – primer design and sequence statistics for phylogenetic DNA data sets. *Applied Bioinformatics* **4**:65–69.

Nylander JAA. 2004. MrModeltest v2.3 program. Distributed by the author. Evolutionary Biology Centre, Uppsala University.

Rambaut A, Drummond AJ. 2007. *Tracer v1.4*. Available from http://tree.bio.ed.ac.uk/software/tracer/.

Ronquist F, Huelsenbeck JP. 2003. MrBayes 3: Bayesian phylogenetic inference under mixed models. *Bioinformatics* **19**:1572–1574.

Ronquist F, van deMark P, Huelsenbeck JP. 2010. Bayesian phylogenetic analyses using MrBayes. In: Lemey P, Salemi M, Vandamme AM, eds. *The phylogenetic handbook: a practical approach to phylogenetic analysis and hypothesis testing*. Cambridge: University Cambridge Press, 2010–2066.

Simmons MP, Ochoterena H. 2000. Gaps as characters in sequence-based phylogenetic analyses. *Systematic Biology* **49**:369–381.

Stefanovic´ S, Kuzmina M, Costea M. 2007. Delimitation of major lineages within *Cuscuta* subgenus *Grammica* (Convolvulaceae) using plastid and nuclear DNA sequences. *American Journal of Botany* **94**:568–589.

Swofford DL. 2003. *PAUP*: phylogenetic analysis using parsimony (*and Other Methods). Version 4.0b10 for Macintosh (PPC/Altivec) ed*. Sunderland MA: Sinauer Associates.

Wilkinson M.1995.Coping with missing entries in phylogenetic inference using parsimony. *Systematic Biology* **44**:501–514.

Yang Z, Rannala B. 1997. Bayesian phylogenetic inference using DNA sequences: a Markov chain Monte Carlo method. *Molecular Biology and Evolution* 14:717–724.

Zarrei M, Wilkin P, Fay MF, Ingrouille MJ, Zarre S, Chase MW. 2009. Molecular systematics of *Gagea* and *Lloydia* (Liliaceae; Liliales): implications of analyses of nuclear ribosomal and plastid sequences for infrageneric classification. *Annals of Botany* **104**:125–142.

Zarrei M, Wilkin P, Ingrouille JM, Leitch IJ, Buerki S, Fay MF, Chase MW. 2012. Speciation and evolution in the *Gagea* *reticulata* species complex (Tulipeae; Liliaceae). *Molecular Phylogenetics and Evolution* **62**:624–639.

Zarrei M, Stefanović S, Dickinson TA. 2014. Reticulate evolution in North American black-fruited hawthorns (*Crataegus* section *Douglasia*; Rosaceae): evidence from nuclear ITS2 and plastid sequences. *Annals of Botany* **114**:253–269.
