# Supplementary material for: DNA barcodes from four loci provide poor resolution of taxonomic groups in the genus Crataegus
Source: AoB Plants. 2015 Apr 29;7:plv045. doi: 10.1093/aobpla/plv045 (PMC4480070; doi:10.1093/aobpla/plv045)
Supplement: Additional Information [file supp_plv045_plv045supp_table1.doc]

| Table S1. Vouchers for *Crataegus* DNA barcode sequences arranged in taxonomic order by section and series (boldface). Letters (A—E) identify major clades in the phylogeny obtained by Lo et al. (2007). Ploidy levels (*x* = 17) in parentheses are based on Talent & Dickinson (2005) and are believed to be generally true of the taxon in question. Other ploidy values are based on determinations made on tissue from the individual represented by the voucher. Collection details, BOLD identifiers (TRTnnn, MKTRTnnn), and TRT (TRTnnnnnnnn) and GenBank accession numbers; further details of the barcode sequences are available at dx.doi.org/10.5883/DS-NAMCRAT. Collecting localities in the Canada and the United States are summarized as province or state abbreviations, county or equivalent, and decimal latitude and longitude. Accessions with TRT and BOLD numbers in bolded italics were part of the sample used to prepare Fig. 1 (Table S2). | | | | | | | | | | | | | | | |
| --- | --- | --- | --- | --- | --- | --- | --- | --- | --- | --- | --- | --- | --- | --- | --- |
| **Section** | | | | | | | | | | | | | | | |
|  | **Series** | **Ploidy** | **Collector and Collector’s Number** | | **Date** | **BOLD** | **TRT** | **Locality** | | | **ITS2** | ***psb*A*-trn*H** | ***rbcLa*** | ***mat*K** | **Compliant** |
| A. ***Mespilus*** T.A. Dickinson & E.Y.Y. Lo | | | |  |  |  |  |  | | |  |  |  |  |  |
|  | *C. germanica* (L.) K. Koch | | |  |  |  |  |  | | |  |  |  |  |  |
|  |  | NA | Dickinson, T.A.; Dickinson, L.M. | 2000-54 | 21-May-2000 | TRT038 | TRT00026649 | MA, Suffolk Co. Arnold Arboretum (42.2977, -71.1282) 727-89C | | | KC861802 | KC251569 | KC251296 |  | Yes |
|  |  | NA | Dickinson, T.A. | s.n. | 16-Jun-1999 | TRT097 | TRT00026642 | CA, Alameda Co. U. of California Botanical Garden (37.875, -122.25) 78.0184 | | | KC861808 | KC251576 | KC251303 |  | Yes |
|  |  | NA | Dönmez, A.A. | AAD11457 | 21-Jun-2003 | TRT084 | voucher in HUB | Turkey Ardanuc, Artvin (41.161, 42.0633) | | | KC861801 | KC251570 | KC251297 |  | Yes |
|  |  | NA | Dönmez, A.A. | AAD11600 | 06-Jul-2003 | TRT079 | voucher in HUB | Turkey Kirklareli, (41.8841, 27.9853) | | | KC861804 | KC251567 | KC251294 |  | Yes |
|  |  | NA | Dönmez, A.A. | AAD11619 | 07-Jul-2003 | TRT088 | voucher in HUB | Turkey Istanbul, (41.3, 28.7) | | | KC861806 | KC251574 | KC251301 |  | Yes |
|  |  | NA | Dönmez, A.A. | AAD11656 | 07-Jul-2003 | TRT089 | voucher in HUB | Turkey Bursa, (40.194, 29.0442) | | |  | KC251568 | KC251295 |  | Yes |
|  |  | NA | Dönmez, A.A. | AAD11660 | 07-Jul-2003 | TRT090 | voucher in HUB | Turkey Goynuk, Bolu (40.4483, 30.9016) | | | KC861807 | KC251572 | KC251299 |  | Yes |
|  |  | NA | Dönmez, A.A. | AAD11687 | 07-Jul-2003 | TRT087 | voucher in HUB | Turkey Bolu, (40.6515, 31.6359) | | | KC861805 | KC251573 | KC251300 |  | Yes |
|  |  | 2x | Hess, W.; Linden, M. | 6210V93 | 30-Sep-1993 | TRT074 | TRT00020699 | IL, DuPage Co. Morton Arboretum (41.814, -88.064) 682-80 | | |  | KC251571 | KC251298 |  | Yes |
|  |  | 2x | Hess, W.; Linden, M. | 6216V93 | 30-Sep-1993 | ***TRT075*** | ***TRT00020706*** | IL, DuPage Co. Morton Arboretum (41.814, -88.064) 645-80 | | | KC861803 | KC251575 | KC251302 |  | Yes |
| A. ***Phippsara*** T.A. Dickinson & E.Y.Y. Lo | | | |  |  |  |  |  | | |  |  |  |  |  |
|  | *C.* ×*canescens* (J.B. Phipps) T.A. Dickinson & E.Y.Y. Lo | | |  |  |  |  |  | | |  |  |  |  |  |
|  |  | 3x | Dickinson, T.A.; Talent, N.; Nguyen, S.; Witsell, T.; Warriner, M. | 2003-35 | 23-Apr-2003 | TRT080 | TRT00020698 | AR, Prairie Co. (34.6322, -91.5917) | | |  | KC251502 | KC251230 | KC251007 | Yes |
|  |  | 3x | Dickinson, T.A.; Talent, N.; Nguyen, S.; Witsell, T.; Warriner, M. | 2003-36 | 23-Apr-2003 | TRT081 | TRT00020697 | AR, Prairie Co. (34.6322, -91.5917) | | |  | KC251499 | KC251227 | KC251004 | Yes |
|  |  | 3x | Dickinson, T.A.; Talent, N.; Nguyen, S.; Witsell, T.; Warriner, M. | 2003-37 | 23-Apr-2003 | TRT082 | TRT00020696 | AR, Prairie Co. (34.6322, -91.5917) | | |  | KC251501 | KC251229 | KC251006 | Yes |
|  |  | 3x | Dickinson, T.A.; Talent, N.; Nguyen, S.; Witsell, T.; Warriner, M. | 2003-38 | 23-Apr-2003 | TRT086 | TRT00020693 | AR, Prairie Co. (34.6322, -91.5917) | | |  | KC251500 | KC251228 | KC251005 | Yes |
|  |  | 3x | Dickinson, T.A.; Talent, N.; Nguyen, S.; Witsell, T.; Warriner, M. | 2003-39 | 23-Apr-2003 | TRT085 | TRT00020694 | AR, Prairie Co. (34.6322, -91.5917) | | |  | KC251504 | KC251232 | KC251009 | Yes |
|  |  | 3x | Dickinson, T.A.; Talent, N.; Nguyen, S.; Witsell, T.; Warriner, M. | 2003-40 | 23-Apr-2003 | TRT083 | TRT00020695 | AR, Prairie Co. (34.6322, -91.5917) | | |  | KC251503 | KC251231 | KC251008 | Yes |
| A. ***Brevispinae*** Beadle ex Schneider | | | |  |  |  |  |  | | |  |  |  |  |  |
|  | ***Brevispinae*** (Beadle ex Schneider) Rehder | | |  |  |  |  |  | | |  |  |  |  |  |
|  | *C. brachyacantha* Sarg. & Engelm. | | |  |  |  |  |  | | |  |  |  |  |  |
|  |  | 2x+ | Dickinson, T.A.; Reid, C. | 2011-2 | 24-Mar-2011 | TRT248 | TRT00019453 | LA, DeSoto Parish (32.3093, -93.8058) | | | KC861777 | KC251493 | KC251220 | KC251002 | No |
|  |  | 2x+ | Dickinson, T.A.; Reid, C. | 2011-2 | 24-Mar-2011 | TRT219 | TRT00019453 | LA, DeSoto Parish (32.3093, -93.8058) | | | KC861778 | KC251498 | KC251226 | KC251003 | Yes |
|  |  | 2x+ | Dickinson, T.A.; Reid, C. | 2011-3 | 24-Mar-2011 | TRT249 | TRT00019454 | LA, DeSoto Parish (32.3039, -93.8022) | | | KC861776 | KC251492 | KC251219 | KC251001 | Yes |
|  |  | 2x+ | Dickinson, T.A.; Talent, N.; Nguyen, S. | 2003-32 | 21-Apr-2003 | ***TRT128*** | ***TRT00000023*** | LA, Sabine Parish (31.8433, -93.7736) | | | KC861773 | KC251491 | KC251218 | KC251000 | Yes |
|  |  | 2x+ | Reid, C. | 5201 | 16-Aug-2004 | TRT070 | TRT00000026 | LA, Ouachita Parish (32.3958, -92.0256) | | |  | KC251497 | KC251224 |  | Yes |
|  |  | NA | Reid, C. | 5202 | 16-Aug-2004 | TRT069 | TRT00000025 | LA, Ouachita Parish (32.3956, -92.0256) | | |  |  | KC251225 |  | Yes |
|  |  | 2x+ | Reid, C. | 5203 | 16-Aug-2004 | TRT073 | TRT00000028 | LA, Morehouse Parish (32.67, -91.7358) | | | KC861775 | KC251494 | KC251221 |  | Yes |
|  |  | 2x+ | Reid, C. | 5206 | 16-Aug-2004 | TRT071 | TRT00000027 | LA, Ouachita Parish (32.4761, -92.0831) | | | KC861774 | KC251496 | KC251223 |  | Yes |
|  |  | 2x+ | Reid, C. | 5207 | 16-Aug-2004 | TRT072 | TRT00000024 | LA, Ouachita Parish (32.4761, -92.0831) | | |  | KC251495 | KC251222 |  | Yes |
| B. ***Crataegus*** | | | |  |  |  |  |  | | |  |  |  |  |  |
|  | ***Crataegus*** | | |  |  |  |  |  | | |  |  |  |  |  |
|  | *C. laevigata* (Poir.) DC. | | |  |  |  |  |  | | |  |  |  |  |  |
|  |  | NA | Zika, P.F. | 18470 | 13-Jun-2003 | TRT148 | TRT00000386 | WA, San Juan Co. (48.6, -123) | | | KC861821 | KC251590 | KC251317 | KC251073 | Yes |
|  |  | 2x | Zika, P.F. | 18472 | 13-Jun-2003 | TRT151 | TRT00002174 | WA, San Juan Co. (48.5983, -122.995) | | | KC861822 | KC251592 | KC251319 | KC251075 | Yes |
|  |  | 4x | Zika, P.F. | 18473 | 13-Jun-2003 | TRT150 | TRT00002894 | WA, San Juan Co. (48.5967, -122.992) | | | KC861823 | KC251591 | KC251318 | KC251074 | Yes |
|  | *C. monogyna* Jacq. | | |  |  |  |  |  | | |  |  |  |  |  |
|  |  | 2x | Coughlan, J.; Zarrei, M.; Shaw, C. | JC001 | 05-Jul-2011 | TRT274 | TRT00020101 | CA, Humboldt (40.4728, -123.879) | | | KC861835 | KC251612 | KC251338 | KC251093 | Yes |
|  |  | (2x) | Coughlan, J.; Zarrei, M.; Shaw, C. | JC003 | 05-Aug-2011 | TRT275 | TRT00020102 | CA, Sonoma (38.4037, -122.848) | | |  | KC251615 | KC251341 | KC251096 | Yes |
|  |  | (2x) | Dickinson, T.A.; Shaik, M. | 2008-26 | 08-Mar-2008 | TRT209 | TRT00002452 | BC, Central Kootenay R.D. (49.3347, -117.701) | | | KC861840 | KC251617 | KC251343 | KC251098 | Yes |
|  |  | (2x) | Dickinson, T.A.; Talent, N. | 2003-79 | 31-May-2003 | TRT109 | TRT00000395 | ON, Middlesex Co. (43.0726, -81.4019) | | | KC861839 | KC251614 | KC251340 | KC251095 | Yes |
|  |  | (2x) | Lo, E.Y.Y.; Dickinson, T.A.; Nguyen, S. | EL-108 | 06-Nov-2004 | TRT143 | TRT00000417 | OR, Columbia Co. (45.7308, -122.767) | | |  | KC251619 | KC251345 | KC251099 | Yes |
|  |  | 2x | Lo, E.Y.Y.; Dickinson, T.A.; Nguyen, S. | EL-74 | 10-Jun-2004 | TRT007 | TRT00000416 | OR, Linn Co. (44.333, -123.122) | | |  | KC251618 | KC251344 |  | Yes |
|  |  | 2x | Lo, E.Y.Y.; Dickinson, T.A.; Nguyen, S. | EL-80 | 10-Jun-2004 | TRT005 | TRT00000413 | OR, Linn Co. (44.333, -123.122) | | |  | KC251621 | KC251347 | KC251101 | Yes |
|  |  | 2x | Lo, E.Y.Y.; Dickinson, T.A.; Nguyen, S. | EL-83 | 10-Jun-2004 | TRT006 | TRT00000415 | OR, Linn Co. (44.333, -123.122) | | | KC861837 | KC251620 | KC251346 | KC251100 | Yes |
|  |  | 3x | Love, R.M. | C-2003-25 | 13-May-2003 | TRT030 | TRT00000420 | OR, Lane Co. (44.0424, -123.149) | | |  | KC251611 | KC251337 | KC251092 | Yes |
|  |  | (2x) | Purich, M. | 098 |  | TRT211 | TRT00029476 | ON, Durham R.M. (43.9016, -78.6846) | | | KC861841 | KC251610 | KC251336 | KC251091 | Yes |
|  |  | (2x) | Purich, M.A.; Talent, N. | 082 | 06-Mar-2004 | TRT108 |  | ON, Durham R.M. (43.9019, -78.6829) | | | KC861838 | KC251616 | KC251342 | KC251097 | Yes |
|  |  | (2x) | Purich, M.A.; Talent, N. | 083 | 06-Mar-2004 | TRT190 | TRT00002248 | ON, Durham R.M. (43.9019, -78.6829) | | | KC861836 | KC251613 | KC251339 | KC251094 | Yes |
|  | *C. songarica* K. Koch | | |  |  |  |  |  | | |  |  |  |  |  |
|  |  | NA | Dickinson, T.A. | s.n. | 10-Sep-1999 | TRT028 | TRT00000423 | MA, Suffolk Co. Arnold Arboretum (42.3031, -71.1242) AA19865 | | |  | KC251673 | KC251400 | KC251138 | Yes |
|  | ***Orientales*** (C.K. Schneid.) Pojarkova | | |  |  |  |  |  | | |  |  |  |  |  |
|  | *C. heldreichii* Boiss. | | |  |  |  |  |  | | |  |  |  |  |  |
|  |  | 2x | Dickinson, T.A. | 2000-64 2003-61 | 20-May-2000 | TRT037 | TRT00002172 | MA, Suffolk Co. Arnold Arboretum (42.2977, -71.1282) 238-71A | | | KC861816 | KC251582 | KC251309 | KC251065 | Yes |
|  | ***Pentagynae*** (C.K. Schneid.) Russanov | | |  |  |  |  |  | | |  |  |  |  |  |
|  | *C. pentagyna* Waldst. & Kit. | | |  |  |  |  |  | | |  |  |  |  |  |
|  |  | NA | Christensen, K.I. | s.n. |  | TRT050 |  | Denmark , Materials grown in the Arboretum Hørsholm, Royal Agrichltural and Veterinary University from seed from Elborz, Challus Valley, Iran. (55.8699, 12.5029) 178-1974 | | | KC861852 | KC251637 | KC251362 | KC251111 | Yes |
|  |  | 2x | Christensen, K.I. | s.n. | 21-Mar-2005 | ***TRT043*** | ***TRT00001887*** | Denmark , Hørsholm, The Arboretum, Kirkegardsvey 3A (55.8699, 12.5029) 315-1993 | | | KC861851 | KC251636 | KC251361 | KC251110 | Yes |
|  |  | 2x | Christensen, K.I. | s.n. | 21-Mar-2005 | TRT091 | TRT00001886 | Denmark , Hørsholm, The Arboretum, Kirkegardsvey 3A (55.8699, 12.5029) 315-1993 | | | KC861853 | KC251638 | KC251363 | KC251112 | Yes |
|  |  | 2x | Christensen, K.I. | s.n. | 21-Mar-2005 | ***TRT098*** | ***TRT00001888*** | Denmark , Hørsholm, The Arboretum, Kirkegardsvey 3A (55.8699, 12.5029) 315-1993 | | |  | KC251635 | KC251360 |  | Yes |
|  | ***Pinnatifidae*** (Zabel ex C.K. Schneid.) Rehder | | |  |  |  |  |  | | |  |  |  |  |  |
|  | *C. pinnatifida* Bunge | | |  |  |  |  |  | | |  |  |  |  |  |
|  |  | NA | Romankova, T. | 2 | 15-Jun-2005 | ***TRT099*** | ***TRT00002365*** | Russia Primorsky Krai, Vladivostok, Botanical Garden of the Academy of Science. (43.1219, 131.871) | | | KC861858 | KC251643 | KC251368 | KC251117 | Yes |
|  |  | NA | Romankova, T. | 3 | 19-Jun-2005 | TRT092 | TRT00002363 | Russia Primorsky Krai, Lazovskiy Zapovednik (reserve), Koreyskaya Pad', Bistrushka River Valley (43.2547, 134.133) | | |  | KC251644 | KC251369 | KC251118 | Yes |
|  |  | NA | Romankova, T. | 6 | 19-Jun-2005 | TRT100 | TRT00002364 | Russia Primorsky Krai, Lazovskiy Zapovednik (reserve), Koreyskaya Pad', Bistrushka River Valley (43.2547, 134.133) | | | KC861859 | KC251645 | KC251370 | KC251119 | Yes |
| B. ***Coccitaegus*** K.I. Chr. & T.A.Dickinson | | | |  |  |  |  |  | | |  |  |  |  |  |
|  | ***Punctaegus*** K.I. Christ. & T.A.Dickinson | | |  |  |  |  |  | | |  |  |  |  |  |
|  | *C.* × *ninae*-*celottiae* K.I. Christ. & T.A.Dickinson | | |  |  |  |  |  | | |  |  |  |  |  |
|  |  | (2x) | Purich, M.A.; Talent, N. | MP84 | 3-Jun-2004 | ***TRT204*** | ***TRT00002249*** | ON, Durham R.M. (43.9016, -78.6846) | | | KC861845 | KC251623 | KC251349 | KC251103 | Yes |
|  |  | (2x) | Purich, M.A.; Talent, N. | MP85 | 3-Jun-2004 | ***TRT201*** | ***TRT00002250*** | ON, Durham R.M. (43.9016, -78.6846) | | | KC861843 | KC251622 | KC251348 | KC251102 | Yes |
|  |  | (2x) | Purich, M.A.; Talent, N. | MP86 | 3-Jun-2004 | ***TRT202*** | ***TRT00002251*** | ON, Durham R.M. (43.9016, -78.6846) | | | KC861844 | KC251625 | KC251351 | KC251104 | Yes |
|  |  | (2x) | Purich, M.A.; Talent, N.; Nguyen, S.; Lo, E. | MP73 | 18-May-2004 | TRT203 | TRT00002239 | ON, Toronto (43.6491, -79.5949) | | | KC861842 | KC251624 | KC251350 |  | Yes |
| B. ***Crataeguineae*** K.I. Chr. | | | |  |  |  |  |  | | |  |  |  |  |  |
|  | *C.* ×*dsungarica* (*C. almaatensis* ) | | |  |  |  |  |  | | |  |  |  |  |  |
|  |  | NA | Dickinson, T.A. | 2002-01 | 20-Jun-2002 | TRT117 | TRT00002407 | MA, Suffolk Co. Arnold Arboretum (42.2953, -71.1321) AA1196-65A | | |  | KC251485 | KC251210 | KC250992 | Yes |
|  |  | NA | Dickinson, T.A. | 2000-62 | 20-May-2000 | TRT118 | TRT00002171 | MA, Suffolk Co. Arnold Arboretum (42.3031, -71.1242) AA1196-65B | | | KC861767 | KC251486 | KC251211 | KC250993 | Yes |
| B. ***Hupehensis*** J.B. Phipps | | | |  |  |  |  |  | | |  |  |  |  |  |
|  | ***Hupehenses*** J.B. Phipps | | |  |  |  |  |  | | |  |  |  |  |  |
|  | *C. hupehensis* Sarg. | | |  |  |  |  |  | | |  |  |  |  |  |
|  |  | (2-3x) | Dickinson, T.A.; Dickinson, L.M. | s.n. | 21-May-2000 | TRT067 | TRT00001956-2 | MA, Suffolk Co. Arnold Arboretum (42.3031, -71.1242) AA356-81C | | |  | KC251584 | KC251311 | KC251067 | Yes |
|  |  | (2-3x) | Dickinson, T.A.; Dickinson, L.M. | s.n. | 21-May-2000 | TRT068 | TRT00001956-1 | MA, Suffolk Co. Arnold Arboretum (42.3031, -71.1242) AA356-81B | | | KC861817 | KC251583 | KC251310 | KC251066 | Yes |
| C. ***Crataegus*** | | | |  |  |  |  |  | | |  |  |  |  |  |
|  | ***Apiifoliae*** (Loudon) Rehder | | |  |  |  |  |  | | |  |  |  |  |  |
|  | C. marshallii Eggleston | | |  |  |  |  |  | | |  |  |  |  |  |
|  |  | NA | Dickinson, T.A.; Talent, N.; Nguyen, S. | 2003-05 | 18-Apr-2003 | TRT078 | TRT00000530 | AL, Dekalb Co. (34.5042, -85.624) | | | KC861831 |  |  | KC251086 | No |
|  |  | 2x | Dickinson, T.A.; Talent, N.; Nguyen, S.; Lance, R. | 2003-08 | 19-Apr-2003 | TRT077 | TRT00000371 | AL, Montgomery Co. (32.3097, -86.375) | | | KC861833 | KC251602 | KC251328 | KC251087 | Yes |
|  |  | 2x | Dickinson, T.A.; Talent, N.; Nguyen, S. | 2003-30 | 20-Apr-2003 | TRT126 | TRT00000372 | MS, Scott Co. (32.3, -89.2667) | | | KC861832 | KC251601 | KC251327 | KC251085 | Yes |
|  | ***Cordatae*** (Beadle) Rehder | | |  |  |  |  |  | | |  |  |  |  |  |
|  | *C. phaenopyrum* (Linnaeus f.) Medikus | | |  |  |  |  |  | | |  |  |  |  |  |
|  |  | NA | Dvorsky, K. | 22 | 29-Jun-2003 | MKTRT598 | TRT00003273 | ON, Middlesex Co. (43.0833, -81.3167) | | |  |  | KP050235 | KP050156 | Yes |
|  |  | NA | MacLeod, M.M.; Wells, T.C. | s.n. | 29-Sep-1984 | MKTRT600 | TRT00003280 | ON, Middlesex Co. (43.0167, -81.2667) | | |  |  | KP050260 |  | Yes |
|  |  | NA | Smith, P. | PS370 | 12-Jun-1986 | MKTRT599 | TRT00003284 | ON, Middlesex Co. (43.0167, -81.2333) | | |  |  | KP050281 |  | Yes |
|  | ***Microcarpae*** (Loudon) Rehder | | |  |  |  |  |  | | |  |  |  |  |  |
|  | *C. spathulata* Michx. | | |  |  |  |  |  | | |  |  |  |  |  |
|  |  | 2x | Dickinson, T.A.; Talent, N.; Nguyen, S. | 2003-06 | 18-Apr-2003 | TRT057 | TRT00001974 | GA, Floyd Co. (34.235, -85.3522) | | | KC861881 | KC251675 | KC251402 | KC251140 | Yes |
|  |  | 2x | Dickinson, T.A.; Talent, N.; Nguyen, S. | 2003-34 | 22-Apr-2003 | TRT058 | TRT00001975 | LA, Boissier Parish (32.5467, -93.5167) | | | KC861882 | KC251677 | KC251404 | KC251142 | Yes |
|  |  | 2x | Dickinson, T.A.; Talent, N.; Nguyen, S.; Lance, R. | 2003-11 | 19-Apr-2003 | TRT059 | TRT00001966 | AL, Montgomery Co. (32.3097, -86.375) | | | KC861880 | KC251676 | KC251403 | KC251141 | Yes |
| D. ***Coccineae*** Loudon | | | |  |  |  |  |  | | |  |  |  |  |  |
|  | ***Aestivales*** (Sarg.) Rehder | | |  |  |  |  |  | | |  |  |  |  |  |
|  | *C. opaca* Hooker & Arnott | | |  |  |  |  |  | | |  |  |  |  |  |
|  |  | 2x | Dickinson, T.A.; Talent, N.; Nguyen, S. | 2003-33 | 21-Apr-2003 | TRT020 | TRT00002042 | LA, Sabine Parish (31.8433, -93.7736) | | | KC861850 | KC251634 | KC251359 |  | Yes |
|  | ***Apricae*** J.B. Phipps | | |  |  |  |  |  | | |  |  |  |  |  |
|  | *C. extraria* Beadle | | |  |  |  |  |  | | |  |  |  |  |  |
|  |  | 3x | Lance, R.W. | 3571 | 16-Apr-2011 | TRT261 | TRT00021221 | SC, McCormick Co. (33.6855, -82.1626) | | |  | KC251560 | KC251286 | KC251052 | Yes |
|  | *C. sororia* Beadle | | |  |  |  |  |  | | |  |  |  |  |  |
|  |  | 4x | Lance, R.W. | 3561 | 16-Apr-2011 | TRT258 | TRT00021255 | SC, McCormick Co. (33.6709, -82.1831) | | | KC861879 | KC251674 | KC251401 | KC251139 | Yes |
|  | *C. visenda* Beadle | | |  |  |  |  |  | | |  |  |  |  |  |
|  |  | 3x | Lance, R.W. | 3563b | 16-Apr-2011 | TRT260 |  | SC, McCormick Co. (33.6815, -82.2021) | | | KC861911 | KC251724 | KC251449 |  | Yes |
|  |  | 3x | Lance, R.W. | 3564 | 16-Apr-2011 | TRT253 | TRT00021257 | SC, McCormick Co. (33.6815, -82.2021) | | | KC861910 | KC251723 | KC251448 | KC251175 | Yes |
|  | ***Bracteatae*** (Sarg.) Rehder | | |  |  |  |  |  | | |  |  |  |  |  |
|  | *C. ashei* Beadle | | |  |  |  |  |  | | |  |  |  |  |  |
|  |  | 4x | Dickinson, T.A.; Talent, N.; Nguyen, S. | 2002-06 | 20-Apr-2003 | TRT066 | TRT00000054 | MS, Scott Co. (32.359, -89.555) | | |  | KC251466 | KC251191 | KC250974 | Yes |
|  |  | 4x | Dickinson, T.A.; Talent, N.; Nguyen, S. | 2003-29 | 20-Apr-2003 | TRT062 | TRT00000050 | MS, Scott Co. (32.3, -89.2667) | | |  | KC251488 | KC251214 | KC250996 | Yes |
|  |  | NA | Dickinson, T.A.; Talent, N.; Nguyen, S.; Lance, R. | 2003-14 | 19-Apr-2003 | TRT060 | TRT00000053 | AL, Lowndes Co. (32.2611, -86.6486) | | |  | KC251490 | KC251217 | KC250999 | Yes |
|  |  | 4x | Dickinson, T.A.; Talent, N.; Nguyen, S.; Lance, R. | 2003-25 | 19-Apr-2003 | TRT063 | TRT00001866 | AL, Autauga Co. (32.3989, -86.7794) | | | KC861770 |  | KC251215 | KC250997 | Yes |
|  |  | 4x | Talent, N.; Lance, R.W.; Talent, J.G. | NT275 | 19-Apr-2004 | TRT110 | TRT00000049 | AL, Lowndes Co. (32.3278, -86.7583) | | | KC861771 | KC251489 | KC251216 | KC250998 | Yes |
|  |  | 4x | Dickinson, T.A.; Talent, N.; Nguyen, S.; Lance, R. | 2003-24 | 19-Apr-2003 | TRT061 | TRT00000052 | AL, Autauga Co. (32.399, -86.779) | | | KC861772 | KC251470 | KC251195 | KC250978 | Yes |
|  | *C. harbisonii* Beadle | | |  |  |  |  |  | | |  |  |  |  |  |
|  |  | 4x | Lance, R. | 2302 | May-2003 | TRT012 | TRT00000059 | TN, Davidson Co. (36.07, -86.88) | | | KC861811 | KC251578 | KC251305 | KC251061 | Yes |
|  |  | 4x | Lance, R. | 2303 | May-2003 | TRT017 | TRT00001869 | TN, Davidson Co. (36.07, -86.88) | | | KC861812 | KC251581 | KC251308 | KC251064 | Yes |
|  |  | 4x | Lance, R. | 2304 | May-2003 | TRT024 | TRT00000058 | TN, Davidson Co. (36.07, -86.88) | | | KC861815 | KC251579 | KC251306 | KC251062 | Yes |
|  |  | 4x | Lance, R. | 2307 | May-2003 | TRT022 | TRT00001868 | NC, Buncombe Co. (35.4976, -82.609) | | | KC861813 | KC251580 | KC251307 | KC251063 | Yes |
|  |  | 4x | Lance, R. | 2308 | May-2003 | TRT023 | TRT00000060 | NC, Buncombe Co. (35.4976, -82.609) | | | KC861814 | KC251577 | KC251304 | KC251060 | Yes |
|  | ***Coccineae*** (Loudon) Rehder | | |  |  |  |  |  | | |  |  |  |  |  |
|  | *C. magniflora* Sarg. | | |  |  |  |  |  | | |  |  |  |  |  |
|  |  | NA | Phipps, J.B. | 7976 | 11-May-1999 | MKTRT632 | TRT00007823 | ON, Middlesex Co. (43.0667, -81.3167) | | |  | KP050223 | KP050296 | KP050195 | Yes |
|  |  | NA | Phipps, J.B. | 7118 | 24-May-1995 | MKTRT631 | TRT00007825 | ON, Essex Co. (42.05, -82.8167) | | |  |  | KP050285 |  | Yes |
|  |  | NA | Phipps, J.B. | 4585 | 12-Jun-1975 | MKTRT633 | TRT00007826 | ON, Grey Co. (44.5833, -80.5) | | |  |  | KP050284 |  | Yes |
|  | ***Crus-galli*** (Loudon) Rehder | | |  |  |  |  |  | | |  |  |  |  |  |
|  | *C. berberifolia* Torr. & Gray var. *engelmannii* (Sarg.) Eggleston | | | |  |  |  |  | | |  |  |  |  |  |
|  |  | 4x | Talent, N.; Lance, R.W.; Talent, J.G. | NT274 | 19-Apr-2004 | TRT111 | TRT00019451 | AL, Lowndes Co. (32.328, -86.758) | | | KC861785 | KC251468 | KC251193 | KC250976 | Yes |
|  |  | 4x | Talent, N. | NT276 | 19-Apr-2004 | TRT112 | TRT00019452 | AL, Lowndes Co. (32.328, -86.758) | | | KC861788 | KC251471 | KC251196 | KC250979 | Yes |
|  | *C. crus-galli* L. | |  |  |  |  |  |  | | |  |  |  |  |  |
|  |  | 2x | Talent, N. | NT213a | 18-Apr-1999 | ***TRT198*** | ***TRT00019161*** | AL, Montgomery Co. (32.311, -86.3744) | | | KC861786 | KC251521 |  | KC251022 | No |
|  |  | 3x | Dickinson, T.A. | 2011-1 | 23-Mar-2011 | TRT247 | TRT00034838 | LA, Caldwell Co. (32.0342, -92.0342) | | |  |  | KC251246 | KC251020 | Yes |
|  |  | 4x- | Dickinson, T.A.; Lo, E.; Chung, J. | 2006-07 | 14-Jul-2006 | TRT136 | TRT00000457 | ON, Niagara RM (43.164, -79.0695) | | | KC861784 | KC251520 | KC251247 | KC251021 | Yes |
|  |  | NA | Dickinson, T.A.; Talent, N.; Nguyen, S. | 2002-07 | 20-Apr-2003 | TRT064 | TRT00000530 | MS, Scott Co. (32.3594, -89.5547) | | | KC861783 | KC251522 | KC251248 |  | Yes |
|  |  | 4x | Lance, R.W. | RWL-3562 | 16-Apr-2011 | TRT252 | TRT00021214 | SC, McCormick Co. (33.6815, -82.2021) | | |  | KC251523 | KC251249 | KC251023 | Yes |
|  |  | 4x | Lance, R.W. | RWL-3565 | 16-Apr-2011 | TRT259 | TRT00021215 | SC, McCormick Co. (33.6815, -82.2021) | | | KC861787 | KC251524 | KC251250 | KC251024 | Yes |
|  | *C. persimilis* Sarg. | | |  |  |  |  |  | | |  |  |  |  |  |
|  |  | 4x | Dickinson, T.A.; Lo, E.; Chung, J. | 2006-05 | 14-Jul-2006 | TRT135 | TRT00000723 | ON, Niagara RM (43.1625, -79.0694) | | | KC861855 | KC251640 | KC251365 | KC251114 | Yes |
|  |  | 4x | Dickinson, T.A.; Lo, E.; Chung, J. | 2006-06 | 14-Jul-2006 | TRT137 | TRT00000722 | ON, Niagara RM (43.1625, -79.0694) | | | KC861856 | KC251639 | KC251364 | KC251113 | Yes |
|  |  | 4x | Dickinson, T.A.; Lo, E.; Chung, J. | 2006-08 | 14-Jul-2006 | TRT138 | TRT00000727 | ON, Niagara RM (43.1648, -79.0664) | | | KC861854 | KC251641 | KC251366 | KC251115 | Yes |
|  | *C. tenax* Ashe | | |  |  |  |  |  | | |  |  |  |  |  |
|  |  | 4x | Dickinson, T.A.; Talent, N.; Nguyen, S. | 2003-28 | 20-Apr-2003 | TRT142 | TRT00000803 | MS, Scott Co. (32.3, -89.267) | | |  | KC251467 | KC251192 | KC250975 | Yes |
|  | ***Dilatatae*** (Sarg.) Rehder | | |  |  |  |  |  | | |  |  |  |  |  |
|  | *C. coccinioides* Ashe | | |  |  |  |  |  | | |  |  |  |  |  |
|  |  | NA | Phipps, J.B. | 4497 | 21-May-1975 | MKTRT636 | TRT00007260 | ON, Niagara R.M. (43.1333, -79.25) | | |  |  | KP050255 |  | Yes |
|  |  | NA | Phipps, J.B. | 8006 | 15-Sep-1999 | MKTRT634 | TRT00007262 | ON, Essex Co. (42.0667, -82.8667) | | |  |  | KP050270 |  | Yes |
|  |  | NA | Sinnott, Q.P. | 815 | 18-May-1977 | MKTRT635 | TRT00007258 | ON, Niagara R.M. (43.1667, -79.0833) | | |  |  | KP050244 | KP050164 | Yes |
|  | ***Intricatae*** (Sarg.) Rehder | | |  |  |  |  |  | | |  |  |  |  |  |
|  | *C. intricata* Lange | | |  |  |  |  |  | | |  |  |  |  |  |
|  |  | NA | Lance, R.W. | 3251 | 16-Sep-2008 | MKTRT637 | TRT00006903 | NC, Buncombe Co. (35.4976, -82.609) | | | KP050147 |  | KP050286 | KP050189 | Yes |
|  |  | NA | Smith, T.E.; Brant, A. | 4253 | 18-May-2006 | MKTRT639 | TRT00006887 | MO, Dent Co. (37.41, -91.306) | | |  |  | KP050249 | KP050167 | Yes |
|  |  | NA | Smith, T.E. | 3879 | 5-May-2004 | MKTRT638 | TRT00006888 | MO, Texas Co. (37.169, -91.803) | | | KP050141 |  | KP050236 | KP050157 | Yes |
|  | ***Lacrimatae*** J.B. Phipps | | |  |  |  |  |  | | |  |  |  |  |  |
|  | *C. dispar* Beadle | | |  |  |  |  |  | | |  |  |  |  |  |
|  |  | 4x | Lance, R.W. | 3570 | 16-Apr-2011 | TRT251 | TRT00021220 | SC, McCormick Co. (33.6855, -82.1626) | | | KC861789 | KC251529 | KC251255 | KC251028 | Yes |
|  | *C. lassa* Beadle | | |  |  |  |  |  | | |  |  |  |  |  |
|  |  | 4x | Dickinson, T.A.; Talent, N.;Nguyen, S.; Lance, R. | 2003-18 | 19-Apr-2003 | TRT027 | TRT00001962 | AL, Dallas Co. (32.386, -86.971) | | | KC861824 | KC251473 | KC251198 | KC250981 | Yes |
|  |  | 3x | Lance, R.W. | 3560 | 15-Apr-2011 | TRT250 | TRT00021213 | SC, Aiken Co. (33.43, -81.48) | | |  | KC251593 | KC251320 | KC251076 | Yes |
|  |  | NA | Lance, R.W. | 3568 | 16-Apr-2011 | TRT254 | TRT00021219 | SC, McCormick Co. (33.6926, -82.1887) | | | KC861825 |  |  | KC251077 | No |
|  | ***Molles*** (Sarg.) Rehder | | |  |  |  |  |  | | |  |  |  |  |  |
|  | *C. brazoria* Sarg. | | |  |  |  |  |  | | |  |  |  |  |  |
|  |  | NA | Lance, R. W. | 3594 | 22-May-2011 | MKTRT660 | TRT00021228 | TX, Fort Bend Co. (29.368, -95.578) | | |  | KP050216 | KP050272 | KP050182 | Yes |
|  |  | NA | Lance, R.W. | 3598 | 22-May-2011 | MKTRT664 | TRT00021229 | TX, Fort Bend Co. (29.376, -95.577) | | |  | KP050204 |  | KP050159 | Yes |
|  | *C. mollis* (Torrey & A. Gray) Scheele | | |  |  |  |  |  | | |  |  |  |  |  |
|  |  | 3x | Dickinson, T.A.; Talent, N.; Nguyen, S.; Lance, R.W. | 2003-13 | 19-Apr-2003 | TRT052 | TRT00000192 | AL, Lowndes Co. (32.2611, -86.6486) | | |  | KC251607 | KC251333 | KC251088 | Yes |
|  |  | 3x | Dickinson, T.A.; Talent, N.; Nguyen, S.; Lance, R.W. | 2003-16 | 19-Apr-2003 | TRT051 | TRT00000191 | AL, Lowndes Co. (32.2611, -86.6486) | | |  | KC251608 | KC251334 | KC251089 | Yes |
|  |  | NA | Lance, R.W. | 3589 | 22-May-2011 | MKTRT656 | TRT00021237 | TX, Brazoria Co. (29.35, -95.53) | | |  | KP050202 | KP050231 | KP050154 | Yes |
|  |  | 2x | Nguyen, S. | s.n.01 | Aug-2002 | TRT076 | Unvouchered; | WI, Dane Co. (43.0759, -89.397) | | |  | KC251609 | KC251335 | KC251090 | Yes |
|  |  |  |  |  |  |  | sight identification | | | |  |  |  |  |  |
|  | *C. mollis* var. *texana* (Buckley) R.W. Lance | | |  |  |  |  |  | | |  |  |  |  |  |
|  |  | NA | Lance, R.W. | 3590 | 22-May-2011 | MKTRT657 | TRT00021233 | TX, Brazoria Co. (29.35, -95.53) | | |  |  | KP050299 | KP050197 | Yes |
|  |  | NA | Lance, R.W. | 3596 | 22-May-2011 | MKTRT662 | TRT00021234 | TX, Fort Bend Co. (29.376, -95.577) | | |  | KP050206 | KP050239 | KP050161 | Yes |
|  |  | NA | Lance, R.W. | 3597 | 22-May-2011 | MKTRT663 | TRT00021232 | TX, Fort Bend Co. (29.376, -95.577) | | |  | KP050214 |  | KP050178 | Yes |
|  | *C. mollis* var. *viburnifolia* (Sarg.) R.W. Lance | | |  |  |  |  |  | | |  |  |  |  |  |
|  |  | NA | Lance, R. W. | 3593 | 22-May-2011 | MKTRT659 | TRT00021238 | TX, Fort Bend Co. (29.368, -95.578) | | |  | KP050215 | KP050271 | KP050181 | Yes |
|  | ***Molles*** × ***Virides*** hybrid | | |  |  |  |  |  | | |  |  |  |  |  |
|  | *C. mollis* × *C. viridis* | | |  |  |  |  |  | | |  |  |  |  |  |
|  |  | NA | Lance, R.W. | 3599 | 22-May-2011 | MKTRT665 | TRT00021230 | TX, Fort Bend Co. (29.376, -95.577) | | |  | KP050225 | KP050302 | KP050200 | Yes |
|  |  | NA | Lance, R. W. | 3592 | 22-May-2011 | MKTRT658 | TRT00021239 | TX, Fort Bend Co. (29.368, -95.578) | | | KP050142 | KP050205 | KP050238 | KP050160 | Yes |
|  |  | NA | Lance, R.W. | 3595 | 22-May-2011 | MKTRT661 | TRT00021231 | TX, Fort Bend Co. (29.368, -95.578) | | |  | KP050218 | KP050279 | KP050186 | Yes |
|  | ***Parvifoliae*** (Loudon) Rehder | | |  |  |  |  |  | | |  |  |  |  |  |
|  | *C. uniflora* Münchhausen | | |  |  |  |  |  | | |  |  |  |  |  |
|  |  | 4x | Dickinson, T.A. | 2002-03A | 10-Sep-1999 | TRT123 | TRT00001980 | MA, Suffolk Co. (42.3031, -71.1242) | | | KC861905 | KC251718 | KC251443 | KC251170 | Yes |
|  |  | 4x | Dickinson, T.A. | 2002-03A | 20-Jun-2002 | TRT124 | TRT00001981 | MA, Suffolk Co. (42.2939, -71.1265) | | | KC861906 | KC251714 | KC251439 | KC251166 | Yes |
|  |  | 3x | Dickinson, T.A.; Talent, N.; Nguyen, S. | 2003-49 | 25-Apr-2003 | TRT188 | TRT00001985 | VA, Franklin Co. (37.0061, -79.9189) | | |  | KC251715 | KC251440 | KC251167 | Yes |
|  |  | 3x | Dickinson, T.A.; Talent, N.; Nguyen, S. | 2003-51 | 25-Apr-2003 | TRT187 | TRT00001984 | VA, Franklin Co. (37.0061, -79.9189) | | |  | KC251717 | KC251442 | KC251169 | Yes |
|  |  | 3x | Dickinson, T.A.; Talent, N.; Nguyen, S. | 2003-52 | 25-Apr-2003 | TRT189 | TRT00001986 | VA, Franklin Co. (37.0061, -79.9189) | | |  | KC251719 | KC251444 | KC251171 | Yes |
|  |  | 3x | Dickinson, T.A.; Talent, N.; Nguyen, S.; Lance, R. | 2003-26 | 19-Apr-2003 | TRT186 | TRT00001983 | AL, Autauga Co. (32.3989, -86.7794) | | |  | KC251716 | KC251441 | KC251168 | Yes |
|  | ***Populneae*** J.B. Phipps | | |  |  |  |  |  | | |  |  |  |  |  |
|  | *C. aemula* Beadle | | |  |  |  |  |  | | |  |  |  |  |  |
|  |  | 4x | Lance, R.W. | 3566 | 16-Apr-2011 | TRT257 | TRT00021216 | SC, McCormick Co. (33.7059, -82.1816) | | | KC861765 | KC251483 | KC251208 | KC250990 | Yes |
|  |  | NA | Lance, R.W. | 3567 | 16-Apr-2011 | TRT256 | TRT00021218 | SC, McCormick Co. (33.6998, -82.1746) | | | KC861766 | KC251482 | KC251207 | KC250989 | Yes |
|  |  | NA | Lance, R.W. (seedling 1) | 2311 | May-2003 | TRT013 | TRT00020700 | GA, Walker Co. (34.6862, -85.3851) | | | KC861762 | KC251477 | KC251202 | KC250985 | Yes |
|  |  | 4x | Lance, R.W. (seedling 2) | 2311 | May-2003 | TRT014 | TRT00020701 | GA, Walker Co. (34.6862, -85.3851) | | | KC861759 | KC251478 | KC251203 | KC250986 | Yes |
|  |  | 4x | Lance, R.W. (seedling 3) | 2311 | May-2003 | TRT015 | TRT00020702 | GA, Walker Co. (34.6862, -85.3851) | | | KC861764 | KC251476 | KC251201 | KC250984 | Yes |
|  |  | 4x | Lance, R.W. (seedling 4) | 2311 | May-2003 | TRT016 | TRT00020703 | GA, Walker Co. (34.6862, -85.3851) | | | KC861760 | KC251479 | KC251204 | KC250987 | Yes |
|  |  | 4x | Lance, R.W. (seedling 5) | 2311 | May-2003 | TRT019 | TRT00020704 | GA, Walker Co. (34.6862, -85.3851) | | | KC861763 | KC251481 | KC251206 | KC250988 | Yes |
|  |  | 4x | Lance, R.W. (seedling 6) | 2311 | May-2003 | TRT011 | TRT00020705 | GA, Walker Co. (34.6862, -85.3851) | | | KC861761 | KC251480 | KC251205 |  | Yes |
|  | *C. beata* Sarg. | | |  |  |  |  |  | | |  |  |  |  |  |
|  |  | NA | Phipps, J.B. | 7983 | 16-May-1999 | MKTRT624 | TRT00008753 | ON, Essex Co. (42.05, -82.8167) | | |  |  | KP050246 | KP050165 | Yes |
|  |  | NA | Phipps, J.B. | 5049 | 30-May-1978 | MKTRT622 | TRT00008754 | ON, Middlesex Co. (43.0667, -81.8) | | |  |  | KP050300 | KP050198 | Yes |
|  |  | NA | Phipps, J.B. | 7973 | 7-May-1999 | MKTRT623 | TRT00008755 | ON, Essex Co. (42.05, -82.8167) | | | KP050149 | KP050222 | KP050294 | KP050194 | Yes |
|  | ***Pruinosae*** (Sarg.) Rehder | | |  |  |  |  |  | | |  |  |  |  |  |
|  | *C. compacta* Sarg. | | |  |  |  |  |  | | |  |  |  |  |  |
|  |  | NA | Barrows, D. | 18 | 26-May-1977 | MKTRT601 | TRT00006752 | ON, Middlesex Co. (42.7167, -81.6667) | | |  |  | KP050250 |  | Yes |
|  |  | NA | Phipps, J.B. | 4450 | 4-Jun-1974 | MKTRT602 | TRT00006742 | ON, Kent Co. (42.65, -81.8833) | | |  |  | KP050241 |  | Yes |
|  |  | NA | Phipps, J.B. | 4512 | 22-May1975 | MKTRT603 | TRT00006745 | ON, Lambton Co. (42.7833, -82.3167) | | |  |  | KP050274 |  | Yes |
|  | *C. formosa* Sarg. | | |  |  |  |  |  | | |  |  |  |  |  |
|  |  | NA | Davies, J. | 77-9 | 18-May-1977 | MKTRT606 | TRT00006699 | ON, Halton R.M. (43.291, -79.875) | | |  |  | KP050243 | KP050163 | Yes |
|  |  | NA | Phipps, J.B. | 5031 | 25-Sep-1977 | MKTRT604 | TRT00006697 | ON, Halton R.M. (43.2833, -79.8833) | | |  |  | KP050276 | KP050184 | Yes |
|  |  | NA | Phipps, J.B. | 5071 | 2-Jun-1978 | MKTRT605 | TRT00006698 | ON, Halton R.M. (43.2833, -79.8833) | | | KP050144 |  | KP050263 | KP050176 | Yes |
|  | *C. perjucunda* Sarg. | | |  |  |  |  |  | | |  |  |  |  |  |
|  |  | NA | Sinnott, Q.P. | 969 | 1-Oct-1977 | MKTRT607 | TRT00006691 | ON, Middlesex Co. (42.95, -81.2167) | | |  |  | KP050264 |  | Yes |
|  |  | NA | Sinnott, Q.P. | 780 | Oct-1976 | MKTRT608 | TRT00006693 | ON, Middlesex Co. (42.95, -81.2167) | | |  |  | KP050277 |  | Yes |
|  |  | NA | Sinnott, Q.P. | 940 | 20-Sep-1977 | MKTRT609 | TRT00006695 | ON, Middlesex Co. (42.95, -81.2167) | | |  |  | KP050287 | KP050190 | Yes |
|  | ***Pulcherrimae*** (Beadle) K.R. Robertson | | |  |  |  |  |  | | |  |  |  |  |  |
|  | C. robur | | |  |  |  |  |  | | |  |  |  |  |  |
|  |  | 3x | Dickinson, T.A.; Talent, N.; Nguyen, S.; Lance, R. | 2003-10 | 19-Apr-2003 | TRT026 | TRT00000231 | AL, Montgomery Co. (32.31, -86.375) | | | KC861877 | KC251472 | KC251197 | KC250980 | Yes |
|  | C. agrestina | | |  |  |  |  |  | | |  |  |  |  |  |
|  |  | 4x | Lance, R. | 2003-20 | Apr-2003 | TRT021 | TRT00002406 | AL, Lowndes Co. (32.333, -86.675) | | |  | KC251484 | KC251209 | KC250991 | Yes |
|  | ***Punctatae*** (Loudon) Rehder | | |  |  |  |  |  | | |  |  |  |  |  |
|  | *C. jonesiae* Sarg. | | |  |  |  |  |  | | |  |  |  |  |  |
|  |  | NA | Hinds, H. | 953 | 22-Aug-1978 | MKTRT610 | TRT00006288 | NB, Charlotte Co. (45.098, -67.05) | | |  |  | KP050289 |  | Yes |
|  |  | NA | Phipps, J.B. | 9025 | 16-Sep-2005 | MKTRT612 | TRT00006286 | ME, Kennebec Co. (44.1167, -70.0917) | | |  |  | KP050237 | KP050158 | Yes |
|  |  | NA | Rossbach, G.B. | 2573 | 8-Jun-1953 | MKTRT611 | TRT00006287 | ME, Knox Co. (44.19, -69.067) | | |  |  |  |  | No |
|  | *C. grandis* Ashe | | |  |  |  |  |  | | |  |  |  |  |  |
|  |  | (3x) | Dickinson, T.A.; Lo, E.; Chung, J. | 2006-10 | 14-Jul-2006 | TRT144 | TRT00000864 | ON, Niagara RM (43.247, -79.06) | | | KC861810 | KC251465 | KC251190 | KC250973 | Yes |
|  |  | (3x) | Dickinson, T.A.; Lo, E.; Chung, J. | 2006-11 | 14-Jul-2006 | TRT134 | TRT00000862 | ON, Niagara RM (43.247, -79.06) | | | KC861809 | KC251469 | KC251194 | KC250977 | Yes |
|  | *C. mexicana* Moc. & Sessé | | |  |  |  |  |  | | |  |  |  |  |  |
|  |  | NA | Harris, E. | s.n. | 22-Jul-1998 | TRT049 |  | CA, Alameda Co. U. of California Botanical Garden (37.875, -122.239)  76-2049 | | | KC861860 | KC251646 | KC251371 |  | Yes |
|  | *C. punctata* Jacq. | | |  |  |  |  |  | | |  |  |  |  |  |
|  |  | (2x) | Dickinson, T.A.; Shaik, M. | 2008-72A | 21-Jun-2008 | TRT210 | TRT00000908 | ON, Perth Co. (43.313, -81.17) | | | KC861867 | KC251648 | KC251373 | KC251121 | Yes |
|  |  | (2x) | Purich, M.A.; McGrath, N. | 056 | 10-May-2004 | TRT105 | TRT00002223 | ON, Middlesex Co. (43.0726, -81.4019) | | | KC861861 | KC251647 | KC251372 | KC251120 | Yes |
|  |  | (2x) | Purich, M.A.; McGrath, N. | 061 | 10-May-2004 | TRT096 | TRT00002228 | ON, Middlesex Co. (43.0726, -81.4019) | | | KC861862 | KC251650 | KC251375 | KC251123 | Yes |
|  |  | (2x) | Purich, M.A.; Purich, A. | 035 | 19-May-2004 | TRT101 | TRT00002203 | ON, City of Toronto (43.6581, -79.3071) | | | KC861865 | KC251649 | KC251374 | KC251122 | Yes |
|  |  | (2x) | Purich, M.A.; Talent, N. | 081 | 03-Jun-2004 | TRT104 | TRT00002247 | ON, Durham R.M. (43.9019, -78.6829) | | | KC861864 | KC251653 | KC251378 | KC251126 | Yes |
|  |  | (2x) | Purich, M.A.; Talent, N., Nguyen, S.; Lo, E. | 024 | 18-May-2004 | TRT106 | TRT00002199 | ON, City of Toronto (43.6483, -79.5947) | | | KC861866 | KC251651 | KC251376 | KC251124 | Yes |
|  |  | (2x) | Purich, M.A.; Talent, N.; Nguyen, S.; Lo, E. | 071 | 18-May-2004 | TRT103 | TRT00002237 | ON, City of Toronto (43.6483, -79.5947) | | | KC861863 | KC251652 | KC251377 | KC251125 | Yes |
|  | ***Rotundifoliae*** (Eggleston) Rehder | | |  |  |  |  |  | | |  |  |  |  |  |
|  | *C. sheila-phippsiae* J.B. Phipps & O’Kennon | | |  |  |  |  |  | | |  |  |  |  |  |
|  |  | NA | Jackson, R. (for Phipps, J.B.) | 8407 | 23-May-2002 | MKTRT617 | TRT00009434 | BC, North Okanagan R.D. (50.4736, -119.152) | | |  |  | KP050259 | KP050174 | Yes |
|  |  | NA | Phipps, J.B. | 8332 | 16-May-2002 | MKTRT620 | TRT00009435 | BC, North Okanagan R.D. (50.43, -119.235) | | |  | KP050220 | KP050283 | KP050188 | Yes |
|  |  | NA | Phipps, J.B. | 6917 | 5-May-1994 | MKTRT616 | TRT00009438 | BC, North Okanagan R.D. (50.55, -119.112) | | |  |  | KP050301 | KP050199 | Yes |
|  | *C. chrysocarpa* Ashe | | |  |  |  |  |  | | |  |  |  |  |  |
|  |  | 4x | Dickinson, T.A.; Coughlan, J.; Zarrei, M. | NT587 | 26-Aug-2010 | TRT238 | TRT00002699 | BC, Central Kootenay R.D. (49.8043, -118.154) | | |  | KC251514 | KC251241 | KC251014 | Yes |
|  |  | 4x | Talent, N.; Heckel, M. | NT544 | 10-May-2010 | TRT237 | TRT00021118 | BC, North Okanagan R.D. (50.4811, -119.116) | | | KC861781 | KC251515 | KC251242 | KC251015 | Yes |
|  |  | 4x | Talent, N.; Lee, J. | 2007-08 | 28-May-2008 | TRT214 | TRT00002694 | BC, Central Kootenay R.D. (49.8067, -118.158) | | |  | KC251517 | KC251244 | KC251017 | Yes |
|  | *C. chrysocarpa* var. *piperi* (Britton) Kruschke | | |  |  |  |  |  | | |  |  |  |  |  |
|  |  | 4x | Coughlan, J.; Zarrei, M.; Shaw, C. | JC308 | 31-May-2011 | TRT273 | TRT00020174 | MT, Flathead Co. (48.1919, -114.121) | | | KC861782 | KC251519 | KC251245 | KC251019 | Yes |
|  |  | 5x | Coughlan, J.; Zarrei, M.; Shaw, C. | JC310 | 31-May-2011 | TRT278 | TRT00020175 | MT, Flathead Co. (48.1895, -114.122) | | |  | KC251518 |  | KC251018 | No |
|  |  | 4x | Love, R.M. | s.n.  Sample 1 | 00-May-2003 | TRT048 | TRT00018677? | WA, Klickitat Co. (45.6495, -121.116) | | |  | KC251516 | KC251243 | KC251016 | Yes |
|  |  | 4x+ | Love, R.M. | s.n.  Sample 2 | 00-May-2003 | ***TRT047*** | ***TRT00018678*** | WA, Klickitat Co. (45.6495, -121.116) | | |  | KC251513 | KC251240 | KC251013 | Yes |
|  | *C. dodgei* Ashe | | |  |  |  |  |  | | |  |  |  |  |  |
|  |  | 4x | Lo, E.; Talent, N. | EL2 | 1-Jun-2004 | TRT183 | TRT00001861 | ON, Grey Co. (44.3103, -80.4831) | | | KC861790 | KC251530 | KC251256 | KC251029 | Yes |
|  | *C. flavida* Sarg. | | |  |  |  |  |  | | |  |  |  |  |  |
|  |  | 4x+ | Dickinson, T.A. | 2000-68 | 20-May-2000 | TRT039 | TRT00000066 | MA, Suffolk Co. Arnold Arboretum (42.3022, -71.1178) AA220-89A | | | KC861797 | KC251561 | KC251287 | KC251053 | Yes |
|  | *C. irrasa* Sarg. | | |  |  |  |  |  | | |  |  |  |  |  |
|  |  | NA | Draper, W.B. | 93-33 | 27-May-1993 | MKTRT619 | TRT00009402 | ON, Muskoka D. M. (45.05, -79.3) | | |  |  | KP050253 | KP050170 | Yes |
|  |  | NA | Draper, W.B. | 92-6 | 1-Sep-1992 | MKTRT621 | TRT00009403 | ON, Muskoka D. M. (45.037, -79.284) | | |  |  | KP050257 |  | Yes |
|  |  | NA | Rouleau, E. | 9384 | 8-Sep-1963 | MKTRT618 | TRT00009406 | NL, St. Georges Dist. (48.17, -58.77) | | |  |  | KP050230 | KP050153 | Yes |
|  | ***Tenuifoliae*** (Sarg.) Rehder | | |  |  |  |  |  | | |  |  |  |  |  |
|  | *C. iracunda* Beadle | | |  |  |  |  |  | | |  |  |  |  |  |
|  |  | 3x | Lance, R.W.; | RWL-3563a | 16-Apr-2011 | TRT262 | TRT00021246 | SC, McCormick Co. (33.6815, -82.2021) | | | KC861818 | KC251588 | KC251315 | KC251071 | Yes |
|  |  | 3x | Lance, R.W.; | RWL-3569 | 16-Apr-2011 | TRT255 | TRT00021247 | SC, McCormick Co. (33.6926, -82.1887) | | | KC861819 | KC251587 | KC251314 | KC251070 | Yes |
|  | *C. fluviatilis* Sarg. | | |  |  |  |  |  | | |  |  |  |  |  |
|  |  | NA | Phipps, J.B. | 5072 | 2-Jun-1978 | MKTRT625 | TRT00008504 | ON, Halton R.M. (48.2833, -79.8833) | | | KP050140 |  | KP050233 |  | Yes |
|  |  | NA | Phipps, J.B. | 4878 | 20-May-1977 | MKTRT626 | TRT00008509 | ON, Grey Co. (46.3, -80.5167) | | |  |  | KP050295 |  | Yes |
|  |  | NA | Ulf-Hansen, P.F.; Smith, P.G. | PF092 | 3-Jun-1983 | MKTRT627 | TRT00008510 | ON, Middlesex Co. (43.1167, -81.8) | | |  |  |  |  | No |
|  | *C. schuettei* Ashe | | |  |  |  |  |  | | |  |  |  |  |  |
|  |  | NA | Phipps, J.B. | 5045 | 29-May-1978 | MKTRT629 | TRT00008424 | ON, Middlesex Co. (42.95, -81.2167) | | |  |  | KP050245 |  | Yes |
|  |  | NA | Phipps, J.B. | 7728 | 5-May-1998 | MKTRT628 | TRT00008428 | ON, Essex Co. (42.05, -82.8167) | | | KP050146 |  | KP050280 |  | Yes |
|  |  | NA | Ulf-Hansen, P.F. | PF065 | 23-May-1983 | MKTRT630 | TRT00008422 | ON, Middlesex Co. (42.7278, -81.6667) | | | KP050145 |  | KP050267 |  | Yes |
|  | *C. turnerorum* Enquist | | |  |  |  |  |  | | |  |  |  |  |  |
|  |  | 4x | Dickinson, T.A. | s.n. | 16-May-1999 | TRT040 | Unvouchered; Arboretum identification | MA, Suffolk Co. Arnold Arboretum (42.298, -71.128) AA235-89 | | | KC861904 | KC251474 | KC251199 | KC250982 | Yes |
|  | ***Triflorae*** (Beadle) Rehder | | |  |  |  |  |  | | |  |  |  |  |  |
|  | *C. triflora* Chapman | | |  |  |  |  |  | | |  |  |  |  |  |
|  |  | 3x | Dickinson, T.A.; Talent, N.; Nguyen, S.; Lance, R. | 2003-07 | 19-Apr-2003 | TRT115 | TRT00021428 | AL, Montgomery Co. (32.3097, -86.375) | | | KC861900 | KC251710 | KC251435 | KC251162 | Yes |
|  |  | 4x | Dickinson, T.A.; Talent, N.; Nguyen, S.; Lance, R. | 2003-09 | 19-Apr-2003 | TRT116 | TRT00021430 | AL, Montgomery Co. (32.3097, -86.375) | | | KC861901 | KC251713 | KC251438 | KC251165 | Yes |
|  |  | 2x | Dickinson, T.A.; Talent, N.; Nguyen, S.; Lance, R. | 2003-22 | 19-Apr-2003 | TRT102 | TRT00021431 | AL, Autauga Co. (32.3989, -86.7794) | | | KC861903 | KC251711 | KC251436 | KC251163 | Yes |
|  |  | 2x | Dickinson, T.A.; Talent, N.; Nguyen, S.; Lance, R. | 2003-23 | 19-Apr-2003 | TRT107 | TRT00021429 | AL, Autauga Co. (32.3989, -86.7794) | | | KC861902 | KC251712 | KC251437 | KC251164 | Yes |
|  | ***Virides*** (Gordon) Rehder | | |  |  |  |  |  | | |  |  |  |  |  |
|  | *C. viridis* L. | | |  |  |  |  |  | | |  |  |  |  |  |
|  |  | NA | Dickinson, T.A. | s.n. | 21-May-2000 | TRT056 | TRT00002078 | MA, Suffolk Co. (42.3031, -71.1242) | | | KC861908 | KC251722 | KC251447 | KC251174 | Yes |
|  |  | 2x | Dickinson, T.A.; Talent, N.; Nguyen, S. | 2003-44 | 23-Apr-2003 | TRT065 | TRT00002075 | AR, Prairie Co. (34.6322, -91.5917) | | | KC861907 | KC251721 | KC251446 | KC251173 | Yes |
|  |  | 2x | Dickinson, T.A.; Talent, N.; Nguyen, S. | 2003-45 | 23-Apr-2003 | TRT130 | TRT00002076 | AR, Prairie Co. (34.6322, -91.5917) | | | KC861909 | KC251720 | KC251445 | KC251172 | Yes |
| D. ***Macracanthae*** Loudon | | | |  |  |  |  |  | | |  |  |  |  |  |
|  | ***Anomalae*** (Sarg. Ex Eggleston) Rehder | | |  |  |  |  |  | | |  |  |  |  |  |
|  | *C. florifera* Sarg. | | |  |  |  |  |  | | |  |  |  |  |  |
|  |  | NA | Barrows, D. | B50 | 01-Jun-1977 | MKTRT640 | TRT00010425 | ON, Elgin Co. (42.5, -81.6167) | | |  |  | KP050262 |  | Yes |
|  |  | NA | Phipps, J.B. | 7736 | 11-May-1998 | MKTRT642 | TRT00010418 | ON, Essex Co. (42.05, -82.8167) | | |  |  | KP050240 |  | Yes |
|  |  | NA | Phipps, J.B.; Dvosky, K. | 8917 | 30-May-2005 | MKTRT641 | TRT00010419 | ON, Middlesex Co. (43, -81.255) | | | KP050151 |  | KP050303 |  | Yes |
|  | C. scabrida Sarg. | | |  |  |  |  |  | | |  |  |  |  |  |
|  |  | NA | Harris, E. | EH-78 | 21-May-2001 | MKTRT643 | TRT00010406 | ON, Haliburton Co. (44.95, -78.65) | | | KP050143 |  | KP050261 |  | Yes |
|  |  | NA | Phipps, J.B.; Smith, P.G. | 5396 | 03-Sep-1984 | MKTRT645 | TRT00010363 | ON, Bruce Co. (45.15, -81.45) | | | KP050148 |  | KP050288 |  | Yes |
|  |  | NA | Smith, P.G. | PS105 | 20-May-1979 | MKTRT644 | TRT00010365 | ON, Middlesex Co. (43.1167, -81.8) | | |  |  | KP050297 |  | Yes |
|  | ***Macracanthae*** (Loudon) Rehder | | |  |  |  |  |  | | |  |  |  |  |  |
|  | *C. calpodendron* (Ehrhart) Medikus | | |  |  |  |  |  | | |  |  |  |  |  |
|  |  | (2x) | Dickinson, T.A.; Han, S.; Buck, K. | 2012-01 | 28-May-2012 | MKTRT655 | TRT00021153 | ON, Middlesex Co. (42.769, -81.818) | | |  | KP050211 | KP050256 |  | Yes |
|  |  | (2x) | Phipps, J.B. | 4870 | 18-May-1977 | MKTRT654 | TRT00009511 | ON, Prince Edward Co. (43.85, -77.1167) | | | KP050150 | KP050224 | KP050298 |  | Yes |
|  |  | (2x) | Phipps, J.B. | 4714 | 10-Jun-1976 | MKTRT653 | TRT00009512 | ON, Lambton Co. (42.75, -81.8667) | | |  |  | KP050269 |  | Yes |
|  |  | (2x) | Phipps, J.B. | 4608 | 15-Jun-1976 | MKTRT652 | TRT00009513 | ON, Middlesex Co. (43.0833, -81.8167) | | |  |  | KP050291 |  | Yes |
|  | *C. macracantha* Lodd. ex Loudon | | |  |  |  |  |  | | |  |  |  |  |  |
|  |  | NA | Dickinson, T.A.; Gervais, G.Y.F.; Dickinson, J.S. | 2001-17 | 31-May-2001 | TRT031 | TRT00000147 | MT, Lake Co. (47.7183, -114.202) | | | KC861826 | KC251597 | KC251324 | KC251081 | Yes |
|  |  | NA | Dickinson, T.A.; Gervais, G.Y.F.; Dickinson, J.S. | 2001-16 | 31-May-2001 | TRT033 | TRT00000146 | MT, Lake Co. (47.7183, -114.202) | | |  | KC251595 | KC251322 | KC251079 | Yes |
|  |  | NA | Dickinson, T.A.; Gervais, G.Y.F.; Dickinson, J.S. | 2001-22 | 31-May-2001 | TRT032 | TRT00000148 | MT, Lake Co. (47.7183, -114.202) | | | KC861828 | KC251596 | KC251323 | KC251080 | Yes |
|  |  | 4x | Talent, N. | NT224 | 04-Jun-2003 | ***TRT127*** | ***TRT00018679*** | ON, City of Toronto (43.6480, -79.5942) | | | KC861827 | KC251598 | KC251325 | KC251082 | Yes |
|  |  | 4x | Talent, N.; Hirst, G. | NT440 | 5-Jun-2009 | TRT234 | TRT00018268 | CO, Boulder Co. (39.9997, -105.215) | | |  | KC251594 | KC251321 | KC251078 | Yes |
|  |  | 4x | Coughlan, J.; Zarrei, M.; Shaw, C. | JC166 | 22-May-2011 | TRT279 | TRT00020257 | WA, Okanogan Co. (48.4999, -119.497) | | | KC861829 | KC251600 | KC251326 | KC251084 | Yes |
|  |  | 4x | Coughlan, J.; Zarrei, M.; Shaw, C. | JC168 | 22-May-2011 | TRT280 | TRT00020260 | WA, Okanogan Co. (48.5002, -119.497) | | | KC861830 | KC251599 |  | KC251083 | No |
|  | *C. rubribracteolata* J.B. Phipps & O’Kennon | | |  |  |  |  |  | | |  |  |  |  |  |
|  |  | NA | Phipps, J.B. | 8673 | 26-May-2004 | MKTRT646 | TRT00010306 | SK, White Valley R.M. (49.567, -109.218) | | |  | KP050209 | KP050252 | KP050169 | Yes |
|  |  | NA | Phipps, J.B. | 8663 | 25-May-2004 | MKTRT648 | TRT00010315 | SK, Piapot R.M. (49.8692, -109.031) | | |  | KP050221 | KP050293 | KP050193 | Yes |
|  |  | NA | Phipps, J.B. | 8650 | 22-May-2004 | MKTRT647 | TRT00010317 | SK, Maple Creek R.M. (49.7336, -109.467) | | |  | KP050217 | KP050278 | KP050185 | Yes |
|  | *C. sheridana* A. Nelson | | |  |  |  |  |  | | |  |  |  |  |  |
|  |  | NA | Phipps, J.B.; O’Kennon, R.J. | 8718 | 31-May-2004 | MKTRT651 | TRT00010191 | AB, Cypress Co. (49.97, -110.05) | | | KP050139 | KP050203 | KP050232 | KP050155 | Yes |
|  |  | NA | Phipps, J.B. ; O’Kennon, R.J. | 8745 | 02-Jun-2004 | MKTRT650 | TRT00010194 | SK, Maple Creek R.M. (49.8, -109) | | |  | KP050210 | KP050254 | KP050171 | Yes |
|  |  | NA | Phipps, J.B. | 9366 | 10-Sep-2008 | MKTRT649 | TRT00010208 | MT, Hill Co. (48.2867, -109.635) | | |  |  | KP050242 | KP050162 | Yes |
|  | | | |  |  |  |  |  | | |  |  |  |  |  |
| E. ***Crataeglasia*** K.I. Chr. & T.A. Dickinson | | | |  |  |  |  |  | | |  |  |  |  |  |
|  | ***Crataeglasianae*** K.I. Chr. & T.A. Dickinson | | |  |  |  |  |  | | |  |  |  |  |  |
|  | *C.* × *cogswellii* K.I. Chr. & T.A. Dickinson | | |  |  |  |  |  | | |  |  |  |  |  |
|  |  | (2x) | Lo, E.; Dickinson, T.A.; Nguyen, S. | EL-71 | 10-Jun-2004 | TRT206 | TRT00002650 | OR, Linn Co. (44.333, -123.122) | | |  | KC251627 |  | KC251106 | No |
|  |  | (2x) | Lo, E.; Dickinson, T.A.; Nguyen, S. | EL-79 | 10-Jun-2004 | ***TRT208*** | ***TRT00002657*** | OR, Linn Co. (44.333, -123.122) | | | KC861847 |  | KC251352 |  | Yes |
|  |  | (2x) | Lo, E.; Dickinson, T.A.; Nguyen, S. | EL-85 | 10-Jun-2004 | ***TRT207*** | ***TRT00002654*** | OR, Linn Co. (44.333, -123.122) | | | KC861846 | KC251626 |  | KC251105 | No |
| E. ***Douglasia*** Loudon | | | |  |  |  |  |  | | |  |  |  |  |  |
|  | ***Cerrones*** J.B. Phipps | | |  |  |  |  |  | | |  |  |  |  |  |
|  | *C. erythropoda* Ashe | | |  |  |  |  |  | | |  |  |  |  |  |
|  |  | 4x | Talent, N. | NT349 | 15-May-2006 | ***TRT241*** | ***TRT00002377*** | CO, Boulder Co. (39.9982, -105.292) | | | KC861795 | KC251558 | KC251284 | KC251050 | Yes |
|  |  | 4x | Talent, N.; Hirst, G. | NT358 | 24-May-2006 | TRT242 | TRT00002387 | NM, Rio Arriba Co. (36.875, -106.583) | | | KC861796 | KC251559 | KC251285 | KC251051 | Yes |
|  | *C. rivularis* Nutt. | | |  |  |  |  |  | | |  |  |  |  |  |
|  |  | 4x | Dickinson, T.A.; Dickinson, A.K. | 2007-01 | 2-Jun-2007 | TRT158 | TRT00000966 | NV, Elko Co. (41.0094, -115.265) | | |  | KC251656 | KC251382 |  | Yes |
|  |  | 4x | Dickinson, T.A.; Dickinson, A.K. | 2007-02 | 2-Jun-2007 | TRT165 | TRT00000965 | NV, Elko Co. (41.0094, -115.265) | | | KC861872 | KC251654 | KC251380 | KC251127 | Yes |
|  |  | 4x | Dickinson, T.A.; Dickinson, A.K. | 2007-03 | 2-Jun-2007 | TRT164 | TRT00000967 | NV, Elko Co. (40.758, -115.437) | | | KC861873 | KC251655 | KC251381 | KC251128 | Yes |
|  |  | 4x | Dickinson, T.A.; Gervais, G.Y.F.; Dickinson, J.S. | 2001-42 | 4-Jun-2001 | ***TRT035*** | ***TRT00001005*** | WY, Converse Co. (42.8397, -105.741) | | | KC861875 | KC251661 | KC251388 |  | Yes |
|  |  | 4x | Dickinson, T.A.; Gervais, G.Y.F., Dickinson, J.S. | 2001-43 | 4-Jun-2001 | TRT121 | TRT00001006 | WY, Converse Co. (42.8397, -105.741) | | | KC861868 | KC251660 | KC251387 |  | Yes |
|  |  | (4x) | Dickinson, T.A.; Gervais, G.Y.F.; Dickinson, J.S. | 2001-05 | 27-May-2001 | TRT212 | TRT00000997 | UT, Uintah Co. (40.4039, -109.916) | | |  |  | KC251379 |  | Yes |
|  |  | 4x | Dickinson, T.A.; Gervais, G.Y.F.; Dickinson, J.S. | 2001-06 | 27-May-2001 | TRT213 | TRT00000995 | UT, Uintah Co. (40.4039, -109.916) | | | KC861871 | KC251662 | KC251389 | KC251131 | Yes |
|  |  | (4x) | Lo, E.; Dickinson, T.A.; Nguyen, S. | EL-199 | 17-Jun-2004 | TRT181 | TRT00000946 | ID, Bear Lake Co. (42.3444, -111.206) | | |  |  | KC251384 |  | Yes |
|  |  | 4x | Lo, E.; Dickinson, T.A.; Nguyen, S. | EL-207 | 17-Jun-2004 | TRT182 | TRT00000945 | ID, Bear Lake Co. (42.3208, -111.262) | | | KC861874 | KC251657 | KC251383 |  | Yes |
|  |  | 4x | Talent, N.; Hirst, G. | NT373 | 14-Jun-2006 | ***TRT235*** | ***TRT00000938*** | CO, Montrose Co. (38.4421, -107.555) | | | KC861870 | KC251659 | KC251386 | KC251130 | Yes |
|  |  | 4x | Talent, N.; Hirst, G. | NT376 | 15-Jun-2006 | TRT236 | TRT00000939 | CO, Archuleta Co. (37.2489, -107.441) | | | KC861869 | KC251658 | KC251385 | KC251129 | Yes |
|  | *C. saligna* Greene | | |  |  |  |  |  | | |  |  |  |  |  |
|  |  | 2x | Dickinson, T.A. | 2004-05 | 18-Jun-2004 | ***TRT120*** | ***TRT00001047*** | UT, Duchesne Co. (40.2083, -110.411) | | |  | KC251670 | KC251397 | KC251136 | Yes |
|  |  | 2x | Dickinson, T.A. | 2004-06 | 18-Jun-2004 | TRT119 | TRT00001026 | CO, Rio Blanco Co. (40.0342, -108.13) | | |  | KC251671 | KC251398 | KC251137 | Yes |
|  |  | 2x | Dickinson, T.A. | 2004-08 | 18-Jun-2004 | TRT113 | TRT00001024 | CO, Rio Blanco Co. (40.0344, -107.863) | | |  | KC251666 | KC251393 | KC251134 | Yes |
|  |  | 2x | Talent, N.; Hirst, G. | NT368 | 14-Jun-2006 | TRT131 | TRT00001018 | CO, Gunnison Co. (38.6141, -106.886) | | |  | KC251667 | KC251394 | KC251135 | Yes |
|  |  | 2x | Talent, N.; Hirst, G. | NT369 | 14-Jun-2006 | TRT132 | TRT00001022 | CO, Gunnison Co. (38.6103, -106.885) | | |  | KC251669 | KC251396 |  | Yes |
|  |  | 2x | Talent, N.; Hirst, G. | NT371 | 14-Jun-2006 | TRT125 | TRT00001021 | CO, Gunnison Co. (38.532, -106.95) | | |  | KC251668 | KC251395 |  | Yes |
|  | ***Douglasianae*** Rehder | | |  |  |  |  |  | | |  |  |  |  |  |
|  | *C. castlegarensis* J.B. Phipps & O’Kennon | | |  |  |  |  |  | | |  |  |  |  |  |
|  |  | 3x | Love, R.M. | C-2003-34F | 09-Apr-2003 | ***TRT042*** | ***TRT00001063*** | OR, Grant Co. (44.584, -119.64) | | |  | KC251509 | KC251237 |  | Yes |
|  |  | 4x | Coughlan, J.; Zarrei, M.; Shaw, C. | JC022 | 05-Sep-2011 | TRT276 | TRT00020128 | CA, Shasta Co. (40.9776, -121.559) | | |  | KC251507 | KC251235 | KC251010 | Yes |
|  |  | 4x | Coughlan, J.; Zarrei, M.; Shaw, C. | JC025 | 05-Sep-2011 | TRT277 | TRT00020131 | CA, Shasta Co. (40.9747, -121.556) | | | KC861780 | KC251511 |  | KC251011 | No |
|  |  | 4x | Dickinson, T. A.; Lo, E. | 2006-13 | 27-Jul-2006 | TRT139 | TRT00001052 | CA, Shasta Co. (40.975, -121.556) | | | KC861779 | KC251510 | KC251238 |  | Yes |
|  |  | 4x | Dickinson, T.A.; Dickson, E.E.; Dickinson, A.K. | 2007-15 | 06-Oct-2007 | TRT156 | TRT00004157 | SK, Cypress Hills (49.534, -109.902) | | |  | KC251506 | KC251234 |  | Yes |
|  |  | 4x | Lo, E.; Dickinson, T.A.; Nguyen, S. | EL-192 | 16-Jun-2004 | TRT180 | TRT00001055 | ID, Lemhi Co. (45.3666, -113.952) | | |  | KC251508 | KC251236 |  | Yes |
|  |  | 4x | Love, R.M. | C-2003-23F | 09-Apr-2003 | TRT018 | TRT00001067 | OR, Grant Co. (44.584, -119.64) | | |  | KC251505 | KC251233 |  | Yes |
|  | *C. douglasii* Lindl. | | |  |  |  |  |  | | |  |  |  |  |  |
|  |  | 4x | Coughlan, J.; Zarrei, M.; Shaw, C. | 2010-24 | 06-Oct-2011 | TRT215 | TRT00002598 | BC, Fraser-Fort George R.D. (53.904, -122.742) | | |  | KC251542 |  | KC251040 | No |
|  |  | 4x | Coughlan, J.; Zarrei, M.; Shaw, C. | 2010-32 | 06-Nov-2011 | TRT218 | TRT00002606 | BC, Kitimat-Stikine R.D. (55.2564, -127.606) | | |  | KC251539 | KC251265 | KC251037 | Yes |
|  |  | 4x | Coughlan, J.; Zarrei, M.; Shaw, C. | 2010-35 | 06-Nov-2011 | TRT285 | TRT00002609 | BC, Kitimat-Stikine R.D. (55.0989, -128.076) | | |  | KC251534 | KC251260 | KC251032 | Yes |
|  |  | 4x | Coughlan, J.; Zarrei, M.; Shaw, C. | JC281 | 30-May-2011 | ***TRT196*** | ***TRT00020272*** | MT, Sanders Co. (47.3445, -114.719) | | |  | KC251585 | KC251312 | KC251068 | Yes |
|  |  | 4x | Coughlan, J.; Zarrei, M.; Shaw, C. | JC380 | 06-May-2011 | TRT283 | TRT00020204 | BC, Capital R.D. (48.4643, -123.384) | | |  | KC251533 | KC251259 | KC251031 | Yes |
|  |  | 4x | Coughlan, J.; Zarrei, M.; Shaw, C. | JC385 | 06-May-2011 | ***TRT245*** | ***TRT00020211*** | BC, Capital R.D. (48.8103, -123.751) | | |  | KC251535 | KC251261 | KC251033 | Yes |
|  |  | 4x | Coughlan, J.; Zarrei, M.; Shaw, C. | JC387 | 06-Jun-2011 | ***TRT244*** | ***TRT00020213*** | BC, Cowichan Valley R.D. (48.7903, -123.71) | | |  | KC251536 | KC251262 | KC251034 | Yes |
|  |  | 4x | Coughlan, J.; Zarrei, M.; Shaw, C. | JC388 | 06-Jun-2011 | TRT284 | TRT00020216 | BC, Cowichan Valley R.D. (48.7911, -123.709) | | |  | KC251532 | KC251258 | KC251030 | Yes |
|  |  | 4x | Dickinson, T.A.; Coughlan, J.; Zarrei, M. | 2010-28 | 13-Aug-2010 | TRT216 | TRT00002602 | BC, Cariboo R.D. (53.9483, -122.951) | | |  | KC251541 | KC251267 | KC251039 | Yes |
|  |  | 4x | Dickinson, T.A.; Coughlan, J.; Zarrei, M. | 2010-29 | 14-Aug-2010 | TRT217 | TRT00002603 | BC, Kitimat-Stikine R.D. (55.2187, -127.445) | | |  | KC251540 | KC251266 | KC251038 | Yes |
|  |  | 4x | Dickinson, T.A.; Dickinson, A.K. | 2007-22 | 17-Jun-2007 | TRT160 | TRT00001146 | ON, Thunder Bay Dist. (48.451, -89.193) | | |  | KC251548 | KC251274 |  | Yes |
|  |  | 4x | Dickinson, T.A.; Dickson, E.E.; Dickinson, A.K. | 2007-19 | 10-Jun-2007 | TRT159 |  | SK, Cypress Hills (49.6572, -109.504) | | |  | KC251547 | KC251273 |  | Yes |
|  |  | 4x | Dickinson, T.A.; Lee, J., Talent N. | 2008-36 | 30-May-2008 | TRT170 | TRT00002467 | BC, Central Kootenay (49.7522, -117.468) | | |  | KC251549 | KC251275 | KC251044 | Yes |
|  |  | 4x | Dickinson, T.A.; Lee, J.; Talent N. | 2008-46 | 01-Jun-2008 | ***TRT240*** | ***TRT00002484*** | BC, North Okanagan R.D. (50.19, -119.25) | | |  | KC251538 | KC251264 | KC251036 | Yes |
|  |  | 4x | Lo, E.; Dickinson, T.A.; Nguyen, S. | EL-166 | 15-Jun-2004 | TRT175 | TRT00002027 | ID, Adams Co. (44.9889, -116.19) | | |  | KC251550 | KC251276 |  | Yes |
|  |  | 4x | Lo, E.; Dickinson, T.A.; Nguyen, S. | EL-170 | 15-Jun-2004 | TRT177 | TRT00001279 | ID, Adams Co. (44.9878, -116.189) | | |  | KC251551 | KC251277 | KC251045 | Yes |
|  |  | 4x | Lo, E.; Dickinson, T.A.; Nguyen, S. | EL-32 | 07-Jun-2004 | TRT184 | TRT00002023 | MT, Powell Co. (47.0278, -112.937) | | |  | KC251531 | KC251257 |  | Yes |
|  |  | 4x | Lo, E.; Dickinson, T.A.; Nguyen, S. | EL-39 | 07-Jun-2004 | TRT004 | TRT00001302 | MT, Powell Co. (47.0202, -112.946) | | | KC861791 | KC251552 | KC251278 |  | Yes |
|  |  | 4x | Lo, E.; Talent, N. | EL11 | 01-Jun-2004 | TRT001 | TRT00001145 | ON, Grey Co. (44.806, -81) | | |  | KC251553 | KC251279 |  | Yes |
|  |  | NA | Love, R.M. | C-2003-22F | 09-Apr-2003 | TRT041 | TRT00001382 | OR, Grant Co. (44.5877, -119.641) | | |  | KC251544 | KC251270 |  | Yes |
|  |  | 4x | Nguyen, S.; Dickinson, T.A. | NT190 | 27-May-2003 | TRT157 |  | ON, Grey Co. (44.8064, -80.9802) | | |  | KC251546 | KC251272 | KC251043 | Yes |
|  |  | 4x | Talent, N.; Hirst, G. | NT611 |  | TRT243 |  | ON, Manitoulin District (45.8962, -82.5292) | | |  | KC251537 | KC251263 | KC251035 | Yes |
|  |  | 4x | Zika, P.F. | 18453 | 07-Jun-2003 | TRT145 | TRT00003801 | BC, North Okanagan R.D. (50.51, -119.103) | | |  | KC251545 | KC251271 |  | Yes |
|  |  | 5x | Coughlan, J.; Zarrei, M.; Shaw, C. | 2010-31 | 14-Aug-2011 | ***TRT199*** | ***TRT00002605*** | BC, Kitimat-Stikine R.D. (55.2568, -127.607) | | |  |  | KC251269 | KC251042 | No |
|  |  | 5x | Coughlan, J.; Zarrei, M.; Shaw, C. | 2010-38 | 15-Aug-2011 | ***TRT200*** | ***TRT00002612*** | BC, Kitimat-Stikine R.D. (54.4447, -128.762) | | |  | KC251543 | KC251268 | KC251041 | Yes |
|  | *C. enderbyensis* J.B. Phipps & O’Kennon | | |  |  |  |  |  | | |  |  |  |  |  |
|  |  | 4x | Zika, P.F. | 18445 | 07-Jun-2003 | ***TRT152*** | ***TRT00004465*** | BC, North Okanagan R.D. (50.55, -119.13) | | | KC861793 | KC251556 | KC251282 | KC251048 | Yes |
|  |  | 4x | Zika, P.F. | 18446 | 07-Jun-2003 | TRT149 | TRT00004466 | BC, North Okanagan R.D. (50.55, -119.13) | | | KC861792 | KC251557 | KC251283 | KC251049 | Yes |
|  |  | 4x | Zika, P.F. | 18451 | 07-Jun-2003 | TRT147 | TRT00004401 | BC, North Okanagan R.D. (50.55, -119.13) | | | KC861794 | KC251554 | KC251280 | KC251046 | Yes |
|  |  | 4x | Zika, P.F. | 18454 | 07-Jun-2003 | TRT153 | TRT00004399 | BC, North Okanagan R.D. (50.525, -119.137) | | |  | KC251555 | KC251281 | KC251047 | Yes |
|  | *C. gaylussacia* A. Heller | | |  |  |  |  |  | | |  |  |  |  |  |
|  |  | 3x | Coughlan, J.; Zarrei, M.; Shaw, C. | JC005 | 05-Aug-2011 | TRT220 | TRT00020104 | CA, Sonoma Co. (38.4044, -122.849) | | |  | KC251563 | KC251289 | KC251055 | Yes |
|  |  | 3x | Coughlan, J.; Zarrei, M.; Shaw, C. | JC014 | 05-Aug-2011 | TRT221 | TRT00020115 | CA, Marin Co. (38.247, -122.915) | | | KC861799 | KC251564 | KC251290 | KC251056 | Yes |
|  |  | 3x | Coughlan, J.; Zarrei, M.; Shaw, C. | JC015 | 05-Aug-2011 | TRT222 | TRT00020117 | CA, Marin Co. (38.247, -122.914) | | |  | KC251565 | KC251291 | KC251057 | Yes |
|  |  | 3x | Coughlan, J.; Zarrei, M.; Shaw, C. | JC018 | 05-Sep-2011 | TRT223 | TRT00020122 | CA, Sonoma Co. (38.3596, -122.8) | | | KC861798 |  | KC251292 | KC251058 | Yes |
|  |  | 3x | Coughlan, J.; Zarrei, M.; Shaw, C. | JC019 | 05-Sep-2011 | TRT224 | TRT00020124 | CA, Sonoma Co. (38.3596, -122.8) | | |  | KC251566 | KC251293 | KC251059 | Yes |
|  |  | 3x | Shiller, J.; Tusha, J.; Dickinson, T.A.; Heckel, M. | PORE-509-1 | 26-Apr-2010 | ***TRT239*** | ***TRT00002016*** | CA, Marin Co. (38.1156, -122.897) | | | KC861800 | KC251562 | KC251288 | KC251054 | Yes |
|  | *C. okennoni* J.B. Phipps | | |  |  |  |  |  | | |  |  |  |  |  |
|  |  | 4x? | Coughlan, J.; Zarrei, M.; Shaw, C. | JC296 | 31-May-2011 | ***TRT197*** | ***TRT00020275*** | MT, Lake Co. (47.6756, -114.159) | | |  | KC251586 | KC251313 | KC251069 | Yes |
|  |  | 4x | Dickinson, T.A.; Dickinson, A.K.; Lee, J.; Lo, B. | 2007-10 | 06-Jul-2007 | TRT169 | TRT00004195 | BC, Central Kootenay (49.805, -118.159) | | |  | KC251633 | KC251358 |  | Yes |
|  |  | 4x | Lo, E.; Dickinson, T.A.; Nguyen, S. | EL-151 | 14-Jun-2004 | TRT171 | TRT00001551 | WA, Whitman Co. (46.852, -117.34) | | |  | KC251464 | KC251189 |  | Yes |
|  |  | 4x | Lo, E.; Dickinson, T.A.; Nguyen, S. | EL-152 | 14-Jun-2004 | ***TRT172*** | ***TRT00001550*** | WA, Whitman Co. (46.8536, -117.342) | | |  | KC251632 | KC251357 | KC251109 | Yes |
|  |  | 4x | Lo, E.; Dickinson, T.A.; Nguyen, S. | EL-153 | 14-Jun-2004 | TRT173 | TRT00001549 | WA, Whitman Co. (46.8536, -117.342) | | |  | KC251631 | KC251356 |  | Yes |
|  | *C. shuswapensis* J.B. Phipps & O’Kennon | | |  |  |  |  |  | | |  |  |  |  |  |
|  |  | 4x | Dickinson, T.A.; Dickinson, A.K.; Lee, J.; Lo, B. | 2007-11 | 06-Jul-2007 | ***TRT167*** | ***TRT00004219*** | BC, Central Kootenay (49.805, -118.159) | | | KC861878 | KC251672 | KC251399 |  | Yes |
|  |  | 5x | Dickinson, T.A.; Lee, J., Talent N. | 2008-38 | 30-May-2008 | TRT166 | TRT00002470 | BC, Central Kootenay (49.752, -117.468) | | |  | KC251463 | KC251188 |  | Yes |
|  | *C. suksdorfii* (Sarg.) Kruschke | | |  |  |  |  |  | | |  |  |  |  |  |
|  |  | 2x | Coughlan, J.; Zarrei, M.; Shaw, C. | JC039 | 11-May-2011 | TRT191 | TRT00020137 | OR, Jackson Co. (42.4307, -123.094) | | |  | KC251694 |  | KC251146 | No |
|  |  | 2x | Coughlan, J.; Zarrei, M.; Shaw, C. | JC060 | 13-May-2011 | TRT192 | TRT00020146 | OR, Linn Co. (44.5626, -123.152) | | |  | KC251695 |  | KC251147 | No |
|  |  | 2x | Coughlan, J.; Zarrei, M.; Shaw, C. | JC092 | 17-May-2011 | TRT193 | TRT00020153 | OR, Skamania Co. (45.6719, -121.887) | | | KC861897 | KC251696 | KC251421 | KC251148 | Yes |
|  |  | 2x | Coughlan, J.; Zarrei, M.; Shaw, C. | JC094 | 17-May-2011 | TRT225 | TRT00020157 | OR, Skamania Co. (45.6723, -121.886) | | | KC861891 | KC251700 | KC251425 | KC251152 | Yes |
|  |  | 2x | Coughlan, J.; Zarrei, M.; Shaw, C. | JC098 | 17-May-2011 | TRT194 | TRT00020159 | OR, Multnomah Co. (45.5557, -122.363) | | | KC861898 | KC251697 | KC251422 | KC251149 | Yes |
|  |  | 2x | Coughlan, J.; Zarrei, M.; Shaw, C. | JC102 | 17-May-2011 | TRT226 | TRT00020163 | OR, Multnomah Co. (45.5515, -122.374) | | | KC861896 | KC251701 | KC251426 | KC251153 | Yes |
|  |  | 2x | Coughlan, J.; Zarrei, M.; Shaw, C. | JC103 | 17-May-2011 | TRT227 | TRT00020165 | OR, Multnomah Co. (45.5509, -122.377) | | | KC861892 | KC251702 | KC251427 | KC251154 | Yes |
|  |  | 2x | Coughlan, J.; Zarrei, M.; Shaw, C. | JC110 | 18-May-2011 | TRT228 | TRT00020169 | OR, Columbia Co. (45.6949, -122.798) | | | KC861893 | KC251703 | KC251428 | KC251155 | Yes |
|  |  | 2x | Coughlan, J.; Zarrei, M.; Shaw, C. | JC114 | 18-May-2011 | TRT230 | TRT00020171 | OR, Columbia Co. (45.7292, -122.767) | | |  | KC251705 | KC251430 | KC251157 | Yes |
|  |  | 2x | Coughlan, J.; Zarrei, M.; Shaw, C. | JC117 | 18-May-2011 | TRT231 | TRT00020172 | OR, Columbia Co. (45.7326, -122.767) | | | KC861890 | KC251706 | KC251431 | KC251158 | Yes |
|  |  | 2x | Coughlan, J.; Zarrei, M.; Shaw, C. | JC118 | 18-May-2011 | TRT232 | TRT00020232 | OR, Columbia Co. (45.7338, -122.767) | | | KC861884 | KC251707 | KC251432 | KC251159 | Yes |
|  |  | 2x | Coughlan, J.; Zarrei, M.; Shaw, C. | JC119 | 18-May-2011 | TRT233 | TRT00020234 | OR, Columbia Co. (45.7326, -122.767) | | | KC861883 | KC251708 | KC251433 | KC251160 | Yes |
|  |  | 2x | Coughlan, J.; Zarrei, M.; Shaw, C. | JC136 | 20-May-2011 | TRT195 | TRT00020242 | OR, Columbia Co. (46.1106, -122.984) | | | KC861899 | KC251698 | KC251423 | KC251150 | Yes |
|  |  | 2x | Dickinson, T. A.; Lo, E. | 2006-16 | 28-Jul-2006 | TRT140 | TRT00001567 | CA, Siskiyou Co. (41.3973, -122.838) | | | KC861886 | KC251690 | KC251417 |  | Yes |
|  |  | 2x | Dickinson, T. A.; Lo, E. | 2006-18 | 28-Jul-2006 | TRT141 | TRT00001568 | CA, Siskiyou Co. (41.3973, -122.838) | | | KC861887 | KC251689 | KC251416 |  | Yes |
|  |  | 2x | Dickinson, T. A.; Lo, E. | 2006-19 | 28-Jul-2006 | TRT129 | TRT00001569 | CA, Siskiyou Co. (41.3973, -122.838) | | | KC861885 | KC251692 | KC251419 |  | Yes |
|  |  | 2x | Lo, E.; Dickinson, T. A. | 2006-22 | 28-Jul-2006 | TRT133 | TRT00001563 | CA, Siskiyou Co. (41.3973, -122.838) | | |  | KC251691 | KC251418 |  | Yes |
|  |  | 2x | Lo, E.; Dickinson, T.A.; Nguyen, S. | EL-68 | 10-Jun-2004 | TRT205 | TRT00001724 | OR, Linn Co. (44.333, -123.122) | | |  | KC251699 | KC251424 | KC251151 | Yes |
|  |  | 2x | Zika, P.F. | 18485 | 15-Jun-2003 | ***TRT146*** | ***TRT00003669*** | WA, Clark Co. (45.8313, -122.755) | | |  | KC251688 | KC251415 |  | Yes |
|  |  | 3x | Coughlan, J.; Zarrei, M.; Shaw, C. | JC375 | 06-May-2011 | TRT281 | TRT00020194 | BC, Capital R.D. (48.436, -123.458) | | |  | KC251693 | KC251420 | KC251145 | Yes |
|  |  | 3x | Coughlan, J.; Zarrei, M.; Shaw, C. | JC383 | 06-May-2011 | TRT282 | TRT00020209 | BC, Capital R.D. (48.4654, -123.385) | | |  | KC251709 | KC251434 | KC251161 | Yes |
|  |  | 3x | Lo, E.; Dickinson, T.A.; Nguyen, S.; Love, R.M. | EL-57 | 09-Jun-2004 | TRT008 | TRT00001740 | OR, Lane Co. (43.7675, -122.617) | | | KC861889 | KC251685 | KC251412 |  | Yes |
|  |  | 3x | Lo, E.; Dickinson, T.A.; Nguyen, S.; Love, R.M. | EL-62 | 09-Jun-2004 | TRT009 | TRT00001763 | OR, Lane Co. (43.7675, -122.617) | | |  | KC251686 | KC251413 |  | Yes |
|  |  | 3x | Lo, E.; Dickinson, T.A.; Nguyen, S.; Love, R.M. | EL-65 | 09-Jun-2004 | TRT010 | TRT00001760 | OR, Lane Co. (43.7675, -122.617) | | | KC861888 | KC251687 | KC251414 |  | Yes |
|  |  | 3x | Lo, E.; Dickinson, T.A.; Nguyen, S. | EL-165 | 15-Jun-2004 | TRT174 | TRT00001605 | ID, Adams Co. (44.9889, -116.19) | | |  | KC251678 | KC251405 |  | Yes |
|  |  | 3x | Lo, E.; Dickinson, T.A.; Nguyen, S. | EL-172 | 15-Jun-2004 | ***TRT178*** | ***TRT00001606*** | ID, Adams Co. (44.9878, -116.189) | | |  | KC251683 | KC251410 |  | Yes |
|  |  | 3x | Lo, E.; Dickinson, T.A.; Nguyen, S. | EL-173 | 15-Jun-2004 | TRT176 | TRT00001602 | ID, Adams Co. (44.9878, -116.189) | | | KC861895 | KC251684 | KC251411 |  | Yes |
|  |  | 3x | Lo, E.; Dickinson, T.A.; Nguyen, S. | EL-188 | 15-Jun-2004 | TRT179 | TRT00001640 | ID, Valley Co. (44.996, -116.063) | | |  | KC251682 | KC251409 |  | Yes |
|  |  | 4x | Lo, E.; Dickinson, T.A.; Nguyen, S. | EL-30 | 07-Jun-2004 | ***TRT002*** | ***TRT00001581*** | MT, Powell Co. (47.038, -112.922) | | |  | KC251680 | KC251407 | KC251143 | Yes |
|  |  | 4x | Lo, E.; Dickinson, T.A.; Nguyen, S. | EL-36 | 07-Jun-2004 | TRT185 | TRT00001583 | MT, Powell Co. (47.0202, -112.946) | | |  | KC251679 | KC251406 |  | Yes |
|  |  | 4x | Lo, E.; Dickinson, T.A.; Nguyen, S. | EL-45 | 07-Jun-2004 | TRT003 | TRT00001577 | MT, Powell Co. (47.0202, -112.946) | | |  | KC251681 | KC251408 | KC251144 | Yes |
|  |  | NA | Coughlan, J.; Zarrei, M.; Shaw, C. | JC111 | 18-May-2011 | TRT229 | TRT00020170 | OR, Columbia Co. (45.6948, -122.798) | | | KC861894 | KC251704 | KC251429 | KC251156 | Yes |
|  | ***Purpureofructus*** J.B. Phipps & O’Kennon | | |  |  |  |  |  | | |  |  |  |  |  |
|  | *C. aquacervensis* J.B. Phipps & O’Kennon | | |  |  |  |  |  | | |  |  |  |  |  |
|  |  | 4x | Dickinson, T.A.; Dickson, E.E.; Dickinson, A.K. | 2007-12 | 06-Sep-2007 | ***TRT162*** | ***TRT00004665*** | AB, Cypress Co. (49.676, -110.318) | | | KC861769 |  | KC251213 | KC250995 | Yes |
|  |  | 4x | Dickinson, T.A.; Dickson, E.E.; Dickinson, A.K. | 2007-14 | 06-Oct-2007 | TRT161 | TRT00001933 | AB, Cypress Co. (49.6456, -110.387) | | | KC861768 | KC251487 | KC251212 | KC250994 | Yes |
|  | *C. atrovirens* J.B. Phipps & O’Kennon | | |  |  |  |  |  | | |  |  |  |  |  |
|  |  | NA | Jackson, R. (for Phipps, J.B.) | 8372 | 20-May-2002 | MKTRT593 | TRT00004311 | BC, North Okanagan R.D. (50.3667, -119.217) | | |  |  | KP050247 |  | Yes |
|  |  | NA | Phipps, J.B.; O’Kennon, R.J. | 6996 | 19-Aug-1994 | MKTRT592 | TRT00004298 | BC, North Okanagan R.D. (50.305, -119.247) | | |  |  | KP050234 |  | Yes |
|  |  | NA | Phipps, J.B. .; O’Kennon, R.J. | 8318 | 12-May-2002 | MKTRT594 | TRT00004307 | BC, North Okanagan R.D. (50.35, -119.242) | | |  |  | KP050292 | KP050192 | Yes |
|  | *C. okanaganensis* J.B. Phipps & O’Kennon | | |  |  |  |  |  | | |  |  |  |  |  |
|  |  | NA | Phipps, J.B. | 7017 | 20-Aug-1994 | MKTRT590 | TRT00004356 | BC, North Okanagan R.D. (50.55, -119.117) | | |  |  | KP050229 |  | Yes |
|  |  | NA | Phipps, W.T.J. | 6.7 | 28-Sep-1999 | MKTRT589 | TRT00004347 | BC, North Okanagan R.D. (50.517, -119.103) | | |  |  | KP050273 |  | Yes |
|  |  | NA | Phipps, W.T.J. | 2.5 | 28-Sep-1999 | MKTRT591 | TRT00004360 | BC, North Okanagan R.D. (50.41, -119.26) | | |  | KP050207 | KP050248 | KP050166 | Yes |
|  | *C. orbicularis* J.B. Phipps & O’Kennon | | |  |  |  |  |  | | |  |  |  |  |  |
|  |  | NA | Jackson, R. (for Phipps, J.B.) | 8304 | 23-May-2002 | MKTRT588 | TRT00004482 | BC, North Okanagan R.D. (50.5667, -119.1) | | |  | KP050213 | KP050266 | KP050177 | Yes |
|  |  | NA | Jackson, R. (for Phipps, J.B.) | 8403 | 23-May-2002 | MKTRT586 | TRT00004485 | BC, North Okanagan R.D. (50.5167, -119.117) | | |  | KP050219 | KP050282 | KP050187 | Yes |
|  |  | NA | Phipps, J.B. | 8371 | 18-May-2002 | MKTRT587 | TRT00004476 | BC, North Okanagan R.D. (50.5133, -119.102) | | |  | KP050212 | KP050258 | KP050173 | Yes |
|  | *C. phippsii* O’Kennon | | |  |  |  |  |  | | |  |  |  |  |  |
|  |  | NA | Coughlan, J.; Zarrei, M.; Shaw, C. | JC178 | 23-May-2011 | ***TRT246*** | ***TRT00020265*** | WA, Okanogan Co. (48.9158, -119.632) | | | KC861857 | KC251642 | KC251367 | KC251116 | Yes |
|  |  | NA | Phipps, J.B.; O’Kennon, R.J. | 8323 | 12-May-2002 | MKTRT613 | TRT00004504 | BC, North Okanagan R.D. (50.349, -119.244) | | |  | KP050208 | KP050251 | KP050168 | Yes |
|  |  | NA | Phipps, J.B. | 8175 | 24-Aug-2000 | MKTRT615 | TRT00004515 | BC, North Okanagan R.D. (50.474, -119.116) | | |  |  | KP050265 |  | Yes |
|  |  | NA | Phipps, J.B. | 6880 | 02-May-1994 | MKTRT614 | TRT00004516 | BC, Okanagan Valley (50.329, -119.24) | | |  |  | KP050275 | KP050183 | Yes |
|  | *C. williamsii* Eggleston | | |  |  |  |  |  | | |  |  |  |  |  |
|  |  | NA | Dickinson, T.A.; Gervais, G.Y.F.; Dickinson, J.S. | 2001-20 | 31-May-2001 | TRT034 | TRT00001952 | MT, Lake Co. (47.718, -114.202) | | | KC861912 | KC251475 | KC251200 | KC250983 | Yes |
|  | *C. cupressocollina* J.B. Phipps & O’Kennon | | |  |  |  |  |  | | |  |  |  |  |  |
|  |  | 4x | Dickinson, T.A.; Dickson, E.E.; Dickinson, A.K. | 2007-16 | 10-Jun-2007 | TRT155 | TRT00004548 | SK, Cypress Hills (49.624, -109.631) | | |  | KC251525 | KC251251 | KC251025 | Yes |
|  |  | 4x | Dickinson, T.A.; Dickson, E.E.; Dickinson, A.K. | 2007-20 | 10-Jun-2007 | ***TRT163*** | ***TRT00004547*** | SK, Cypress Hills (49.666, -109.463) | | |  | KC251526 | KC251252 | KC251026 | Yes |
|  | ***Montaninsulae*** J.B. Phipps & O’Kennon | | |  |  |  |  |  | | |  |  |  |  |  |
|  | *C. purpurella* J.B. Phipps & O’Kennon | | |  |  |  |  |  | | |  |  |  |  |  |
|  |  | NA | Nagel-Hisey, M.; Goett, R. | 3 | 09-Jun-2004 | MKTRT595 | TRT00003630 | SK, Cypress Hills Provincial Park (49.68, -109.5) | | |  |  | KP050228 | KP050152 | Yes |
|  |  | NA | Nagel-Hisey, M. | 2 | 31-May-2003 | MKTRT596 | TRT00003634 | SK, Cypress Hills Provincial Park (49.653, -109.495) | | |  |  | KP050268 | KP050179 | Yes |
|  |  | NA | Phipps, J.B. ; O’Kennon, R.J. | 8690 | 27-May-2004 | MKTRT597 | TRT00003639 | SK, Maple Creek R.M. (49.7178, -109.453) | | |  |  | KP050290 | KP050191 | Yes |
|  | *C. rivuloadamensis* J.B. Phipps & O’Kennon | | |  |  |  |  |  | | |  |  |  |  |  |
|  |  | 4x | Dickinson, T.A.; Dickson, E.E.; Dickinson, A.K. | 2007-13 | 06-Sep-2007 | ***TRT168*** | ***TRT00003557*** | AB, Cypress Co. (49.655, -110.348) | | | KC861876 | KC251663 | KC251390 |  | Yes |
|  | *C. rivulopugnensis* J.B. Phipps & O’Kennon | | |  |  |  |  |  | | |  |  |  |  |  |
|  |  | 4x | Dickinson, T.A.; Dickson, E.E.; Dickinson, A.K. | 2007-21 | 10-Jun-2007 | ***TRT154*** | ***TRT00003524*** | SK, Cypress Hills (49.626, -109.971) | | |  | KC251664 | KC251391 | KC251132 | Yes |
| E. ***Sanguineae*** Zabel ex C.K. Schneid. | | | |  |  |  |  |  | | |  |  |  |  |  |
|  | ***Nigrae*** (Loudon) Russanov | | |  |  |  |  |  | | |  |  |  |  |  |
|  | *C. chlorosarca* Maxim. | | |  |  |  |  |  | | |  |  |  |  |  |
|  |  | 2x | Dickinson, T.A. | 2000-63 | 20-May-2003 | TRT046 | TRT00001996 | MA, Suffolk Co. Arnold Arboretum (42.3031, -71.1242) AA281-71A | | |  | KC251512 | KC251239 | KC251012 | Yes |
|  | *C. kansuensis* Wilson | | |  |  |  |  |  | | |  |  |  |  |  |
|  |  | NA | Dickinson, T.A. | 2002-02A | 20-Jun-2002 | TRT045 | TRT00001997 | MA, Suffolk Co. (42.3031, -71.1242) | | | KC861820 | KC251589 | KC251316 | KC251072 | Yes |
|  | *C. maximowiczii* C.K. Schneid. | | |  |  |  |  |  | | |  |  |  |  |  |
|  |  | NA | Dickinson, T.A. | 2002-04A | 20-Jun-2002 | TRT044 | TRT00001991 | MA, Suffolk Co. (42.3031, -71.1242) | | | KC861834 | KC251605 | KC251331 |  | Yes |
|  |  | NA | Romankova, T. | 1 | 15-Jun-2005 | TRT095 | TRT00002372 | Russia Primorsky Krai, Vladivostok, Botanical Garden of the Academy of Science. (43.128, 131.891) | | |  | KC251604 | KC251330 |  | Yes |
|  |  | NA | Romankova, T. | 4 | 23-Jun-2005 | ***TRT094*** | ***TRT00002370*** | Russia Primorsky Krai, Lazovskiy Zapovednik (reserve), Lazo, Lazovka River Valley (43.4247, 134.028) | | |  | KC251603 | KC251329 |  | Yes |
|  |  | NA | Romankova, T. | 5 | 24-Jun-2005 | TRT093 | TRT00002371 | Russia Primorsky Krai, Lazovskiy Zapovednik (reserve), Lazo, Lazovka River Valley (43.4247, 134.028) | | |  | KC251606 | KC251332 |  | Yes |
|  |  | 4x | Christensen, K.I. | KIC 287 | 01-Jan-2004 | TRT053 |  | Denmark , Taastrup (55.65, 12.3) | | |  | KC251665 | KC251392 | KC251133 | Yes |
|  | *C. nigra* Waldst. & Kit. | | |  |  |  |  |  | | |  |  |  |  |  |
|  |  | NA | Christensen, K.I. | Arboretum No 1248-1915 1041-3336 on the silica bag | 01-Jan-2004 | TRT054 |  | Denmark , Material grown in the Arboretum, Hørsholm, Royal Agricultural and Veterinary University, from seed from Hesse Nursery, Germany (1941). (55.8699, 12.5029) | | |  | KC251628 | KC251353 | KC251107 | Yes |
|  |  | 2x | Christensen, K.I. | KIC 294 | 21-Mar-2005 | ***TRT036*** | ***TRT00002052*** | Bulgaria , Sofia, Hortus Botanicus of the Institute Silvotechnici Superioris (42.7, 23.325) | | | KC861849 | KC251630 | KC251355 |  | Yes |
|  |  | 2x | Dickinson, T.A.; Evans, R.C. | s.n. | 31-May-1999 | TRT025 | TRT00002000 | QC, Montréal, Jardin Botanique de Montréal, Arboretum. (45.5599, -73.5662) 2318-50 | | | KC861848 | KC251629 | KC251354 | KC251108 | Yes |
|  | ***Sanguineae*** (Zabel ex C.K. Schneid.) Rehder | | |  |  |  |  |  | | |  |  |  |  |  |
|  | *C. dahurica* Koehne ex C.K. Schneid. | | |  |  |  |  |  | | |  |  |  |  |  |
|  |  | NA | Dickinson, T.A. | s.n. | 20-Jun-2002 | TRT055 |  | MA, Suffolk Co. Arnold Arboretum (42.303, -71.124) AA-EN 250-2000 | | |  | KC251527 | KC251253 | KC251027 | Yes |
|  |  | 2x | Dickinson, T.A. = *maximowiczii* per KIC? | s.n. | 20-May-2000 | TRT029 | TRT00002060 | MA, Suffolk Co. Arnold Arboretum (42.3031, -71.1242) AA71-73A | | |  | KC251528 | KC251254 |  | Yes |
|  | *C. wilsoni* Sarg. | | |  |  |  |  |  | | |  |  |  |  |  |
|  |  | 2x | Dickinson, T.A. | s.n. | 9-Oct-1999 | ***TRT114*** | ***TRT00002055*** | MA, Suffolk Co. (Arnold Arboretum 42.2939, -71.1265) AA749-74A | | | KC861913 | KC251726 | KC251451 | KC251177 | Yes |
|  |  | NA | Dickinson, T.A. | s.n. | 21-May-2000 | TRT122 | TRT00000165 | MA, Suffolk Co. Arnold Arboretum (42.2939, -71.1265) | | | KC861914 | KC251725 | KC251450 | KC251176 | Yes |
|  |  |  |  |  |  |  |  |  | | |  |  |  |  |  |
| **Outgroups** | | | |  |  |  |  |  | | |  |  |  |  |  |
| *Amelanchier* | | | |  |  |  |  |  | | |  |  |  |  |  |
|  | *A. alnifolia* Nutt. | | | JC431 |  |  | TRT00021066 | (Zarrei *et al.* 2014) | KC174283, KC174284, KC174285, KC174286, | | | | | |  |
|  | *A. arborea* (F. Michx.) Fernald | | | 2003-1 |  |  | TRT00034837 | (Lo et al., 2007) | | EF127041 | |  |  |  |  |
|  | *A. bartramiana* (Tausch) M. Roem. | | | B5 |  |  | voucher in MAINE | (Lo *et al.* 2009) | | EU500453 | |  |  |  |  |
| *Malus* | | | |  |  |  |  |  | |  | |  |  |  |  |
|  | *M. prunifolia* | | |  |  |  |  | Robinson JP, Harris SA, Juniper BE. 2001. Taxonomy of the genus *Malus* Mill. (Rosaceae) with emphasis on the cultivated apple, *Malus domestica* Borkh. Plant Systematics and Evolution, 226: 35-58. | | AF186500 | |  |  |  |  |
|  | *M. toringoides* Hughes | | |  |  |  |  | (Feng et al. unpubl.) | | EF113186 | |  |  |  |  |
| *Pyrus ussuriensis* Maxim. | | | |  |  |  |  | Zheng X, Cai D, Yao L, Teng Y. 2008. Non-concerted ITS evolution, early origin and phylogenetic utility of ITS pseudogenes in *Pyrus*. Molecular Phylogenetics & Evolution, 48: 892-903. | | EU149965 | |  |  |  |  |
| *Sorbus hupehensis* C.K. Schneid. | | | |  |  |  |  | (Li et al., 2012) | | EF113186 | |  |  |  |  |
|  |  |  |  |  |  |  |  |  | |  | |  |  |  |  |
