# Supplementary material for: DNA barcodes from four loci provide poor resolution of taxonomic groups in the genus Crataegus
Source: AoB Plants. 2015 Apr 29;7:plv045. doi: 10.1093/aobpla/plv045 (PMC4480070; doi:10.1093/aobpla/plv045)
Supplement: Additional Information [file supp_plv045_plv045supp_table2.doc]

Table S2. Phylogenetic analysis of 14 *Crataegus* plastid DNA loci (Fig. 1). Voucher information and GenBank accssion numbers for 11 supplementary plastid DNA loci and the three plastid DNA barcode loci (Table S1; further details of the barcode sequences are available at dx.doi.org/10.5883/DS-NAMCRAT). Additional sources for outgroup sequences from GenBank: 1. Lo (2008); 2. Verbylaitė et al. (2006); 3. Terakami,S., Matsumura,Y., Kurita,K., Kanamori,H., Katayose,Y., Yamamoto,T. and Katayama,H. (unpublished). Complete sequences of the chloroplast genome from pear (*Pyrus pyrifolia*): genome structure and comparative analysis; 4. Zheng,X., Cai,D., Potter,D., Postman,J., Liu,J. and Teng,Y. (unpublished). Phylogeny and evolutionary histories of *Pyrus* species revealed by phylogenetic trees and networks based on multiple DNA sequence data; 5. Chen,S.-L. and Pang,X.-H. (unpublished). Plant DNA barcodes and species resolution in Rosaceae. Data for accessions with TRT and BOLD numbers in bolded italics are found in Table S1.

| **Section** | | | |  | |  |  | **GenBank** | |  |  |  |  |  |  |  |  |  |  |  |  |  | |
| --- | --- | --- | --- | --- | --- | --- | --- | --- | --- | --- | --- | --- | --- | --- | --- | --- | --- | --- | --- | --- | --- | --- | --- |
|  | **Series** | | **Collector and number** | | | **BOLD** | **TRT** | ***trn*G*-trn*S** | ***rpl*2*-trn*H** | ***rpl*20*-rps*12** | ***trn*L*-trn*F** | ***atp*B*-rbc*L** | ***rps*16** | ***rpl*16** | ***trn*C*-yfc*6** | ***acc*D** | ***rpo*C1** | ***atp*F*-atp*H** | ***mat*K** | ***rbcLa*** | ***psb*A*-trn*H** |  | |
| A. ***Mespilus*** T.A. Dickinson & E.Y.Y. Lo | | | |  | |  |  |  |  |  |  |  |  |  |  |  |  |  |  |  |  |  | |
|  | *C. germanica* (L.) K. Koch | | |  | |  |  |  |  |  |  |  |  |  |  |  |  |  |  |  |  |  | |
|  |  | NA | Hess, W.; Linden, M. | 6216V93 | | ***TADCR075*** | ***TRT00020706*** | - | KC206840 | KC206929 | KC206772 | KC206626 | KC207003 | KC206916 | KC207044 | KC206591 | - | KC206554 | KC173390 | Table S1 | Table S1 |  | |
| A. ***Brevispinae*** Beadle ex Schneider | | | |  | |  |  |  |  |  |  |  |  |  |  |  |  |  |  |  |  |  | |
|  | ***Brevispinae*** (Beadle ex Schneider) Rehder | | | | | |  |  |  |  |  |  |  |  |  |  |  |  |  |  |  |  | |
|  | *C. brachyacantha* Sarg. & Engelm. | | | | |  |  |  |  |  |  |  |  |  |  |  |  |  |  |  |  |  | |
|  |  | *2x +* | Dickinson, T.A.; Talent, N.; Nguyen, S. | 2003-32 | | ***TADCR128*** | ***TRT00000023*** | KC206671 | KC206837 | KC206926 | KC206775 | KC206623 | KC207000 | KC206913 | KC207041 | KC206588 | KC174287 | KC206551 | Table S1 | Table S1 | Table S1 |  | |
| B. ***Coccitaegus*** K.I. Chr. & T.A.Dickinson (A) | | | | | | |  |  |  |  |  |  |  |  |  |  |  |  |  |  |  |  | |
|  | ***Punctaegus*** K.I. Christ. & T.A.Dickinson | | | | | |  |  |  |  |  |  |  |  |  |  |  |  |  |  |  |  | |
|  | *C.* × *ninae*-*celottiae* K.I. Christ. & T.A.Dickinson | | | | | | *C.* × *ninae-celottiae* = ♀ *C. monogyna* Jacq. × ♂ *C. punctata* Jacq. (Christensen et al. 2014) | | | | | | | |  |  |  |  |  |  |  |  | |
|  |  | *(2x)* | Purich, M.; Talent, N. | MP84 | | ***TADCR204*** | ***TRT00002249*** | KC206728 | KC206859 | KC206947 | KC206802 | KC206670 | KC206980 | KC206893 | KC207053 | - | - | - | Table S1 | Table S1 | Table S1 |  | |
|  |  | *(2x)* | Purich, M.; Talent, N. | MP85 | | ***TADCR201*** | ***TRT00002250*** | KC206761 | KC206857 | KC206945 | KC206822 | KC206647 | KC206978 | KC206891 | KC207022 | - | KC174307 | KC206562 | Table S1 | Table S1 | Table S1 |  | |
|  |  | *(2x)* | Purich, M.; Talent, N. | MP86 | | ***TADCR202*** | ***TRT00002251*** | KC206727 | KC206858 | KC206946 | KC206801 | KC206648 | KC206979 | KC206892 | KC207023 | KC206607 | KC174308 | KC206563 | Table S1 | Table S1 | Table S1 |  | |
| B. ***Crataegus*** | | | | | |  |  |  |  |  |  |  |  |  |  |  |  |  |  |  |  |  | |
|  | ***Pentagynae*** (C.K. Schneid.) Russanov (A) | | | | | |  |  |  |  |  |  |  |  |  |  |  |  |  |  |  |  | |
|  | *C. pentagyna* Waldst. & Kit. | | | | | |  |  |  |  |  |  |  |  |  |  |  |  |  |  |  |  | |
|  |  | - | Christensen, K.I. | s.n. | | ***TADCR043*** | ***TRT00001887*** | KC206759 | KC206878 | KC206965 | KC206827 | KC206668 | KC206999 | KC206912 | KC207040 | KC206621 | KC174329 | KC206576 | KC173405 | Table S1 | KC207067 |  | |
|  |  | *-* | Christensen, K.I. | s.n. | | ***TADCR098*** | ***TRT00001888*** | KC206762 | - | - | KC206823 | KC206641 | KC206973 | KC206886 | KC207018 | KC206603 | KC174302 | KC206578 | KC173396 | Table S1 | KC207058 |  | |
|  | ***Pinnatifidae*** (Zabel ex C.K. Schneid.) Rehder (A) | | | | | |  |  |  |  |  |  |  |  |  |  |  |  |  |  |  |  | |
|  | *C. pinnatifida* Bunge | | | |  |  |  |  |  |  |  |  |  |  |  |  |  |  |  |  |  |  | |
|  |  | NA | Romankova, T. | | 2 | ***TADCR099*** | ***TRT00002365*** | KC206724 | KC206844 | KC206933 | KC206799 | KC206631 | KC207007 | KC206920 | KC207048 | - | KC174292 | KC206557 | Table S1 | Table S1 | Table S1 |  | |
| D. ***Coccineae*** Loudon | | | | |  |  |  |  |  |  |  |  |  |  |  |  |  |  |  |  |  |  | |
|  | ***Crus-galli*** (Loudon) Rehder (E) | | | |  |  |  |  |  |  |  |  |  |  |  |  |  |  |  |  |  |  | |
|  | *C. crus-galli* L. | | | |  |  |  |  |  |  |  |  |  |  |  |  |  |  |  |  |  |  | |
|  |  | *2x* | Talent, N. | | NT213a | ***TADCR198*** | ***TRT00019161*** | KC206754 | KC206839 | KC206928 | KC206763 | KC206625 | KC207002 | KC206915 | KC207043 | KC206590 | KC174289 | KC206553 | Table S1 | Table S1 | Table S1 |  | |
|  | ***Punctatae***(Loudon) Rehder (E) | | | |  |  |  |  |  |  |  |  |  |  |  |  |  |  |  |  |  |  | |
|  | *C. punctata* Jacq. | | | |  |  |  |  |  |  |  |  |  |  |  |  |  |  |  |  |  |  | |
|  |  | *2x* | Dickinson, T.A.; Nguyen, S. | | BB4 |  | *Unvouchered; sight identification* | KC206747 | KC206877 | - | KC206833 | KC206666 | KC206998 | KC206911 | KC207039 | - | KC174327 | KC206575 | Table S1 | Table S1 | KC207065 |  | |
|  |  |  | ON, Bruce Co. (44.95040, -81.21909) | | | | |  |  |  |  |  |  |  |  |  |  |  |  |  |  |  | |
|  |  | | | | | |  |  |  |  |  |  |  |  |  |  |  |  |  |  |  |  | |
|  | ***Rotundifolieae*** (Eggleston) Rehder (E) | | | | | |  |  |  |  |  |  |  |  |  |  |  |  |  |  |  |  | |
|  | *C. chrysocarpa* var. *piperi* (Britton) Kruschke | | | | | |  |  |  |  |  |  |  |  |  |  |  |  |  |  |  |  | |
|  |  | *4x* | Love, R.M. | s.n. sample 2 | | ***TADCR047*** | ***TRT00018678*** | KC206720 | KC206852 | KC206941 | KC206790 | KC206639 | KC206971 | KC206884 | KC207016 | KC206601 | KC174300 | KC206561 | Table S1 | Table S1 | KC207057 |  | |
|  | ***Triflorae*** (Beadle) Rehder (E) | | |  | |  |  |  |  |  |  |  |  |  |  |  |  |  |  |  |  |  | |
|  | *C. triflora* Chapman | | |  | |  |  |  |  |  |  |  |  |  |  |  |  |  |  |  |  |  | |
|  |  | *2x midpoint -10.08%* | Talent, N.; Lance, R.W. | NT290 | |  | TRT00000351 | KC206753 | KC206879 | KC206966 | KC206835 | KC206642 | KC206974 | KC206887 |  | - | - | - | Table S1 | Table S1 | Table S1 |  | |
|  |  |  | 21-Apr-2004, GA, Floyd Co. (34.20111, -85.38916) | | | | |  |  |  |  |  |  |  |  |  |  |  |  |  |  |  | |
| D. ***Macracanthae*** Loudon | | | |  | |  |  |  |  |  |  |  |  |  |  |  |  |  |  |  |  |  | |
|  | ***Macracanthae*** (Loudon) Rehder (E) | | | | | |  |  |  |  |  |  |  |  |  |  |  |  |  |  |  |  | |
|  | *C. macracantha* Lodd. ex Loudon | | | | | |  |  |  |  |  |  |  |  |  |  |  |  |  |  |  |  | |
|  |  | *4x* | Talent, N. | NT224 | | ***TADCR127*** | ***TRT00018679*** | KC206721 | KC206853 | KC206942 | KC206788 | KC206640 | KC206972 | KC206885 | KC207017 | KC206602 | KC174301 | KC206577 | Table S1 | Table S1 | Table S1 |  | |
| E. ***Crataeglasia*** K.I. Chr. & T.A. Dickinson | | | |  | |  |  |  |  |  |  |  |  |  |  |  |  |  |  |  |  |  | |
|  | ***Crataeglasianae*** K.I. Chr. & T.A. Dickinson (I3) | | | | | |  |  |  |  |  |  |  |  |  |  |  |  |  |  |  |  | |
|  | *Crataegus* × *cogswellii* K.I. Chr. & T.A. Dickinson | | | | | |  |  |  |  |  |  |  |  |  |  |  |  |  |  |  |  | |
|  |  | *2x* | Lo, E.Y.Y.; Dickinson, T.A.; Nguyen, S. | EL-79 | | ***TADCR208*** | ***TRT00002657*** | KC206751 | KC206861 | KC206949 | KC206836 | KC206650 | KC206982 | KC206895 | - | KC206608 | KC174310 | KC206565 | KC173398 | Table S1 | KC207060 |  | |
|  |  | *2x* | Lo, E.Y.Y.; Dickinson, T.A.; Nguyen, S. | EL-85 | | ***TADCR207*** | ***TRT00002654*** | KC206750 | KC206860 | KC206948 | KC206805 | KC206649 | KC206981 | KC206894 | KC207054 | - | KC174309 | KC206564 | Table S1 | Table S1 | Table S1 |  | |
|  | | | |  | |  |  |  |  |  |  |  |  |  |  |  |  |  |  |  |  |  | |
| E. ***Douglasia*** Loudon | | | |  | |  |  |  |  |  |  |  |  |  |  |  |  |  |  |  |  |  | |
|  | ***Cerrones*** J.B. Phipps (F) | | | | | |  |  |  |  |  |  |  |  |  |  |  |  |  |  |  |  |  |
|  | *C. erythropoda* Ashe | | | | | |  |  |  |  |  |  |  |  |  |  |  |  |  |  |  |  |  |
|  |  | *4x* | Talent, N. | NT349 | | ***TADCR241*** | ***TRT00002377*** | KC206736 | KC206866 | KC206954 | KC206817 | KC206655 | KC206987 | KC206900 | KC207028 | KC206614 | KC174316 | KC206585 | Table S1 | Table S1 | Table S1 |  | |
|  | *C. rivularis* Nutt. | | |  | |  |  |  |  |  |  |  |  |  |  |  |  |  |  |  |  |  | |
|  |  | *4x* | Dickinson, T.A.; Gervais, F.; Dickinson, J.S. | 2001-42 | | ***TADCR035*** | ***TRT00001005*** | KC206737 | KC206845 | KC206934 | KC206776 | KC206632 | KC207008 | KC206921 | KC207049 | KC206594 | KC174293 | KC206558 | KC173392 | Table S1 | Table S1 |  | |
|  |  | *4x* | Talent, N.;  Hirst, G. | NT373 | | ***TADCR235*** | ***TRT00000938*** | KC206734 | KC206864 | KC206952 | KC206813 | KC206653 | KC206985 | KC206898 | KC207026 | KC206612 | KC174314 | KC206582 | Table S1 | Table S1 | Table S1 |  | |
|  | *C. saligna* Greene | | |  | |  |  |  |  |  |  |  |  |  |  |  |  |  |  |  |  |  | |
|  |  | *2x+* | Dickinson, T.A. | 2004-05 | | ***TADCR120*** | ***TRT00001047*** | KC206742 | KC206846 | KC206935 | KC206778 | KC206633 | KC207009 | KC206922 | KC207050 | KC206595 | KC174294 | KC206559 | Table S1 | Table S1 | Table S1 |  | |
|  | ***Douglasianae*** (Loudon) Poletiko (H) | | | | | |  |  |  |  |  |  |  |  |  |  |  |  |  |  |  |  | |
|  | *C. castlegarensis* J.B. Phipps & O’Kennon | | | | | |  |  |  |  |  |  |  |  |  |  |  |  |  |  |  |  | |
|  |  | *4x* | Love, R.M. | C-2003-23F | | ***TADCR042*** | ***TRT00001063*** | KC206674 | KC206838 | KC206927 | KC206764 | KC206624 | KC207001 | KC206914 | KC207042 | KC206589 | KC174288 | KC206552 | KC173389 | Table S1 | Table S1 |  | |
|  | *C. douglasii* Lindl. | | |  | |  |  |  |  |  |  |  |  |  |  |  |  |  |  |  |  |  | |
|  |  | *4x* | Dickinson, T.A.; Coughlan, J.; Zarrei, M. | 2010-36 | |  | TRT00021087 | KC206700 | KC206855 | KC206944 | KC206797 | KC206644 | KC206976 | KC206889 | KC207020 | KC206605 | KC174304 | KC206580 | KC173397 | Table S1 | KC207059 |  | |
|  |  |  | 11-Jun-2011, BC, Kitimat-Stikine R. D. (55.098928, -128.076194) | | | | |  |  |  |  |  |  |  |  |  |  |  |  |  |  |  | |
|  |  | *4x* | Coughlan, J.; Shaw, C.; Zarrei, M. | JC387 | | ***TADCR244*** | ***TRT00020213*** | KC206709 | KC206868 | KC206956 | KC206820 | KC206657 | KC206989 | KC206902 | KC207030 | KC206616 | KC174318 | - | Table S1 | Table S1 | Table S1 |  | |
|  |  | *4x* | Coughlan, J.; Shaw, C.; Zarrei, M. | JC385 | | ***TADCR245*** | ***TRT00020211*** | KC206710 | KC206869 | KC206957 | KC206821 | KC206658 | KC206990 | KC206903 | KC207031 | - | KC174319 | KC206567 | Table S1 | Table S1 | Table S1 |  | |
|  |  | *5x* | Dickinson, T.A.; Coughlan, J.; Zarrei, M. | 2010-38 | | ***TADCR200*** | ***TRT00002612*** | KC206699 | KC206856 | KC206945 | KC206798 | KC206645 | KC206977 | KC206890 | KC207021 | KC206622 | KC174305 | KC206581 | Table S1 | Table S1 | Table S1 |  | |
|  | *C. enderbyensis* J.B. Phipps & O’Kennon | | | | | |  |  |  |  |  |  |  |  |  |  |  |  |  |  |  |  | |
|  |  | *4x* | *Zika* | 18445 | | ***TADCR152*** | ***TRT00004465*** | KC206712 | KC206862 | KC206950 | KC206809 | KC206651 | KC206983 | KC206896 | KC207024 |  | KC174312 | KC206566 | Table S1 | Table S1 | Table S1 |  | |
|  | *C. gaylussacia* A. Heller | | |  | |  |  |  |  |  |  |  |  |  |  |  |  |  |  |  |  |  | |
|  |  | *3x* | Shiller, J.; Tusha, J.; Dickinson, T.A.; Heckel, M. | PORE-509-1 | | ***TADCR239*** | ***TRT00002016*** | KC206713 | KC206863 | KC206951 | KC206810 | KC206652 | KC206984 | KC206897 | KC207025 | KC206611 | KC174313 | KC206584 | Table S1 | Table S1 | Table S1 |  | |
|  | *C. okennoni* J.B. Phipps | | |  | |  |  |  |  |  |  |  |  |  |  |  |  |  |  |  |  |  | |
|  |  | *4x* | Lo, E.Y.Y.; Dickinson, T.A. | EL-152 | | ***TADCR172*** | ***TRT00001550*** | KC206683 | KC206843 | KC206932 | KC206768 | KC206629 | KC207006 | KC206919 | KC207047 | KC206592 | KC174290 | KC206556 | Table S1 | Table S1 | Table S1 |  | |
|  | *C. shuswapensis* J.B. Phipps & O’Kennon | | | | | |  |  |  |  |  |  |  |  |  |  |  |  |  |  |  |  | |
|  |  | *4x* | Dickinson, T.A.; Dickson, E.E.; Dickinson, A.K. | 2007-13 | | ***TADCR168*** | ***TRT00003557*** | KC206749 | KC206876 | KC206964 | KC206829 | KC206665 | KC206997 | KC206910 | KC207038 | - | KC174326 | KC206574 | KC173403 | Table S1 | Table S1 |  | |
|  |  | | |  | |  |  |  |  |  |  |  |  |  |  |  |  |  |  |  |  |  | |
|  | *C. suksdorfii* (Sarg.) Kruschke | | |  | |  |  |  |  |  |  |  |  |  |  |  |  |  |  |  |  |  | |
|  |  | *2x* | Zika, P. | 18485 | | ***TADCR146*** | ***TRT00003669*** | KC206690 | KC206849 | KC206938 | KC206784 | KC206636 | KC206968 | KC206881 | KC207013 | KC206598 | KC174297 | KC206560 | KC173394 | Table S1 | Table S1 |  | |
|  |  | *3x* | Lo, E.Y.Y.; Dickinson, T.A.; Nguyen, S. | EL-172 | | ***TADCR178*** | ***TRT00001606*** | KC206691 | KC206850 | KC206939 | KC206785 | KC206637 | KC206969 | KC206882 | KC207014 | KC206599 | KC174298 | - | Table S1 | Table S1 | Table S1 |  | |
|  |  | *3x* | Dickinson, T.A.; Coughlan, J.; Zarrei, M. | 2010-42 | |  | TRT00002616 | KC206706 | KC206867 | KC206955 | KC206819 | KC206656 | KC206988 | KC206901 | KC207029 | KC206615 | KC174317 | KC206586 | Table S1 | Table S1 | Table S1 |  | |
|  |  |  | 17-Aug-2010, BC, Skeena-Queen Charlotte R. D., (53.250378, -132.120414) | | | | | |  |  |  |  |  |  |  |  |  |  |  |  |  |  | |
|  |  | 4*x* | Lo, E.Y.Y.; Dickinson, T.A.; Nguyen, S. | EL-30 | | ***TADCR002*** | ***TRT00001581*** | KC206687 | KC206848 | KC206937 | KC206781 | KC206635 | KC207011 | KC206924 | KC207052 | KC206597 | KC174296 | - | Table S1 | Table S1 | Table S1 |  | |
|  | ***Purpureofructus*** J.B. Phipps & O’Kennon (E) | | | | | |  |  |  |  |  |  |  |  |  |  |  |  |  |  |  |  | |
|  | *C. aquacervensis* J.B. Phipps & O’Kennon | | | | | |  |  |  |  |  |  |  |  |  |  |  |  |  |  |  |  | |
|  |  | *4x* | Dickinson, T.A.; Dickson, E.E.; Dickinson, A.K. | 2007-12 | | ***TADCR162*** | ***TRT00004665*** | KC206756 | KC206873 | KC206961 | KC206832 | KC206662 | KC206994 | KC206907 | KC207035 | - | KC174323 | KC206571 | Table S1 | Table S1 | KC207064 |  | |
|  | *C. atrovirens* J.B. Phipps & O’Kennon | | | | | |  |  |  |  |  |  |  |  |  |  |  |  |  |  |  |  | |
|  |  | *-* | Phipps, J.B. | *8344* | |  | TRT00004309 | KC206758 | KC206871 | KC206959 | KC206828 | KC206660 | KC206992 | KC206905 | KC207033 | - | KC174321 | KC206569 | KC173401 | Table S1 | Table S1 |  | |
|  |  |  | 16-May-2002, BC, North Okanagan R. D. (50.585842, -119.130659) | | | | |  |  |  |  |  |  |  |  |  |  |  |  |  |  |  | |
|  | *C. okanaganensis* J.B. Phipps & O’Kennon | | | | | |  |  |  |  |  |  |  |  |  |  |  |  |  |  |  |  | |
|  |  | *4x* | *Dickinson, Lee, and Talent* | 2008-46 | | ***TADCR240*** | ***TRT00002484*** | KC206718 | KC206865 | KC206953 | KC206816 | KC206654 | KC206986 | KC206899 | KC207027 | KC206613 | KC174315 | KC206583 | Table S1 | Table S1 | Table S1 |  | |
|  | *C. orbicularis* J.B. Phipps & O’Kennon | | | | | |  |  |  |  |  |  |  |  |  |  |  |  |  |  |  |  | |
|  |  | *-* | Phipps, J.B. | *8365* | |  | TRT00004480 | KC206757 | KC206872 | KC206960 | KC206824 | KC206661 | KC206993 | KC206906 | KC207034 | KC206618 | KC174322 | KC206570 | KC173402 | Table S1 | KC207063 |  | |
|  |  |  | 18-May-2002, BC, North Okanagan R. D. (50.471476, -119.150475) | | | | |  |  |  |  |  |  |  |  |  |  |  |  |  |  |  | |
|  | *C. phippsii* O’Kennon | | |  | |  |  |  |  |  |  |  |  |  |  |  |  |  |  |  |  |  | |
|  |  | - | Coughlan, J.; Shaw, C.; Zarrei, M. | JC178 | | ***TADCR246*** | ***TRT00020265*** | KC206755 | KC206870 | KC206958 | KC206825 | KC206659 | KC206991 | KC206904 | KC207032 | KC206617 | KC174320 | KC206568 | Table S1 | Table S1 | Table S1 |  | |
|  | *C. cupressocollina* J.B. Phipps & O’Kennon | | | | | |  |  |  |  |  |  |  |  |  |  |  |  |  |  |  |  | |
|  |  | *4x* | Dickinson, T.A.; Dickson, E.E.; Dickinson, A.K. | 2007-20 | | ***TADCR163*** | ***TRT00004547*** | KC206752 | KC206874 | KC206962 | KC206830 | KC206663 | KC206995 | KC206908 | KC207036 | KC206619 | KC174324 | KC206572 | Table S1 | Table S1 | Table S1 |  | |
|  | ***Montaninsulae*** J.B. Phipps & O’Kennon (E) | | | | | |  |  |  |  |  |  |  |  |  |  |  |  |  |  |  |  | |
|  | *C. rivuloadamensis* J.B. Phipps & O’Kennon | | | | | |  |  |  |  |  |  |  |  |  |  |  |  |  |  |  |  | |
|  |  | *4x* | Dickinson, T.A.; Dickson, E.E.; Dickinson, A.K. | 2007-13 | | ***TADCR168*** | ***TRT00003557*** | KC206749 | KC206876 | KC206964 | KC206829 | KC206665 | KC206997 | KC206910 | KC207038 | - | KC174326 | KC206574 | KC173403 | Table S1 | Table S1 |  | |
|  |  | | | | | |  |  |  |  |  |  |  |  |  |  |  |  |  |  |  |  | |
|  | *C. rivulopugnensis* J.B. Phipps & O’Kennon | | | | | |  |  |  |  |  |  |  |  |  |  |  |  |  |  |  |  | |
|  |  | *4x* | Dickinson, T.A.; Dickson, E.E.; Dickinson, A.K. | 2007-21 | | ***TADCR154*** | ***TRT00003524*** | KC206748 | KC206875 | KC206963 | KC206831 | KC206664 | KC206996 | KC206909 | KC207037 | KC206620 | KC174325 | KC206573 | Table S1 | Table S1 | Table S1 |  | |
| E. ***Sanguineae*** Zabel ex C.K. Schneid. (G1, G2) | | | | | | |  |  |  |  |  |  |  |  |  |  |  |  |  |  |  |  | |
|  | ***Nigrae*** (Loudon) Russanov | | |  | |  |  |  |  |  |  |  |  |  |  |  |  |  |  |  |  |  | |
|  | *C. maximowiczii* C.K. Schneid. | | |  | |  |  |  |  |  |  |  |  |  |  |  |  |  |  |  |  |  | |
|  |  | *-* | Romankova, T. | 4 | | ***TADCR094*** | ***TRT00002370*** | KC206682 | KC206841 | KC206930 | KC206773 | KC206627 | KC207004 | KC206917 | KC207045 | - | - | - | Table S1 | Table S1 | Table S1 |  | |
|  | *C. nigra* Waldst. & Kit. | | |  | |  |  |  |  |  |  |  |  |  |  |  |  |  |  |  |  |  | |
|  |  | *2x+* | Christensen, K.I. | 294 | | ***TADCR036*** | ***TRT00002052*** | KC206719 | KC206842 | KC206931 | KC206774 | KC206628 | KC207005 | KC206918 | KC207046 | - | - | KC206555 | KC173391 | Table S1 | Table S1 |  | |
|  | ***Sanguineae*** (Zabel ex C.K. Schneid.) Rehder | | | | | |  |  |  |  |  |  |  |  |  |  |  |  |  |  |  |  | |
|  | *C. wilsonii* Sarg. | | |  | |  |  |  |  |  |  |  |  |  |  |  |  |  |  |  |  |  | |
|  |  | *2x* | Dickinson, T.A. | AA749-74A | | ***TADCR114*** | ***TRT00002055*** | KC206732 | KC206851 | KC206940 | KC206787 | KC206638 | KC206970 | KC206883 | KC207015 | KC206600 | KC174299 | - | KC173395 | Table S1 | KC207056 |  | |
|  |  |  |  |  | |  |  |  |  |  |  |  |  |  |  |  |  |  |  |  |  |  | |
| **OUTGROUPS** | | | |  | |  |  |  |  |  |  |  |  |  |  |  |  |  |  |  |  |  | |
|  |  | *Amelanchier alnifolia* ( Nutt. ) Nutt. ex M.Roem. | | | | |  |  |  |  |  |  |  |  |  |  |  |  |  |  |  |  | |
|  |  | *4x?* | Coughlan, J.; Shaw, C.; Zarrei, M. | JC431 | |  | TRT00021066 | KC206745 | KC206880 | KC206967 | KC206800 | KC206646 | KC207012 | KC206925 | KC207055 | KC206606 | KC174306 | KC206587 | CCDB | CCDB | CCDB |  | |
|  |  |  |  | 11-Jun-2011, BC, Cariboo R.D. (54.06299, -124.61882) | | | | |  |  |  |  |  |  |  |  |  |  |  |  |  |  | |
|  |  | *Cotoneaster taoensis* G. Klotz | | | | |  |  |  |  |  |  |  |  |  |  |  |  |  |  |  |  | |
|  |  |  | Lo & Donoghue (2012) | | | | | JQ391914 | JQ392094 | JQ390794 | JQ392203 | JQ391132 | JQ391633 | JQ391460 | JQ391755 | - | - | - | JQ390963 | JQ391287 | JQ390687 |  | |
|  |  | *Malus angustifolia* (Aiton) Michx. | | | | |  |  |  |  |  |  |  |  |  |  |  |  |  |  |  |  | |
|  |  |  | Lo & Donoghue (2012) except as noted | | | | | EF127116^1^ | EF127190^1^ | EF127227^1^ | AM157404^2^ | JQ391189 | JQ391661 | JQ391529 | - | - | - | - | JQ391013 | JQ391358 | JQ390726 |  | |
|  |  | *Pyrus pyrifolia* (Burm.f.) Nakai | | | | |  |  |  |  |  |  |  |  |  |  |  |  |  |  |  |  | |
|  |  |  | Lo & Donoghue (2012) except as noted | | | | | AP012207^3^ | AP012207^3^ | AP012207^3^ | AP012207^3^ | JQ391214 | JQ391698 | JQ391542 | JQ391854 | JX122473^4^ | GU363851^5^ | AP012207^3^ | JQ391048 | JQ391390 | JQ390748 |  | |
