# Supplementary material for: DNA barcodes from four loci provide poor resolution of taxonomic groups in the genus Crataegus
Source: AoB Plants. 2015 Apr 29;7:plv045. doi: 10.1093/aobpla/plv045 (PMC4480070; doi:10.1093/aobpla/plv045)
Supplement: Additional Information [file supp_plv045_plv045supp_table3.doc]

| Table S3. *Crataegus* voucher information and GenBank accession numbers for the AT1 region used in the current study. Asterisks, total genomic DNA extracted from seed rather than leaf tissue. Code, DNA extraction code used in Fig. S3. Data for vouchers with TRT numbers in bolded italics are found in Table S1. | | | | | | |
| --- | --- | --- | --- | --- | --- | --- |
|  | |  |  |  |  |  |
| **Section, species (ploidy level)** | | | **Voucher information** | **TRT accession** | **Code** | **GenBank Acession numbers** |
| *Mespilus* | | |  |  |  |  |
|  | *C. germanica* (*2x*) | | *Dickinson* 2000-54 | ***TRT00026649*** | 53 | KC172979, KC172980, KC172981, KC172982, KC172983, KC172984, KC172985, KC172986 |
|  | *C. germanica* (*2x*) | | AAD11600 | voucher in HUB | 56 | KC172967, KC172968, KC172969, KC172970, KC172971, KC172972, KC172973, KC172974, KC172975, KC172976, KC172977, KC172978 |
| *Brevispinae* | | |  |  |  |  |
|  | *C. brachyacantha* (*2x*) | | *Dickinson, Talent, and Nguyen* 2003-32 | ***TRT00000023*** | 25 | KC172955, KC172956, KC172957, KC172958 |
|  | *C. brachyacantha* (*2x*) | | *Reid* 5206 | ***TRT00000027*** | 22 | KC172951, KC172952, KC172953, KC172954 |
| *Crataegus* | | |  |  |  |  |
|  | *C. monogyna* (*2x*) | | Dickinson, Lee, and Talent 2008-26 | ***TRT00002452*** | 162 | KC172987, KC172988, KC172989, KC172990, KC172991, KC172992, KC172993, KC172994, KC172995 |
| *Coccitaegus* | | |  |  |  |  |
|  | *C. × ninae-celottiae (2x)* | | *Purich and Talent* MP85 | ***TRT00002250*** | 154 | KC173383, KC173384, KC173385, KC173386, KC173387, KC173388 |
| *Coccineae* | | |  |  |  |  |
|  | *C. crus-galli* (*2x*) | | *Talent* NT213a | ***TRT00019161*** | 38 | KC173308, KC173309 |
|  | *C. crus-galli* (2x) | | *Talent* NT283 |  | 40 | KC172959, KC172960, KC172961, KC172962, KC172963, KC172964, KC172965, KC172966 |
|  | *C. punctata* (*2x*)* | | Dickinson 897  ON, Middlesex Co. (42.72798, -81.66808) | Seed collection; sight identification | 119 | KC173322, KC173323, KC173324, KC173325, KC173326, KC173327, KC173328 |
|  | *C. punctata* var. aurea (*2x*)* | | Dickinson 1496  ON, York R.M. (44.01576, -79.65465) | Seed collection; sight identification | 118 | KC173311, KC173312, KC173313, KC173314, KC173315, KC173316, KC173317, KC173318, KC173319, KC173320, KC173321 |
|  | *C. chrysocarpa* (*4x*) | | *Talent, Heckel, and Lee* NT587 | ***TRT00002699*** | 124 | KC173354, KC173355, KC173356, KC173357, KC173358, KC173359, KC173360, KC173361, KC173362, KC173363, KC173364, KC173365, KC173366 |
|  | *C. chrysocarpa* var. *piperi (4x)* | | *Love s.n.* | ***TRT00018678*** | 121 | KC173329, KC173330, KC173331, KC173332, KC173333, KC173334 |
| *Macracanthae* | | |  |  |  |  |
|  | *C. macracantha (4x)* | | *Talent* NT224 | ***TRT00018679*** | 122 | KC173335, KC173336, KC173337, KC173338, KC173339 |
|  | *C. macracantha* *(4x*) | | *Talent* NT440 | ***TRT00018268*** | 123 | KC173340, KC173341, KC173342, KC173343, KC173344, KC173345, KC173346, KC173347, KC173348, KC173349, KC173350, KC173351, KC173352, KC173353 |
| *Crataeglasia* | | |  |  |  |  |
|  | *C. × cogswellii* (2x) | | *Lo*, *Dickinson, and Nguyen* EL-85 | ***TRT00002654*** | 160 | KC173305, KC173306, KC173307 |
| *Douglasia* | | |  |  |  |  |
|  | *C. rivularis* (*4x*) | | *Dickinson, Gervais, and Dickinson* 2001-43 | ***TRT00001006*** | 81 | KC173193, KC173194 |
|  | *C. saligna (2x)* | | *Dickinson* 2004-05 | ***TRT00001047*** | 90 | KC173001, KC173002, KC173003, KC173004, KC173005, KC173006, KC173007, KC173008, KC173009, KC173010, KC173011, KC173012, KC173013 |
|  | *C. saligna* (2x) | | *Talent and Hirs*t NT368 | ***TRT00001018*** | 92 | KC173014, KC173015, KC173016, KC173017, KC173018, KC173019, KC173020, KC173021, KC173022 |
|  | *C. saligna* (*2x*) | | *Talent and Hirs*t NT369 | ***TRT00001022*** | 93 | KC173023, KC173024, KC173025, KC173026, KC173027, KC173028 |
|  | *C. castlegarensis*(*4x*) | | *Love* C-2003-23F | ***TRT00001067*** | 32 | KC173115, KC173116, KC173117, KC173118, KC173119, KC173120, KC173121, KC173122, KC173123, KC173124, KC173125 |
|  | *C. douglasii* (*4x*) | | *Love* C-2003-23F |  | 32 | KC173176, KC173177, KC173178 |
|  | *C. douglasii* (*4x*) | | *Dickinson, Coughlan, and Zarrei* 2010-28 | ***TRT00002602*** | 143 | KC173163, KC173164, KC173165, KC173166, KC173167, KC173168, KC173169, KC173170, KC173171, KC173172, KC173173, KC173174, KC173175 |
|  | *C. douglasii (4x)* | | *Dickinson, Coughlan, and Zarrei* 2010-36 | TRT00021087 (Table S2) | 147 | KC173367, KC173368, KC173369, KC173370, KC173371, KC173372, KC173373, KC173374, KC173375, KC173376, KC173377, KC173378, KC173379, KC173380, KC173381, KC173382 |
|  | *C. douglasii* (*4x*) | | *Lo, Dickinson, and Nguyen* EL-121  ID, Nez Perce Co. (46.866667, -116.71667) | TRT00001250 | 125 | KC173149, KC173150, KC173151, KC173152, KC173153, KC173154, KC173155, KC173156, KC173157, KC173158, KC173159, KC173160, KC173161, KC173162 |
|  | *C. douglasii* (*4x*)* | | *Dickinson, Coughlan, and Zarrei* 2010-56  (= NT503) BC, Columbia Shuswap R. D. (50.8758, -118.9221) | TRT00002626 | 115 | KC173126, KC173127, KC173128, KC173129, KC173130, KC173131, KC173132, KC173133, KC173134, KC173135, KC173136, KC173137, KC173138, KC173139, KC173140, KC173141, KC173142, KC173143, KC173144, KC173145, KC173146, KC173147, KC173148 |
|  | *C. douglasii* (*4x*) | | *Zika* 18453 | ***TRT00003801*** | 45 | KC173280, KC173281, KC173282, KC173283, KC173284, KC173285, KC173286, KC173287, KC173288, KC173289, KC173290 |
|  | *C. douglasii* (5*x)* | | *Dickinson, Coughlan, and Zarrei* 2010-31 | ***TRT00002605*** | 145 | KC173241, KC173242, KC173243, KC173244, KC173245, KC173246, KC173247, KC173248, KC173249, KC173250, KC173251, KC173252, KC173253, KC173254, KC173255, KC173256, KC173257, KC173258, KC173259, KC173260, KC173261, KC173262, KC173263 |
|  | *C. douglasii* (*5x*) | | *Dickinson, Coughlan, and Zarrei* 2010-38 | ***TRT00002612*** | 148 | KC173264, KC173265, KC173266, KC173267, KC173268, KC173269, KC173270, KC173271, KC173272, KC173273, KC173274, KC173275, KC173276, KC173277, KC173278, KC173279 |
|  | *C. gaylusssacia* (*3x*) | | *Lo, Dickinson, Nguyen, and Love* EL-57  OR, Lane Co. (43.7378, -122.6271) | TRT00001740 | 98 | KC173057, K 173058, KC173059, KC173060, KC173061, KC173062, KC173063, KC173064, KC173065, KC173066, KC173067, KC173068, KC173069, KC173070, KC173071, KC173072, KC173073, KC173074 |
|  | *C. okennonii* (*4x*) | | *Lo and Dickinson* EL-153 | ***TRT00001549*** | 73 | KC173179, KC173180, KC173181, KC173182, KC173183, KC173184, KC173185, KC173186, KC173187, KC173188, KC173189, KC173190, KC173191, KC173192 |
|  | *C. suksdorfii* (*2x*) | | *Dickinson and Lo* 2006-19 | ***TRT00001569*** | 107 | KC173029, KC173030, KC173031, KC173032, KC173033, KC173034, KC173035, KC173036 |
|  | *C. suksdorfii* (*2x*) | | *Lo and Dickinson* 2006-22 | ***TRT00001563*** | 108 | KC173037, KC173038, KC173039, KC173040, KC173041, KC173042, KC173043, KC173044 |
|  | *C. suksdorfii* (*2x*) | | *Lo, Dickinson, and Nguyen* EL-70  OR, Linn Co. (44.33444, -123.12278) | TRT00002652 | 127 | KC173297, KC173298, KC173299, KC173300 |
|  | *C. suksdorfii* (*2x*) | | *Coughlan, Shaw, and Zarrei* JC094 | ***TRT00020157*** | 137 | KC172927, KC172928, KC172929, KC172930, KC172931, KC172932, KC172933, KC172934, KC172935, KC172936, KC172937, KC172938 |
|  | *C. suksdorfii* (*2x*) | | *Coughlan, Shaw, and Zarrei* JC097  OR, Multnomah Co. (45.5524, -122.3606) | TRT00020367 | 138 | KC172939, KC172940, KC172941, KC172942, KC172943, KC172944, KC172945 |
|  | *C. suksdorfii* (*2x*) | | *Coughlan, Shaw, and Zarrei* JC116  OR, Columbia Co. (45.73203, -122.76713) | TRT00020384 | 139 | KC172946, KC172947, KC172948, KC172949, KC172950 |
|  | *C. suksdorfii (2x)* | | *Zika* 18485 (=18430, 18417) | ***TRT00003669*** | 101 | KC173291, KC173292, KC173293, KC173294, KC173295, KC173296 |
|  | *C. suksdorfii* (*3x*) | | *Lo, Dickinson, Nguyen, and Love* EL-65 | ***TRT00001760*** | 100 | KC173075, KC173076, KC173077, KC173078, KC173079, KC173080, KC173081, KC173082, KC173083, KC173084 |
|  | *C. suksdorfii* (*3x*) | | *Lo, Dickinson, and Nguyen* EL-165 | ***TRT00001605*** | 102 | KC173085, KC173086, KC173087, KC173088, KC173089, KC173090, KC173091 |
|  | *C. suksdorfii* (*3x*) | | *Lo, Dickinson, and Nguyen* EL-173 | ***TRT00001602*** | 103 | KC173092, KC173093, KC173094, KC173095, KC173096, KC173097, KC173098, KC173099, KC173100, KC173101, KC173102, KC173103, KC173104 |
|  | *C. suksdorfii* (*3x*) | | *Lo, Dickinson, and Nguyen* EL-188 | ***TRT00001640*** | 105 | KC173105, KC173106, KC173107, KC173108, KC173109, KC173110, KC173111, KC173112, KC173113, KC173114 |
|  | *C. suksdorfii* (*4x*) | | *Lo, Dickinson, and Nguyen* EL-30 | ***TRT00001581*** | 96 | KC173205, KC173206, KC173207, KC173208, KC173209, KC173210, KC173211, KC173212, KC173213, KC173214, KC173215, KC173216, KC173217, KC173218, KC173219, KC173220, KC173221, KC173222, KC173223, KC173224, KC173225, KC173226, KC173227, KC173228, KC173229, KC173230 |
|  | *C. suksdorfii* (*4x*) | | *Lo and Dickinson* EL-45 | ***TRT00001577*** | 97 | KC173231, KC173232, KC173233, KC173234, KC173235, KC173236, KC173237, KC173238, KC173239, KC173240 |
|  | *C. suksdorfii* (*4x*) | | *Lo, Dickinson, and Nguyen* EL-36 | ***TRT00001583*** | 106 | KC173195, KC173196, KC173197, KC173198, KC173199, KC173200, KC173201, KC173202, KC173203, KC173204 |
| *Sanguineae* | | |  |  |  |  |
|  | *C. maximoviczii* (*2x*) | | *Romankova* 4 | ***TRT00002370*** | 64 | KC173310 |
|  | *C. nigra* (*2x*) | | *Christensen* KIC 294 | ***TRT00002052*** | 71 | KC172996, KC172997, KC172998, KC172999, KC173000 |
|  | *C. wilsonii (2x)* | | *Dickinson* AA749-74A | ***TRT00002055*** | 111 | KC173045, KC173046, KC173047, KC173048, KC173049, KC173050, KC173051, KC173052, KC173053, KC173054, KC173055, KC173056 |
| OUTGROUP | | |  |  |  |  |
|  | *Amelanchier alnifolia (3x)* | | *Coughlan, Shaw, and Zarrei* JC431 | TRT00021066 | 143 | KC173301, KC173302, KC173303, KC173304 |
