# Supplementary material for: DNA barcodes from four loci provide poor resolution of taxonomic groups in the genus Crataegus
Source: AoB Plants. 2015 Apr 29;7:plv045. doi: 10.1093/aobpla/plv045 (PMC4480070; doi:10.1093/aobpla/plv045)
Supplement: Additional Information [file supp_plv045_plv045supp_table4.doc]

| Table S4. *Crataegus* voucher information and GenBank accession numbers for the PEPC region used in the current study. Asterisks, total genomic DNA extracted from seed rather than leaf tissue. Code, DNA extraction code used in Fig. S4. Data for vouchers with TRT numbers in bolded italics are found in Table S1. | | | | | | |
| --- | --- | --- | --- | --- | --- | --- |
|  | |  |  |  |  |  |
| **Section, species**  **(ploidy level)** | | | **voucher** | **TRT accession** | **Code** | **GenBank accession numbers**  **(L= Large-copy; S= Short-copy)** |
| *Mespilus* | | |  |  |  |  |
|  | *C. germanica* (2x) | | *Dickinson* 2000-54 | ***TRT00026649*** | 53 | KC203811 (L), KC203812 (L), KC203813 (L), KC203814 (L), KC203815 (L), KC203816 (L), KC203817 (L), KC203818 (L), KC203881 (S) |
| *Brevispinae* | | |  |  |  |  |
|  | | *C. brachyacantha* (*2x*) | *Reid* 5202 | ***TRT00000025*** | 20 | KC203668 (L), KC203669 (L), KC203670 (L) |
|  | | *C. brachyacantha* (*2x*) | *Reid* 5203 | ***TRT00000028*** | 24 | KC203676 (L), KC203677 (L), KC203678 (L), KC203679 (L), KC203680 (L), KC203681 (L), KC203682 (L), KC203683 (L) |
|  | | *C. brachyacantha* (*2x*) | *Reid* 5206 | ***TRT00000027*** | 22 | KC203671 (L), KC203672 (L), KC203673 (L), KC203674 (L), KC203675 (L), KC203866 (S) |
|  | | *C. brachyacantha* (*2x*) | *Dickinson, Talent, and Nguyen* 2003-32 | ***TRT00000023*** | 25 | KC203684 (L), KC203685 (L), KC203686 (L), KC203687 (L), KC203688 (L), KC203689 (L), KC203690 (L), KC203691 (L), KC203867 (S) |
| *Crataegus* | | |  |  |  |  |
|  | | *C. pentagyna* (2x) | *Christensen s.n.* 315-1993 | ***TRT00001886, TRT00001887, TRT00001888*** | ***128*** | KC203643 (L), KC203644 (L), KC203645 (L), KC203646 (L), KC203699 (L), KC203700 (L), KC203701 (L), KC203702 (L), KC203832 (S), KC203874 (S), KC203875 (S) |
| *Coccitaegus* | | |  |  |  |  |
|  | | *C. × ninae-celottiae (2x)* | *Purich and Talent* MP84 | ***TRT00002249*** | 157 | KC203859 (S), KC203860 (S), KC203861 (S) |
|  | | *C. × ninae-celottiae (2x)* | *Purich and Talent* MP85 | ***TRT00002250*** | 154 | KC203658 (L), KC203659 (L), KC203660 (L), KC203661 (L), KC203662 (L), KC203663 (L), KC203664 (L), KC203665 (L), KC203857 (S), KC203858 (S) |
|  | | |  |  |  |  |
| *Coccineae* | | |  |  |  |  |
|  | | *C. crus-galli* (*2x*) | *Talent* NT213a | ***TRT00019161*** | 38 | KC203692 (L), KC203868 (S), KC203869 (S), KC203870 (S), KC203871 (S), KC203872 (S), KC203873 (S) |
|  | | *C. crus-galli* (2x) | *Talent* NT213a | ***TRT00019161*** | 38 | KC203693 (L), KC203694 (L), KC203695 (L) |
|  | | *C. crus-galli* (2x) | *Talent* NT283 |  | 40 | KC203696 (L), KC203697 (L), KC203698 (L) |
|  | | *C. punctata* (2x)* | *Dickinson* 897  ON, Middlesex Co. (42.72798, -81.66808) | Seed collection; sight identification | 119 | KC203627 (L), KC203628 (L), KC203629 (L), KC203630 (L), KC203631 (L), KC203708 (L), KC203819 (S) |
|  | | *C. punctata* var. aurea (*2x*)* | *Dickinson* 1496  ON, York R.M. (44.01576, -79.65465) | Seed collection; sight identification | 118 | KC203703 (L), KC203704 (L), KC203705 (L), KC203706 (L), KC203707 (L) |
|  | | *C. chrysocarpa* (*4x*) | *Talent, Heckel, and Lee* NT587 | ***TRT00002699*** | 124 | KC203637 (L), KC203638 (L), KC203639 (L), KC203640 (L), KC203641 (L), KC203824 (S), KC203825 (S), KC203826 (S), KC203827 (S) |
| *Macracanthae* | | |  |  |  |  |
|  | | *C. macracantha (4x)* | *Talent* NT224 | ***TRT00018679*** | 122 | KC203632 (L), KC203633 (L), KC203823 (S) |
|  | | *C. macracantha* (4x) | *Talent* NT440 |  | 123 | KC203634 (L), KC203635 (L), KC203636 (L) |
| *Crataeglasia* | | |  |  |  |  |
|  | | *C. × cogswellii* (2x) | *Lo*, *Dickinson, and Nguyen* EL-85 | ***TRT00002654*** | 160 | KC203666 (L), KC203667 (L), KC203862 (S) |
| *Douglasia* | | |  |  |  |  |
|  | | *C. rivularis (4x)* | *Talent and Hirst* NT373 | ***TRT00000938*** | 180 | KC203863 (S), KC203864 (S), KC203865 (S) |
|  | | *C. saligna (2x)* | *Dickinson* 2004-05 | ***TRT00001047*** | 90 | KC203722 (L), KC203723 (L), KC203724 (L), KC203725 (L), KC203726 (L), KC203727 (L) |
|  | | *C. saligna* (2x) | *Dickinson* 2004-06 | ***TRT00001026*** | 89 | KC203715 (L), KC203716 (L), KC203717 (L), KC203718 (L), KC203719 (L), KC203720 (L), KC203721 (L), KC203878 (S) |
|  | | *C. saligna* (2x) | *Dickinson* 2004-08 | ***TRT00001024*** | 88 | KC203711 (L), KC203712 (L), KC203713 (L), KC203714 (L) |
|  | | *C. saligna* (2x) | *Talent and Hirs*t NT368 | ***TRT00001018*** | 92 | KC203737 (L), KC203738 (L), KC203739 (L), KC203740 (L), KC203741 (L), KC203742 (L) |
|  | | *C. saligna* (2x) | *Talent and Hirst* NT371 | ***TRT00001021*** | 91 | KC203728 (L), KC203729 (L), KC203730 (L), KC203731 (L), KC203732 (L), KC203733 (L), KC203734 (L), KC203735 (L), KC203736 (L), KC203709 (L), KC203710 (L), KC203876 (S), KC203877 (S) |
|  | | *C. douglasii* (4x)* | *Dickinson, Coughlan, and Zarrei* 2010-56  (= NT503) BC, Columbia Shuswap R. D. (50.8758, -118.9221) | TRT00002626 | 115 | KC203615 (L), KC203616 (L), KC203617 (L), KC203618 (L), KC203619 (L), KC203620 (L), KC203621 (L), KC203622 (L), KC203623 (L), KC203624 (L), KC203625 (L), KC203626 (L), KC203820 (S), KC203821 (S), KC203822 (S) |
|  | | *C. douglasii* (4x) | *Lo, Dickinson, and Nguyen* EL-121  ID, Nez Perce Co. (46.866667, -116.71667) | TRT00001250 | 125 | KC203642 (L), KC203828 (S), KC203829 (S), KC203830 (S), KC203831 (S) |
|  | | *C. douglasii* (4x) | *Dickinson, Coughlan, and Zarrei* 2010-28 | ***TRT00002602*** | 143 | KC203833 (S), KC203834 (S), KC203835 (S), KC203836 (S), KC203837 (S), KC203838 (S), KC203839 (S) |
|  | | *C. douglasii (4x)* | *Dickinson, Coughlan, and Zarrei* 2010-36 | TRT00021087 (Table S2) | 147 | KC203652 (L), KC203653 (L), KC203654 (L), KC203655 (L), KC203656 (L), KC203657 (L), KC203848 (S), KC203849 (S) |
|  | | *C.douglasii* (5*x)* | *Dickinson, Coughlan, and Zarrei* 2010-31 | ***TRT00002605*** | 145 | KC203647 (L), KC203648 (L), KC203649 (L), KC203650 (L), KC203651 (L), KC203840 (S), KC203841 (S), KC203842 (S), KC203843 (S), KC203844 (S), KC203845 (S), KC203846 (S), KC203847 (S) |
|  | | *C. suksdorfii* (2x) | *Dickinson and Lo* 2006-19 | ***TRT00001569*** | 107 | KC203749 (L), KC203750 (L), KC203751 (L), KC203752 (L), KC203753 (L) |
|  | | *C. suksdorfii* (2x) | *Dickinson and Lo* 2006-19 | ***TRT00001569*** | 107 | KC203756 (L) |
|  | | *C. suksdorfii* (2x) | *Lo and Dickinson* 2006-22 | ***TRT00001563*** | 108 | KC203754 (L), KC203755 (L), KC203880 (S) |
|  | | *C. suksdorfii* (2x) | *Lo, Dickinson, and Nguyen* EL-70  OR, Linn Co. (44.33444, -123.12278) | TRT00002652 | 127 | KC203757 (L), KC203758 (L), KC203759 (L), KC203760 (L), KC203761 (L), KC203762 (L), KC203763 (L) |
|  | | *C. suksdorfii* (2x) | *Lo, Dickinson, and Nguyen* EL-103 JC116  OR, Columbia Co. (45.73203, -122.76713) | TRT00001918 | 129 | KC203764 (L), KC203765 (L), KC203766 (L), KC203767 (L), KC203768 (L), KC203769 (L), KC203770 (L), KC203771 (L), KC203772 (L) |
|  | | *C. suksdorfii* (2x) | *Coughlan, Shaw, and Zarrei* JC090  OR, Hood River Co. (45.67147, -121.8856) | TRT00020362 | 135 | KC203773 (L), KC203774 (L), KC203775 (L), KC203776 (L), KC203777 (L) |
|  | | *C. suksdorfii* (2x) | *Coughlan, Shaw, and Zarrei* JC092 | ***TRT00020153*** | 136 | KC203778 (L), KC203779 (L), KC203780 (L), KC203781 (L), KC203782 (L) |
|  | | *C. suksdorfii* (2x) | *Coughlan, Shaw, and Zarrei* JC094 | ***TRT00020157*** | 137 | KC203783 (L), KC203784 (L), KC203785 (L), KC203786 (L), KC203787 (L), KC203788 (L), KC203789 (L), KC203790 (L) |
|  | | *C. suksdorfii* (2x) | *Coughlan, Shaw, and Zarrei* JC097  OR, Multnomah Co. (45.5524, -122.3606) | TRT00020367 | 138 | KC203791 (L), KC203792 (L), KC203793 (L), KC203794 (L), KC203795 (L), KC203796 (L), KC203797 (L), KC203798 (L) |
|  | | *C. suksdorfii* (2x) | *Coughlan, Shaw, and Zarrei* JC116  OR, Columbia Co. (45.73203, -122.76713) | TRT00020384 | 139 | KC203799 (L), KC203800 (L), KC203801 (L), KC203802 (L), KC203803 (L), KC203804 (L), KC203805 (L) |
|  | | *C. suksdorfii* (2x) | *Coughlan, Shaw, and Zarrei* JC119 | ***TRT00020234*** | 140 | KC203806 (L), KC203807 (L), KC203808 (L), KC203809 (L), KC203810 (L) |
|  | | *C. suksdorfii (2x)* | *Zika* 18485 (=18430, 18417) | ***TRT00003669*** | ***101*** | KC203743 (L), KC203744 (L), KC203745 (L), KC203746 (L), KC203747 (L), KC203748 (L), KC203879 (S) |
| *Sanguineae* | | |  |  |  |  |
|  | | *C.maximoviczii* (*2x*) | *Romankova* 4 | ***TRT00002370*** | 64 | KC203882 (S), KC203883 (S) |
|  | | *C. nigra* (*2x*) | *Christensen* KIC 294 | ***TRT00002052*** | 71 | KC203884 (S), KC203885 (S) |
|  | | *C. wilsonii (2x)* | *Dickinson* AA749-74A | ***TRT00002055*** | 111 | KC203608 (L), KC203609 (L), KC203610 (L), KC203611 (L), KC203612 (L), KC203613 (L), KC203614 (L) |
| OUTGROUP | | |  |  |  |  |
|  | | *Amelanchier alnifolia (3x)* | *Coughlan, Shaw, and Zarrei* JC431 | TRT00021066 | 143 | KC203850 (S), KC203851 (S), KC203852 (S), KC203853 (S), KC203854 (S), KC203855 (S), KC203856 (S) |
|  | |  |  |  |  |  |
