# Supplementary material for: DNA barcodes from four loci provide poor resolution of taxonomic groups in the genus Crataegus
Source: AoB Plants. 2015 Apr 29;7:plv045. doi: 10.1093/aobpla/plv045 (PMC4480070; doi:10.1093/aobpla/plv045)
Supplement: Additional Information [file supp_plv045_plv045supp_table5.doc]

Table S5. Models of nucleotide evolution in *Crataegus* selected using the Akaike information criterion (AIC) for analyses of plastid (Fig. 1; Table S2) and nuclear (Fig. S3, S4; Table S3, S4) markers. Asterisks indicate loci that are protein-coding regions.

| **Markers** | **Model selected** | **Proportion of invariable sites** | **Gamma variation** |
| --- | --- | --- | --- |
| trnG-trnS | F81 | 0 | Equal rates for all sites |
| rpl2-trnH | GTR+G | 0 | 0.1373 |
| rpl20-rps12 | F81+I | 0.8580 | Equal rates for all sites |
| trnL-trnF | GTR+G | 0 | 0.2937 |
| atpB-rbcL | F81 | 0 | Equal rates for all sites |
| rps16 | GTR | 0 | Equal rates for all sites |
| rpl16 | GTR+I | 0.9115 | Equal rates for all sites |
| trnC-yfc6 | GTR+I | 0.7446 | Equal rates for all sites |
| accD | GTR+I | 0.5299 | Equal rates for all sites |
| rpoC1* | F81 | 0 | Equal rates for all sites |
| atpF-atpH | F81 | 0 | Equal rates for all sites |
| matK* | F81+I | 0.8552 | Equal rates for all sites |
| rbcLa* | HKY+I | 0.9096 | Equal rates for all sites |
| psbA-trnH | GTR+I | 0.5341 | Equal rates for all sites |
| Plastid-concatenated | GTR+I+G | 0.8467 | 0.8404 |
| PEPC | GTR+G | 0 | 1.1825 |
| AT1 | GTR+G | 0 | 0.9691 |
