# Supplementary material for: DNA barcodes from four loci provide poor resolution of taxonomic groups in the genus Crataegus
Source: AoB Plants. 2015 Apr 29;7:plv045. doi: 10.1093/aobpla/plv045 (PMC4480070; doi:10.1093/aobpla/plv045)
Supplement: Additional Information [file supp_plv045_plv045supp_table6.doc]

| Table S6. Comparison of *Crataegus* sequence variation between different markers utilized for the phylogenetic analyses (Fig. 1; Fig. S3, S4). * Minimum (mean ± Std Dev) Maximum; STR = safe taxonomic reduction; ^ outgroups excluded. | | | | | | | | | | | | |
| --- | --- | --- | --- | --- | --- | --- | --- | --- | --- | --- | --- | --- |
| **Matrices** | | **Sequences** | **Ungapped length of sequences*** | **Gapped length of sequences** | **% pairwise identity** | **% identical sites** | **% of parsimony informative sites** | **% GC**  **content** | **Nucleotide frequencies** | | | |
|  |  |  |  |  |  |  |  |  | **A C G T** | | | |
| **Plastid**  **loci** | accD^ | 31 | 608(662±26.7)751 | 782 | 94.6 | 73.9 | 1.41 | 21.2 | 0.362 | 0.131 | 0.119 | 0.388 |
|  | atpB-rbcL^ | 44 | 908(910.5±6.3)943 | 955 | 99.2 | 93.6 | 0.84 | 30.8 | 0.343 | 0.165 | 0.158 | 0.334 |
|  | atpF-atpH^ | 36 | 618(628.6±2.1)631 | 632 | 99.5 | 95.9 | 0.63 | 31.0 | 0.326 | 0.167 | 0.145 | 0.362 |
|  | **matK**^ | 38 | 535(760.1±51.1)793 | 793 | 99.5 | 96.7 | 0.88 | 32.5 | 0.297 | 0.169 | 0.158 | 0.376 |
|  | **rbcLa**^ | 32 | 552 | 552 | 99.8 | 98.4 | 0.36 | 42.9 | 0.269 | 0.199 | 0.23 | 0.302 |
|  | rpl2-trnH^ | 43 | 264(323.8±78)533 | 630 | 76.6 | 12.9 | 3.65 | 34.6 | 0.411 | 0.130 | 0.121 | 0.338 |
|  | rpl16^ | 44 | 1054(1073.6±11.4)1089 | 1115 | 98.3 | 92.3 | 1.52 | 30.8 | 0.391 | 0.150 | 0.170 | 0.290 |
|  | rpl20-rps12^ | 41 | 740(743.7±2.2)748 | 748 | 99.2 | 95.7 | 1.10 | 33.1 | 0.290 | 0.182 | 0.151 | 0.376 |
|  | rpoC1^ | 39 | 544 | 544 | 99.96 | 99.4 | 0.18 | 42.1 | 0.263 | 0.186 | 0.235 | 0.316 |
|  | rps16^ | 44 | 616(616±0.1)617 | 617 | 99.9 | 98.5 | 0.49 | 31.1 | 0.372 | 0.148 | 0.174 | 0.307 |
|  | trnC-ycf6^ | 42 | 889(893.3±8.9)932 | 971 | 98.4 | 88.2 | 1.03 | 29.6 | 0.321 | 0.135 | 0.187 | 0.357 |
|  | trnG-trnS^ | 43 | 638(654.3±16)688 | 700 | 97.1 | 87.6 | 1.86 | 27.9 | 0.397 | 0.129 | 0.170 | 0.304 |
|  | **psbA-trnH**^ | 40 | 258(272.8±7.5)285 | 320 | 91.9 | 74.1 | 3.35 | 17.4 | 0.332 | 0.075 | 0.129 | 0.446 |
|  | trnL-trnF^ | 43 | 936(938.6±2.8)942 | 949 | 99.3 | 96.7 | 0.84 | 30.4 | 0.364 | 0.156 | 0.151 | 0.329 |
|  | concatenated (outgroups included) | 48 | 9188(9627±171.9)10164 | 10570 | 84 | 21.9 | 1.84 | 29.3 | 0.309 | 0.139 | 0.149 | 0.306 |
|  | concatenated^ | 44 | 9285(9644.7±159.5)10164 | 10527 | 85.4 | 24.6 | 1.21 | 27.2 | 0.311 | 0.14 | 0.151 | 0.308 |
| **AT1** | all sequences | 462 | 1216 | 1216 | 98.9 | 48.9 | 20.72 | 41.4 | 0.276 | 0.136 | 0.278 | 0.310 |
|  | haplotypes | 399 | 1216 | 1216 | 98.8 | 49.2 | 20.15 | 41.4 | 0.274 | 0.137 | 0.277 | 0.312 |
|  | STR | 131 | 1216 | 1216 | 98.9 | 75.5 | 7.65 | 41.4 | 0.274 | 0.137 | 0.277 | 0.312 |
| **PEPC** | L + S-copes, total sequences | 317 | 670(797.3±42.5)853 | 902 | 84.9 | 20 | 38.36 | 39.1 | 0.283 | 0.217 | 0.225 | 0.276 |
|  | L + S-copes, haplotypes | 281 | 670(797.3±42.1)853 | 902 | 84.7 | 20.1 | 37.13 | 39.1 | 0.283 | 0.217 | 0.225 | 0.276 |
|  | L-copy (STR) | 125 | 670(818.5±16.1)853 | 900 | 97.1 | 32.8 | 5.22 | 39.9 | 0.280 | 0.21 | 0.229 | 0.281 |
|  | S-copy (STR) | 67 | 707(731±21.6)818 | 841 | 91.2 | 46.4 | 11.30 | 39.1 | 0.291 | 0.239 | 0.211 | 0.259 |
|  |  |  |  |  |  |  |  |  |  |  |  |  |
